# Supplementary material for: Evolutionary lineage-specific genomic imprinting at the ZNF791 locus
Source: PLoS Genet. 2025 Jan 15;21(1):e1011532. doi: 10.1371/journal.pgen.1011532 (PMC11734915; doi:10.1371/journal.pgen.1011532)
Supplement: S1 Fig — (PDF) [file pgen.1011532.s001.pdf]

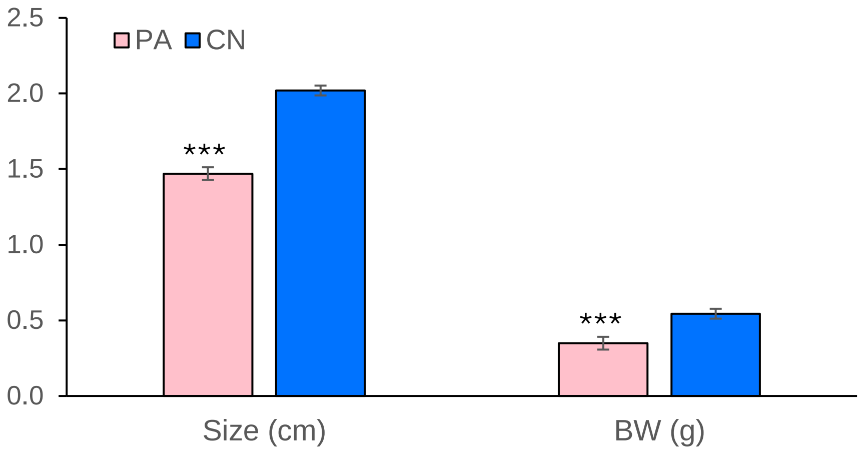

S1 Fig. Measurement of porcine embryos. The size and body weight (BW) of control (CN, n = 10) and parthenogenetically activated (PA, n = 10) embryos collected 21 days after the onset of estrus were measured. Data are presented as mean ± SEM.***, $P < 0.001$.
