# Supplementary material for: Evolutionary lineage-specific genomic imprinting at the ZNF791 locus
Source: PLoS Genet. 2025 Jan 15;21(1):e1011532. doi: 10.1371/journal.pgen.1011532 (PMC11734915; doi:10.1371/journal.pgen.1011532)
Supplement: S2 Fig — (PDF) [file pgen.1011532.s002.pdf]

A

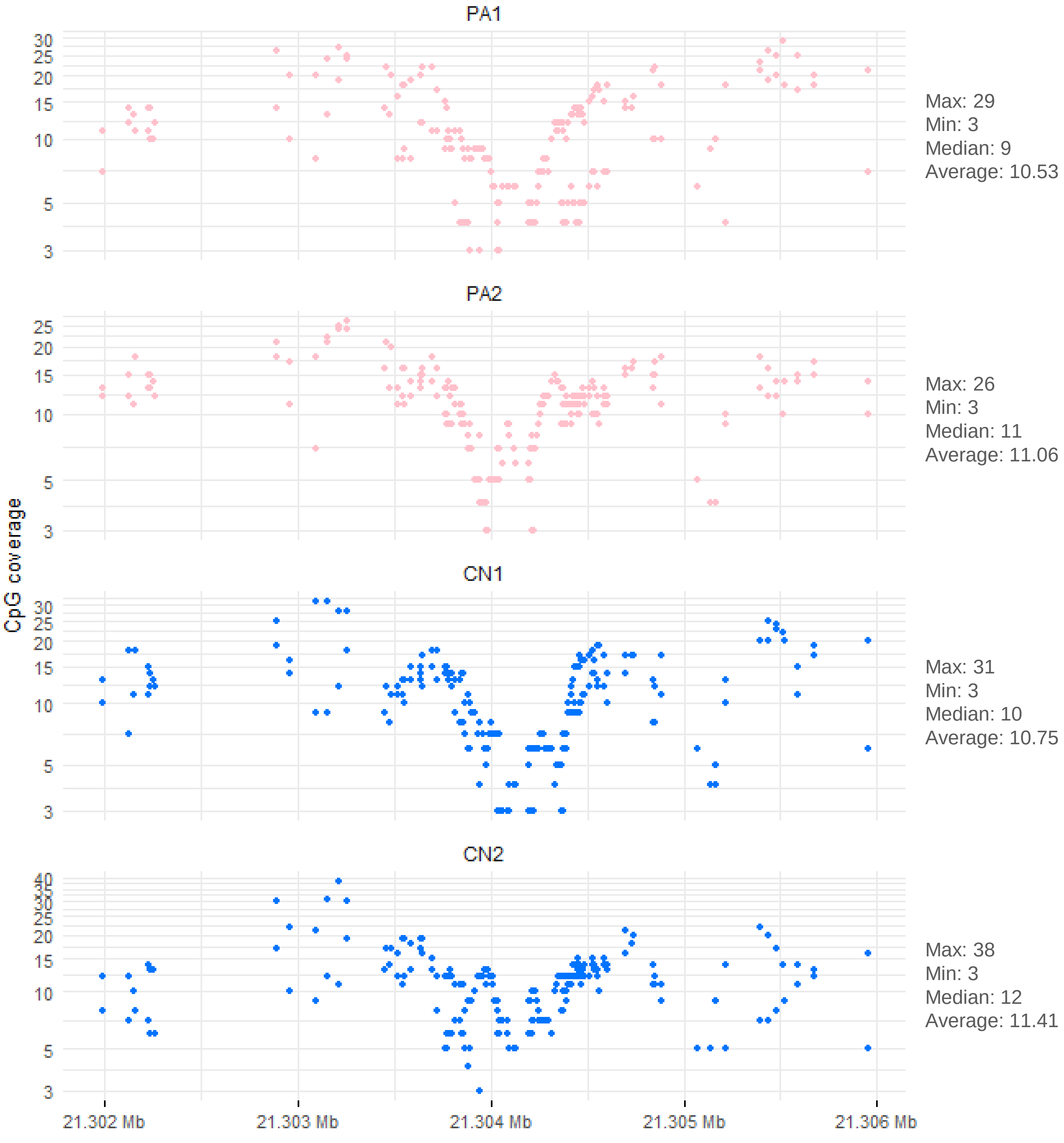

S2 Fig. CpG coverages and a positive control for the pig imprinted gene. (A) The CpG coverage for *PLAGL1*, presented in Fig 1C, is separated by each sample. Y-axis represents real numbers plotted on a log2 scale. The values on the right are based on XM_005654368.3, whose putative promoter region (TSS ± 2 kb) encompasses all CpGs within the depicted region (chr1:21301988-21305987) shown in Fig 1C.

B

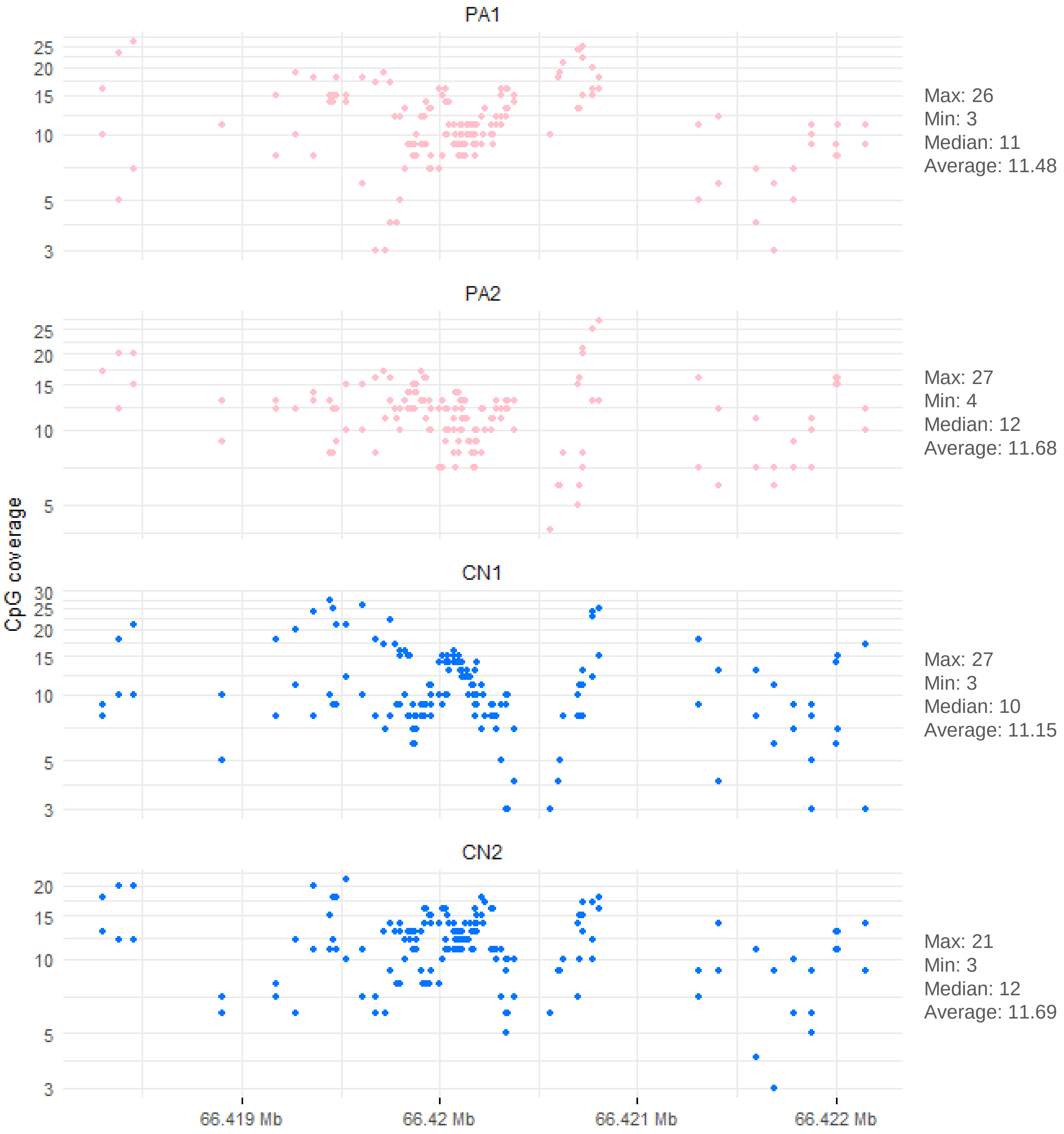

S2 Fig (Cont'd). (B) For *ZNF791-like*, the CpG coverage shown in Fig 1C is plotted for each sample. Y-axis represents real numbers on a log2 scale. The values on the right are based on XM_013990640.2, whose putative promoter region (TSS ± 2 kb) span the entire region (chr2:66418162-66422161) shown in Fig 1C.

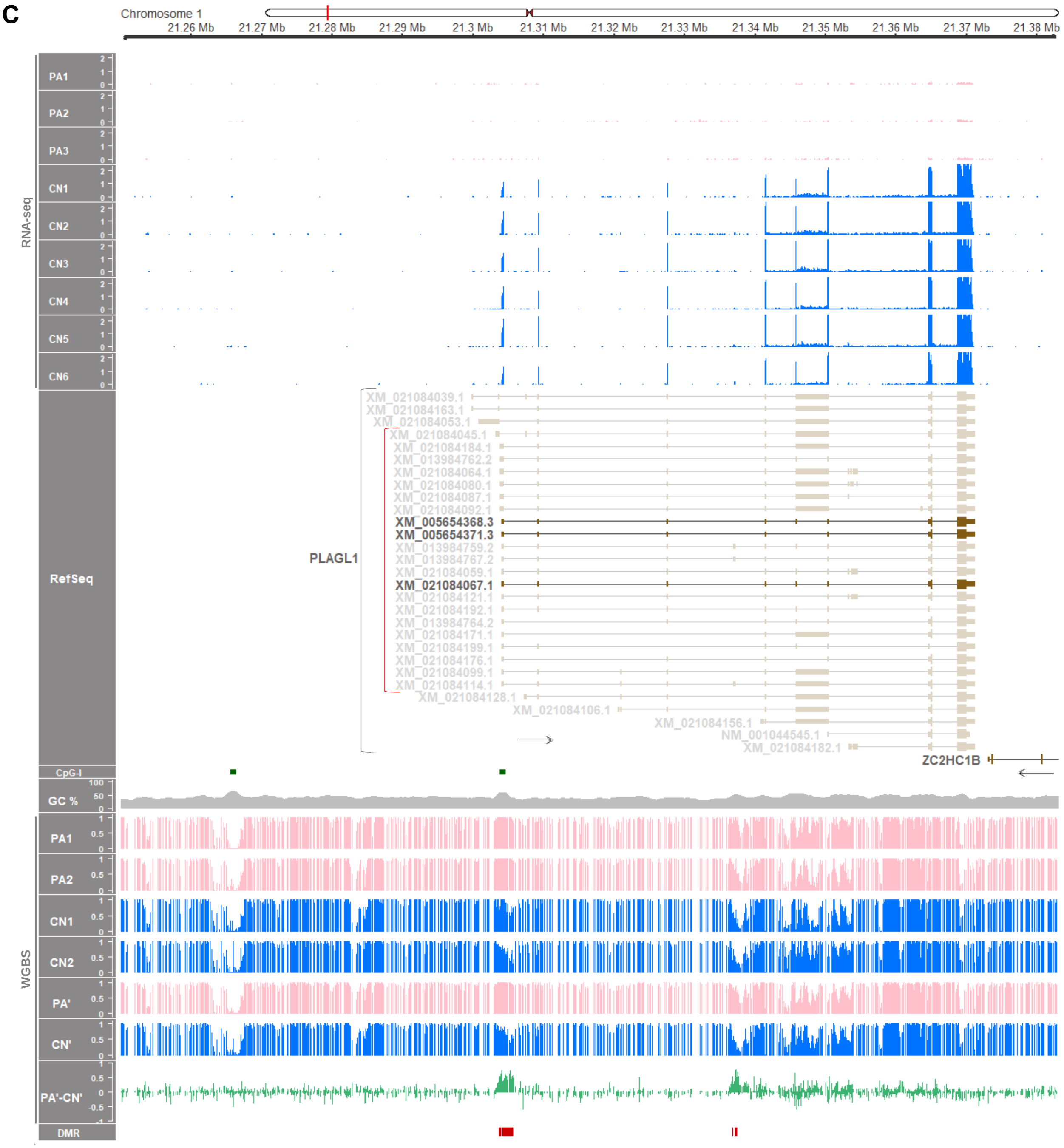

S2 Fig (Cont'd). (C) A positive control of imprinted expression in pig embryos. The known imprinted gene, PLAG1 like zinc finger 1 (*PLAGL1*), was expressed exclusively in CN embryos indicating its paternal expression. Maternally methylated DMRs were identified within the transcribed region. The transcript IDs are from the NCBI RefSeq annotation. RNA-seq read coverages and WGBS methylation levels are presented as TPM values and DNA methylation ratios, respectively. The candidate 21 transcripts associated with the DMR are marked with a red bracket. Non-expressed *PLAGL1* transcripts in the porcine embryonic samples analyzed in this study are covered with white shading for visual distinction.
