# Supplementary material for: Evolutionary lineage-specific genomic imprinting at the ZNF791 locus
Source: PLoS Genet. 2025 Jan 15;21(1):e1011532. doi: 10.1371/journal.pgen.1011532 (PMC11734915; doi:10.1371/journal.pgen.1011532)
Supplement: S3 Fig — (PDF) [file pgen.1011532.s003.pdf]

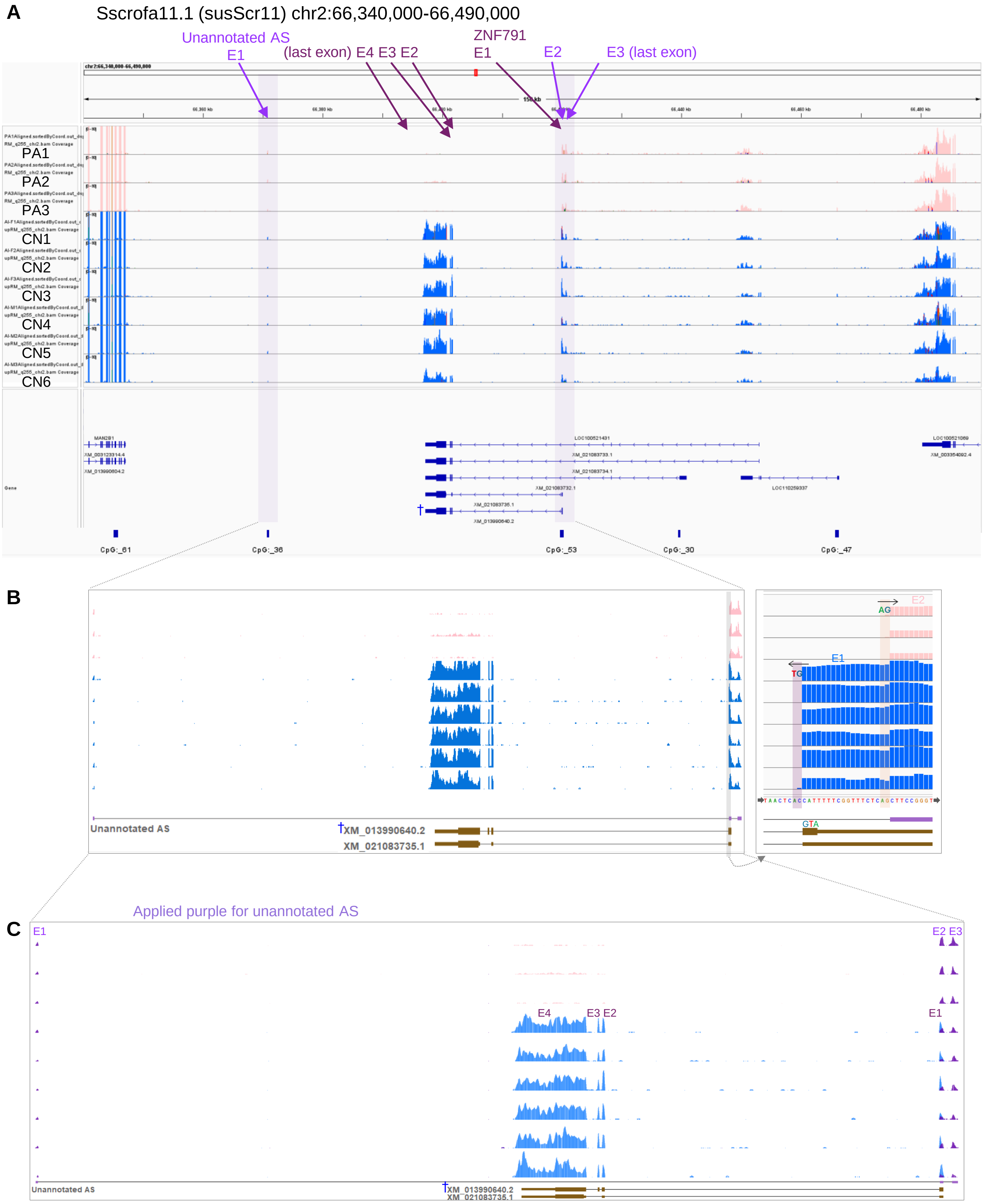

S3 Fig. Identification of expressed transcripts from RNA-seq data of porcine PA and CN embryos. (A) The pig *ZNF791-like* locus is shown with Integrative Genomics Viewer (IGV). **(B)** Close views of expressed transcripts. Splicing donor (GT) and acceptor (AG) are denoted with directional arrows. The start codon (ATG) is marked. **(C)** Visualization of expression and overlapping of the *ZNF791-like* transcript (blue) and Unannotated AS (purple). A predominant *ZNF791-like* transcript is marked with †.
