# Supplementary material for: Evolutionary lineage-specific genomic imprinting at the ZNF791 locus
Source: PLoS Genet. 2025 Jan 15;21(1):e1011532. doi: 10.1371/journal.pgen.1011532 (PMC11734915; doi:10.1371/journal.pgen.1011532)
Supplement: S4 Fig — (PDF) [file pgen.1011532.s004.pdf]

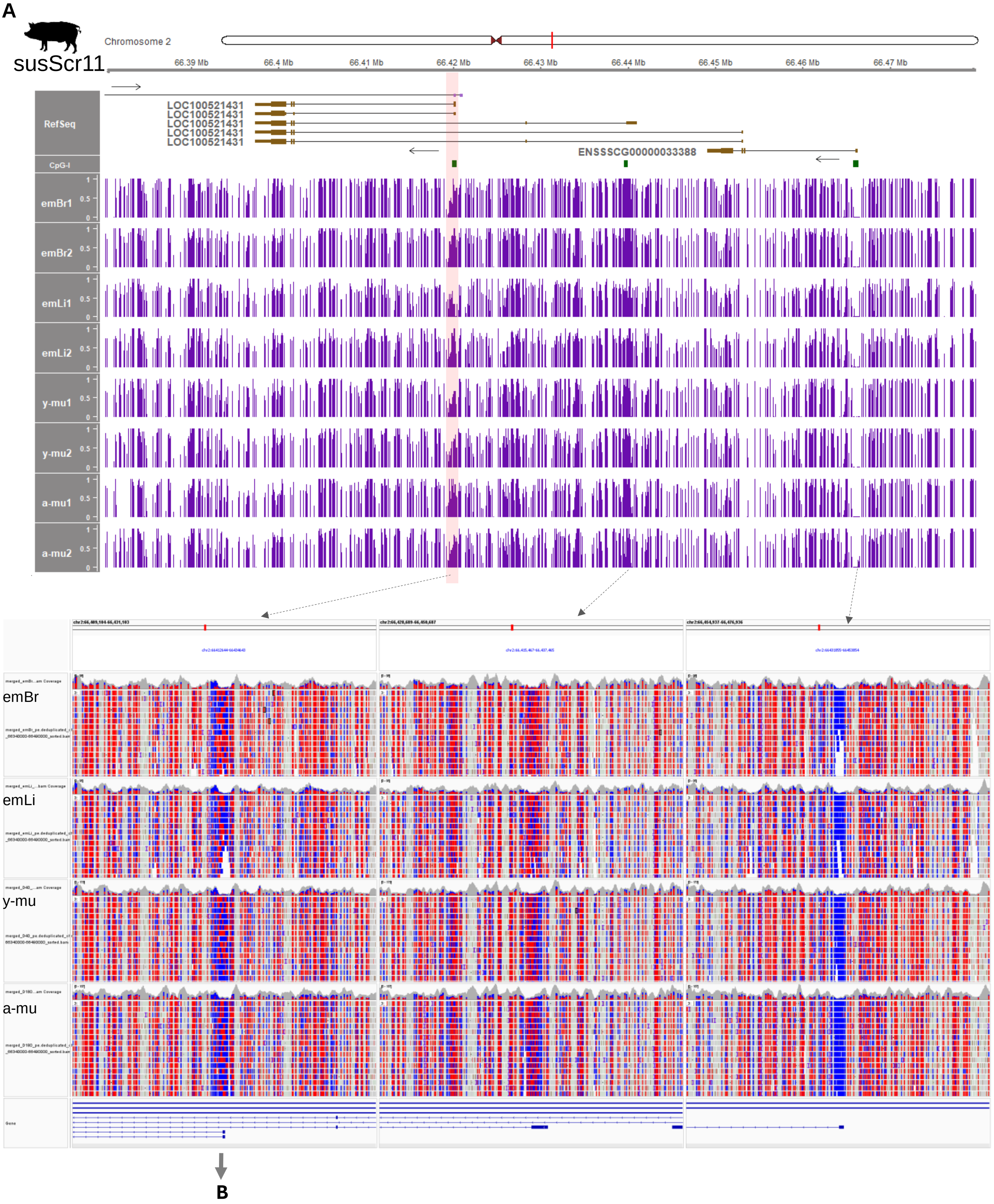

S4 Fig. Partial DNA methylation at the *ZNF791* locus in pigs downstream of the *MAN2B1* gene. (A) In the split-screen view of merged reads displayed at the bottom, the red color represents unconverted (methylated) cytosines, and the blue color represents bisulfite-converted (unmethylated) cytosines. The CpG sites are displayed in either red or blue.

B

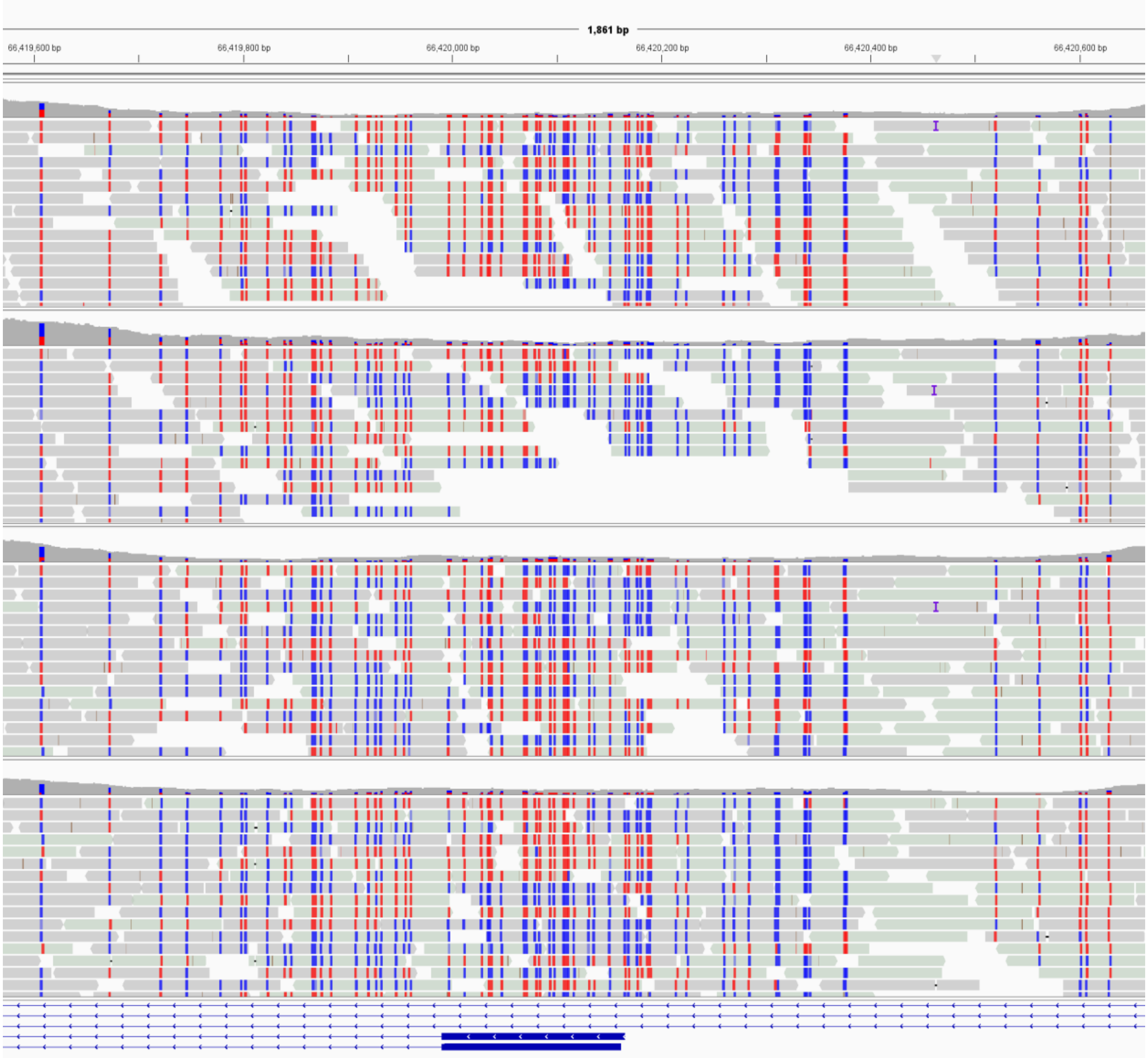

S4 Fig (Cont'd). (B) A close view of the partially methylated region indicated in A around chr2:66,420,000 (66.42 Mb), which is highlighted with a red bar. This region was analyzed for hemi-methylation tendency and status as described in Figures 3C and 3D. The same approach was applied for other hemi-methylation analyses in the following S5 Fig.– S10 Fig.

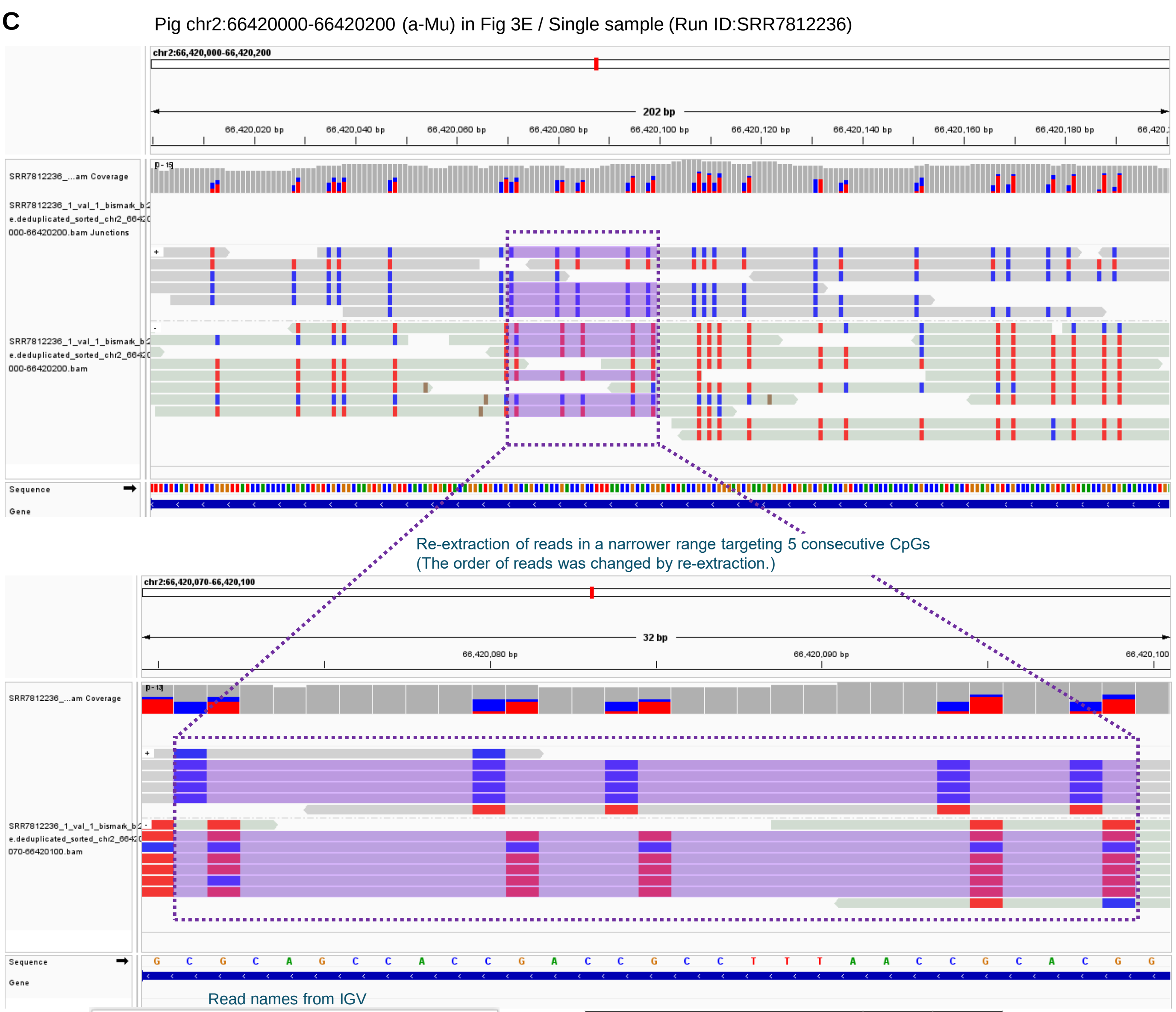

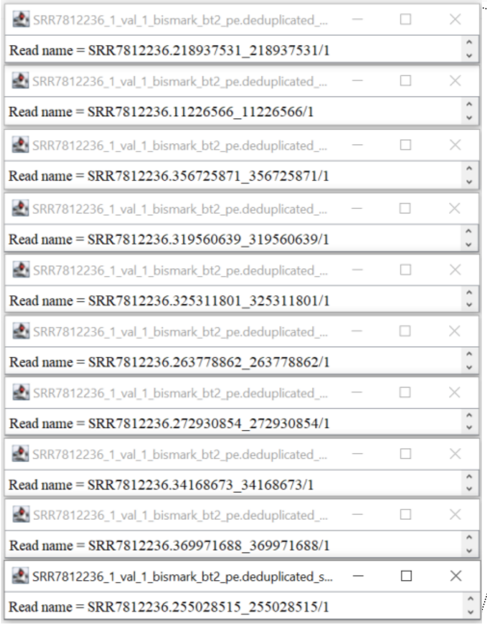

readname	chr	start	
SRR7812236.325311801_325311801/1	chr2	66419959	Qualified reads
SRR7812236.263778862_263778862/1	chr2	66419980	
SRR7812236.218937531_218937531/1	chr2	66419983	
SRR7812236.272930854_272930854/1	chr2	66420000	
SRR7812236.11226566_11226566/1	chr2	66420003	
SRR7812236.34168673_34168673/1	chr2	66420027	
SRR7812236.356725871_356725871/1	chr2	66420032	
SRR7812236.319560639_319560639/1	chr2	66420037	
SRR7812236.369971688_369971688/1	chr2	66420058	
SRR7812236.255028515_255028515/1	chr2	66420066	
SRR7812236.331622760_331622760/1	chr2	66419932	Unqualified reads
SRR7812236.325311801_325311801/1	chr2	66419938	
SRR7812236.330086736_330086736/1	chr2	66419986	
SRR7812236.283171849_283171849/1	chr2	66419995	
SRR7812236.90544603_90544603/1	chr2	66420009	
SRR7812236.125521342_125521342/1	chr2	66420074	
SRR7812236.135546090_135546090/1	chr2	66420088	
SRR7812236.270155337_270155337/1	chr2	66420090	

S4 Fig (Cont'd). (C) Procedures for analyzing pig chr2:66420000-66420200 (a-Mu) presented in Fig 3E. This example of a local search for qualified reads from a BAM file was conducted for a single sample (Run ID: SRR7812236). For a targeted region within the dotted boxes (chr2:66420070-66420100), IGV screenshots show 5 consecutive CpGs on either the + strand or – strand across 10 reads shaded in purple. These purple-shaded reads from IGV were matched with those extracted by MethylDackel v0.6.1 using the BAM file to identify qualified and unqualified reads for subsequent analysis. For IGV, the default mapping quality threshold of 30 was used.

D QUMA input procedure

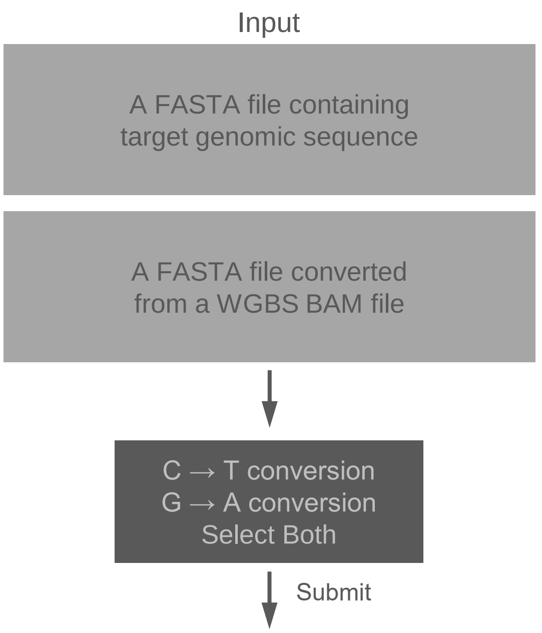

E QUMA result

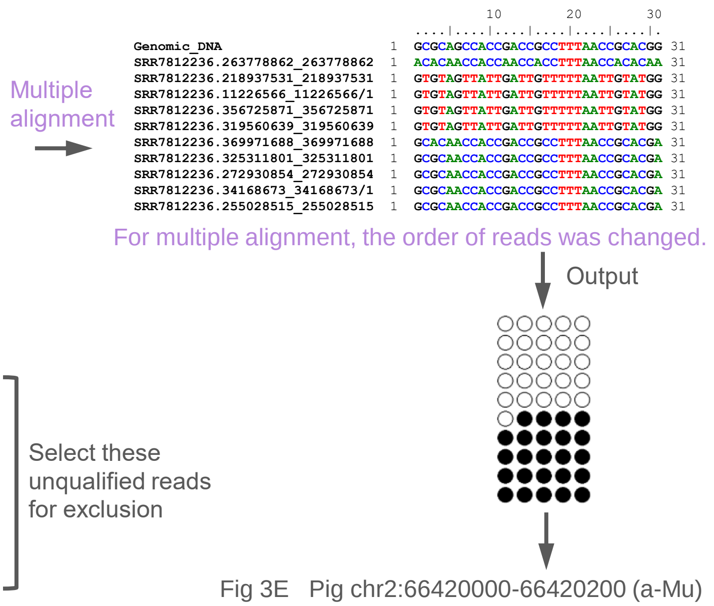

readname	chr	start
SRR7812236.325311801_325311801/1	chr2	66419959
SRR7812236.263778862_263778862/1	chr2	66419980
SRR7812236.218937531_218937531/1	chr2	66419983
SRR7812236.272930854_272930854/1	chr2	66420000
SRR7812236.11226566_11226566/1	chr2	66420003
SRR7812236.34168673_34168673/1	chr2	66420027
SRR7812236.356725871_356725871/1	chr2	66420032
SRR7812236.319560639_319560639/1	chr2	66420037
SRR7812236.369971688_369971688/1	chr2	66420058
SRR7812236.255028515_255028515/1	chr2	66420066
SRR7812236.331622760_331622760/1	chr2	66419932
SRR7812236.325311801_325311801/1	chr2	66419938
SRR7812236.330086736_330086736/1	chr2	66419986
SRR7812236.283171849_283171849/1	chr2	66419995
SRR7812236.90544603_90544603/1	chr2	66420009
SRR7812236.125521342_125521342/1	chr2	66420074
SRR7812236.135546090_135546090/1	chr2	66420088
SRR7812236.270155337_270155337/1	chr2	66420090

S4 Fig (Cont'd). (D) Input procedures of QUMA (quantification tool for methylation analysis; http://quma.cdb.riken.jp/). Two input files were used: i) a target genomic sequence in FASTA format and ii) a FASTA file generated from an aligned WGBS BAM file, which can be prepared using "samtools fasta .bam -o .fa". DNA methylation on both strands was selected for analysis. The analysis was initiated by clicking "Submit". **(E)** QUMA results. After the analysis using the default settings, unqualified reads shown in S4C Fig were selected and excluded from the displayed results. Multiple alignment results were generated, and a CpG methylation pattern plot was produced, which was used in Fig 3E. Open and solid circles represent unmethylated and methylated CpGs, respectively.
