# Supplementary material for: Evolutionary lineage-specific genomic imprinting at the ZNF791 locus
Source: PLoS Genet. 2025 Jan 15;21(1):e1011532. doi: 10.1371/journal.pgen.1011532 (PMC11734915; doi:10.1371/journal.pgen.1011532)
Supplement: S6 Fig — (PDF) [file pgen.1011532.s006.pdf]

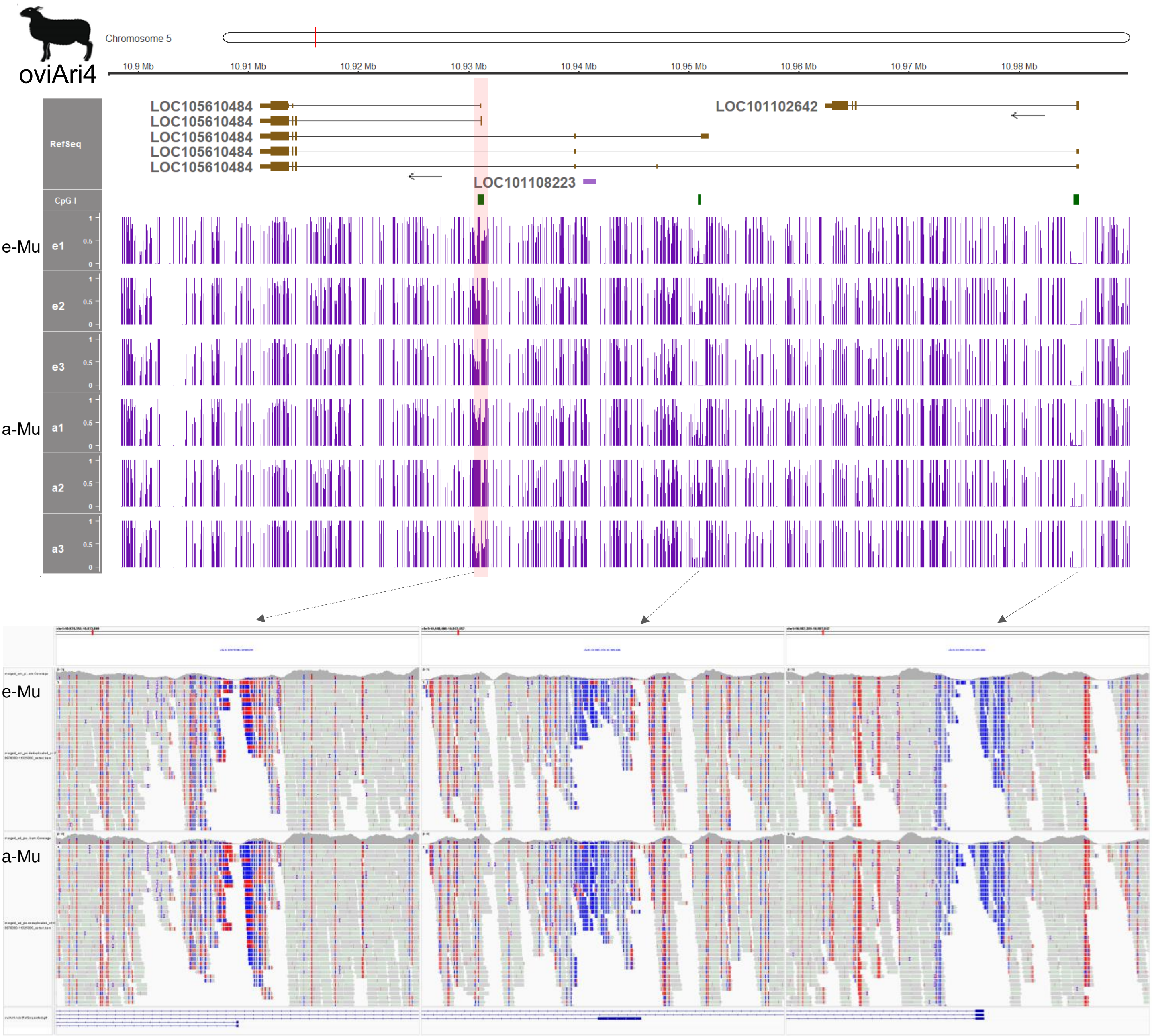

S6 Fig. Partial DNA methylation at the *ZNF791* locus in sheep downstream of the *MAN2B1* gene. Split screen view of merged reads are displayed at the bottom where red represents unconverted (methylated) and blue represents bisulfite-converted (unmethylated) cytosines. The CpG sites are displayed in either red or blue. e-Mu, embryonic muscle; a-Mu, adult muscle.
