# Supplementary material for: Evolutionary lineage-specific genomic imprinting at the ZNF791 locus
Source: PLoS Genet. 2025 Jan 15;21(1):e1011532. doi: 10.1371/journal.pgen.1011532 (PMC11734915; doi:10.1371/journal.pgen.1011532)
Supplement: S7 Fig — (PDF) [file pgen.1011532.s007.pdf]

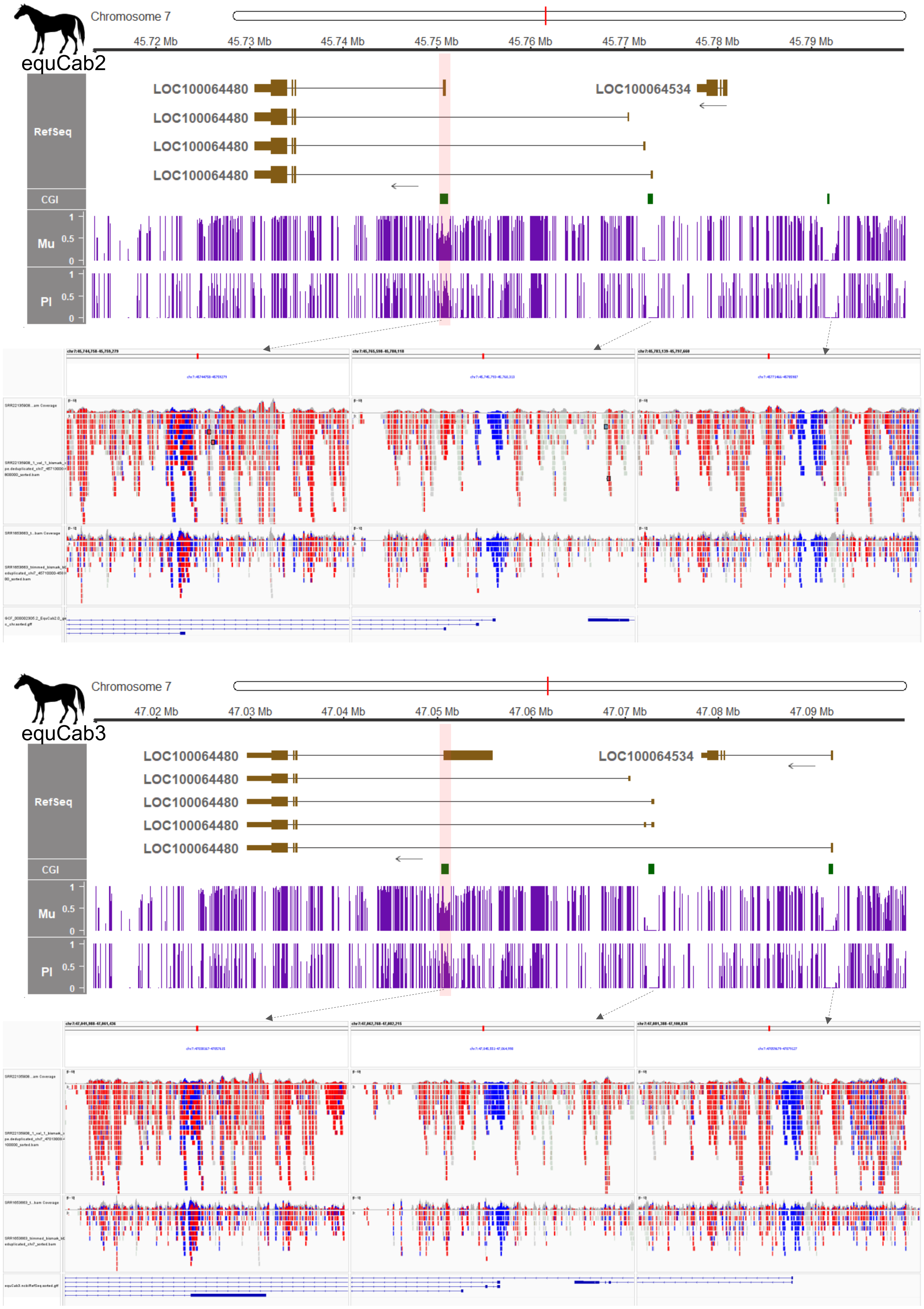

equCab2
Chromosome 7
45.72 Mb
45.73 Mb
45.74 Mb
45.75 Mb
45.76 Mb
45.77 Mb
45.78 Mb
45.79 Mb
RefSeq
LOC100064480
LOC100064534
LOC100064480
LOC100064480
LOC100064480
CGI
Mu
Pl
1
0.5
0
equCab3
Chromosome 7
47.02 Mb
47.03 Mb
47.04 Mb
47.05 Mb
47.06 Mb
47.07 Mb
47.08 Mb
47.09 Mb
RefSeq
LOC100064480
LOC100064534
LOC100064480
LOC100064480
LOC100064480
LOC100064480
CGI
Mu
Pl
1
0.5
0

S7 Fig. Partial DNA methylation at the *ZNF791* locus in horses downstream of the *MAN2B1* gene. Split screen view of merged reads are displayed at the bottom where red represents unconverted (methylated) and blue represents bisulfite-converted (unmethylated) cytosine. The CpG sites are displayed in either red or blue. The same data from horse skeletal muscle used in Fig 3 were aligned to both the current reference genome (EquCab3.0/equCab3) and the previous (EquCab2.0/equCab2) genome.
