# Supplementary material for: Evolutionary lineage-specific genomic imprinting at the ZNF791 locus
Source: PLoS Genet. 2025 Jan 15;21(1):e1011532. doi: 10.1371/journal.pgen.1011532 (PMC11734915; doi:10.1371/journal.pgen.1011532)
Supplement: S8 Fig — (PDF) [file pgen.1011532.s008.pdf]

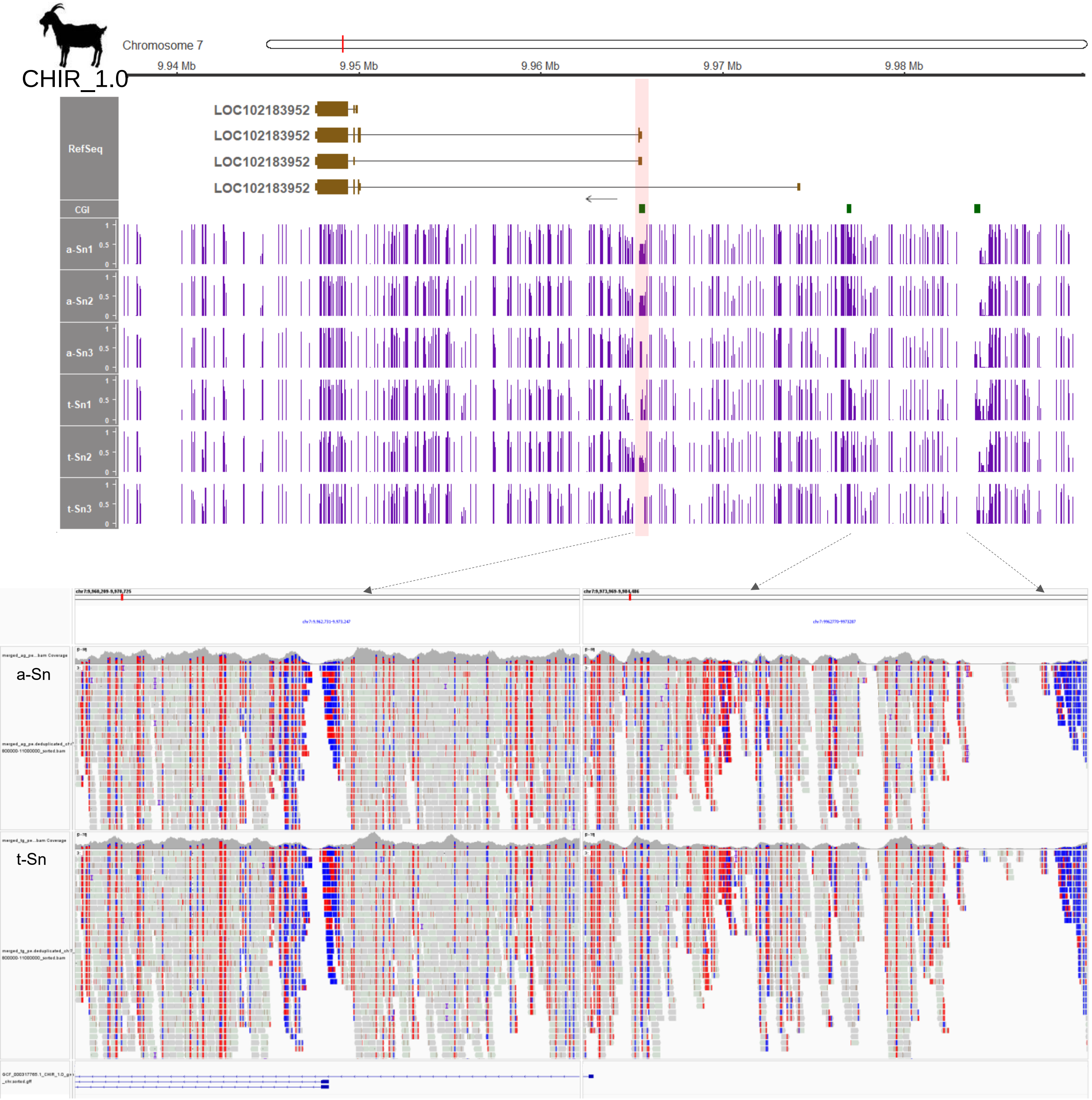

S8 Fig. Partial DNA methylation at the *ZNF791* locus in goat downstream of the *MAN2B1* gene. Split screen view of merged reads are displayed at the bottom (red represents unconverted (methylated) and blue represents bisulfite-converted (unmethylated). The CpG sites are displayed in either red or blue.
