# Supplementary material for: Evolutionary lineage-specific genomic imprinting at the ZNF791 locus
Source: PLoS Genet. 2025 Jan 15;21(1):e1011532. doi: 10.1371/journal.pgen.1011532 (PMC11734915; doi:10.1371/journal.pgen.1011532)
Supplement: S9 Fig — (PDF) [file pgen.1011532.s009.pdf]

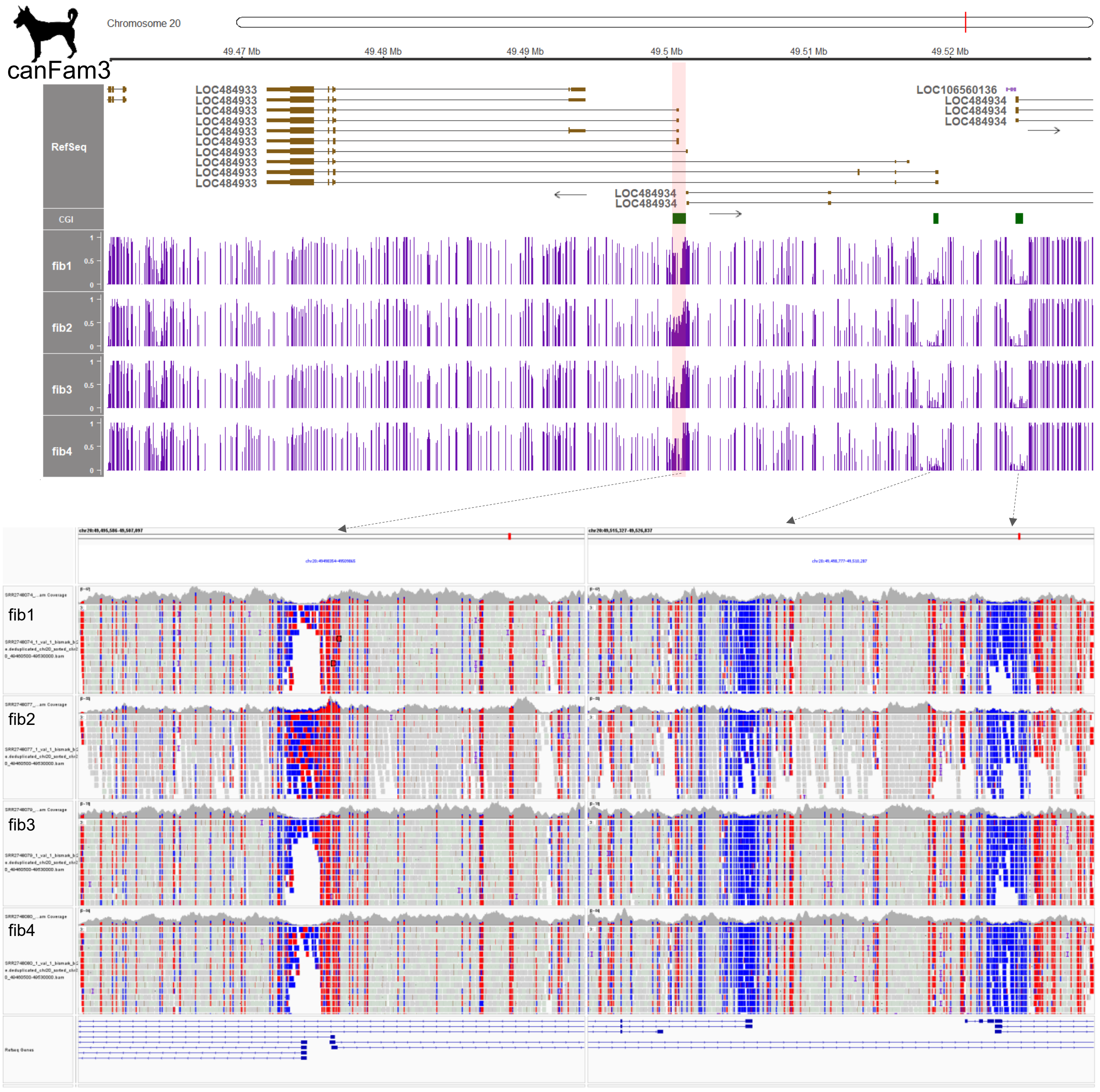


**S9 Fig. Partial DNA methylation at the *ZNF791* locus in dogs downstream of the *MAN2B1* gene.** Split screen view of sample reads are displayed, where red represents unconverted (methylated) and blue represents bisulfite-converted (unmethylated) cytosines. The CpG sites are displayed in either red or blue.
