# Supplementary material for: Evolutionary lineage-specific genomic imprinting at the ZNF791 locus
Source: PLoS Genet. 2025 Jan 15;21(1):e1011532. doi: 10.1371/journal.pgen.1011532 (PMC11734915; doi:10.1371/journal.pgen.1011532)
Supplement: S10 Fig — (PDF) [file pgen.1011532.s010.pdf]

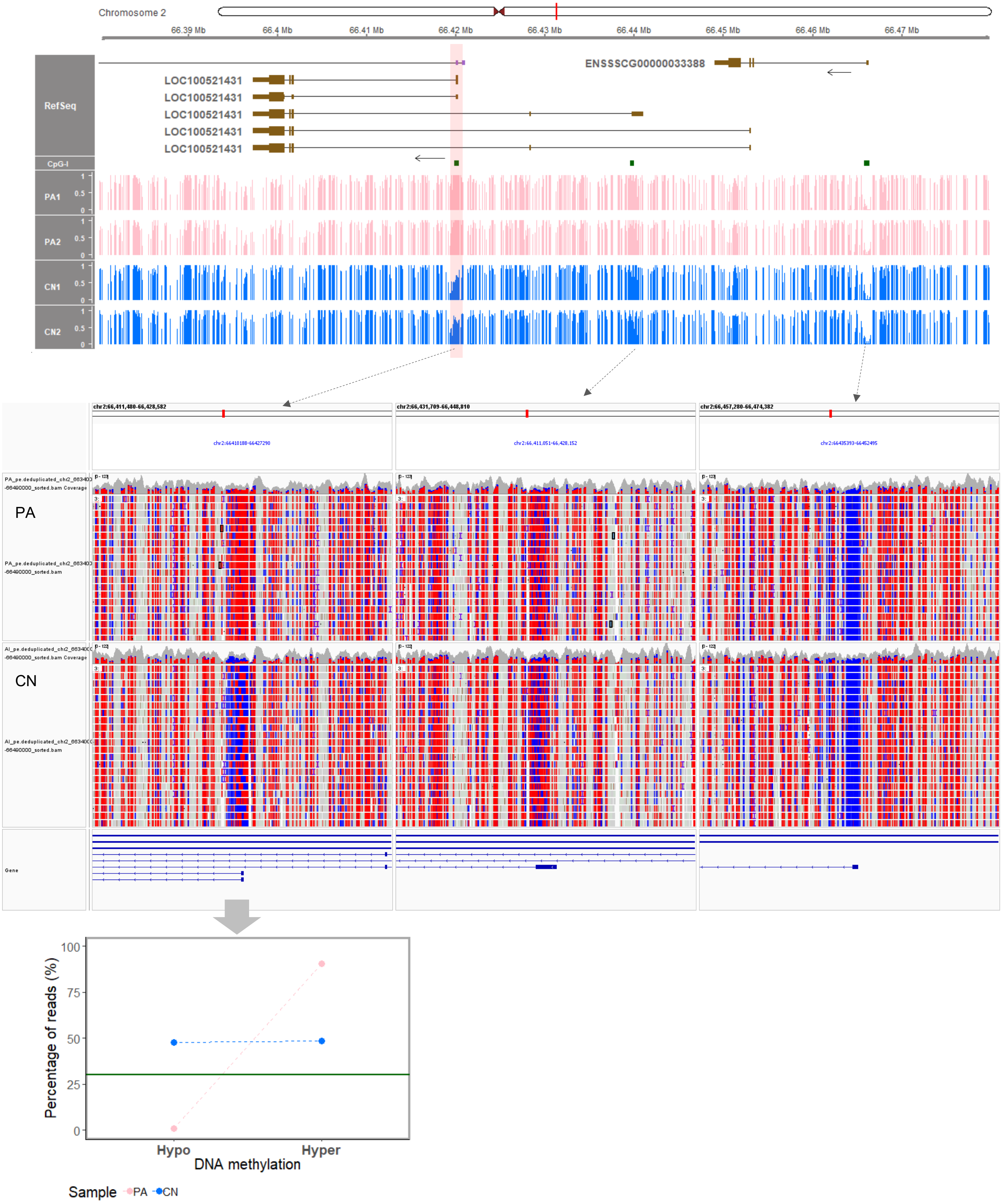

S10 Fig. DNA methylation at the *ZNF791* locus in pig embryos downstream of the *MAN2B1* gene. In the split-screen view of merged reads displayed in the middle, the red color represents unconverted (methylated) cytosines, and the blue color represents bisulfite-converted (unmethylated) cytosines. The CpG sites are displayed in either red or blue. At the bottom, the full methylated region in PA embryos exhibited hypermethylation only, while the partially methylated in CN embryos showed hemi-methylation tendency.
