# Supplementary material for: Evolutionary lineage-specific genomic imprinting at the ZNF791 locus
Source: PLoS Genet. 2025 Jan 15;21(1):e1011532. doi: 10.1371/journal.pgen.1011532 (PMC11734915; doi:10.1371/journal.pgen.1011532)
Supplement: S12 Fig — (PDF) [file pgen.1011532.s012.pdf]

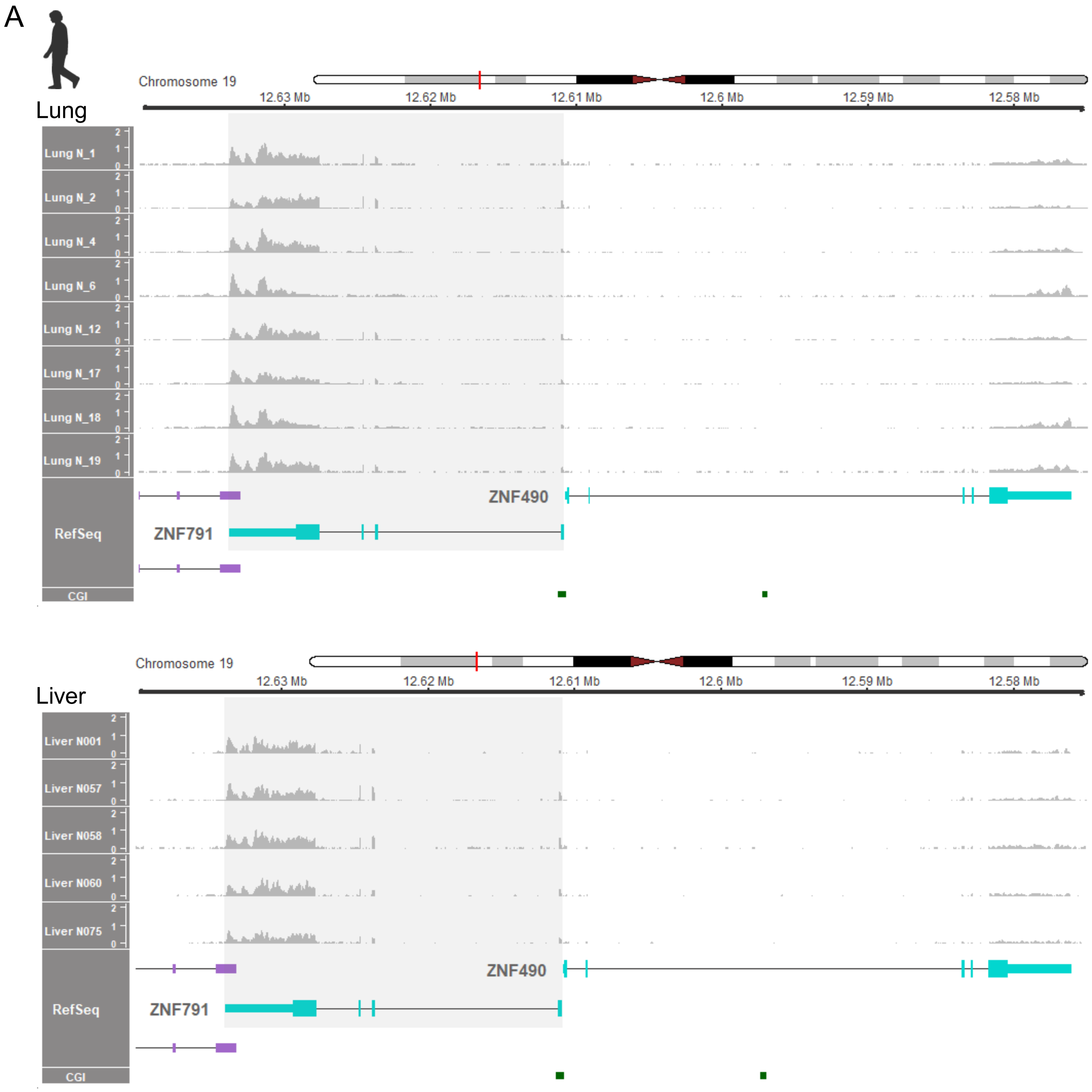

S12 Fig. Expressed *ZNF791* transcripts in humans, primates, and mice. (A) Human *ZNF791* mRNA expression in lung and liver tissues.

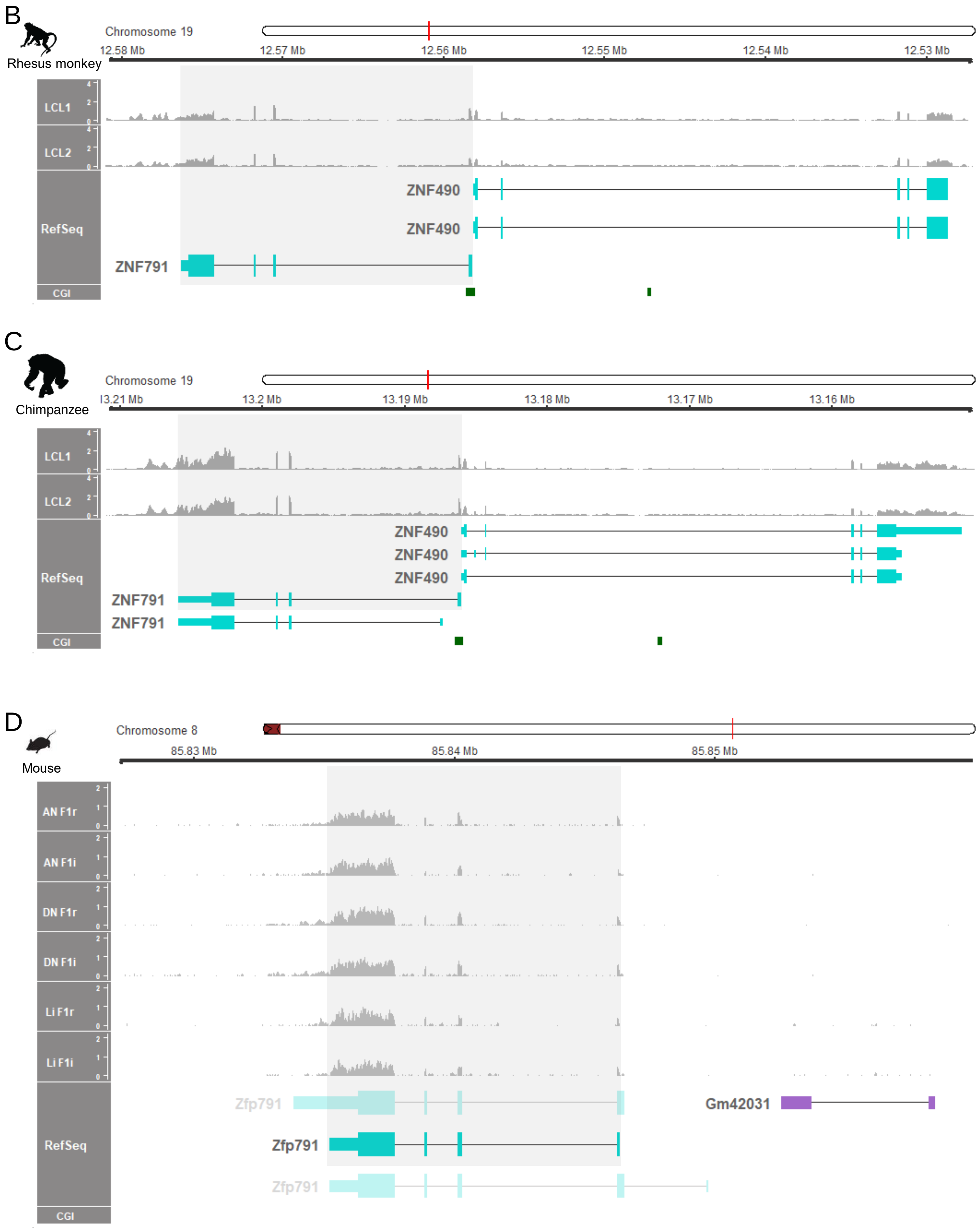

S12 Fig (Cont'd). (B, C) Primate *ZNF791* mRNA expression. **(D)** Mouse *Zfp791* mRNA expression patterns. Transcripts that are expressed are indicated with grey highlights. RNA-seq read coverages were normalized to TPM. Datasets used in Figure 4 (PRJNA395106 for human lung, hum0158.v2 for human liver, PRJNA563344 for rhesus monkey and chimpanzee, and GSE70484 for mouse) are analyzed.
