# Supplementary material for: Evolutionary lineage-specific genomic imprinting at the ZNF791 locus
Source: PLoS Genet. 2025 Jan 15;21(1):e1011532. doi: 10.1371/journal.pgen.1011532 (PMC11734915; doi:10.1371/journal.pgen.1011532)
Supplement: S13 Fig — (PDF) [file pgen.1011532.s013.pdf]

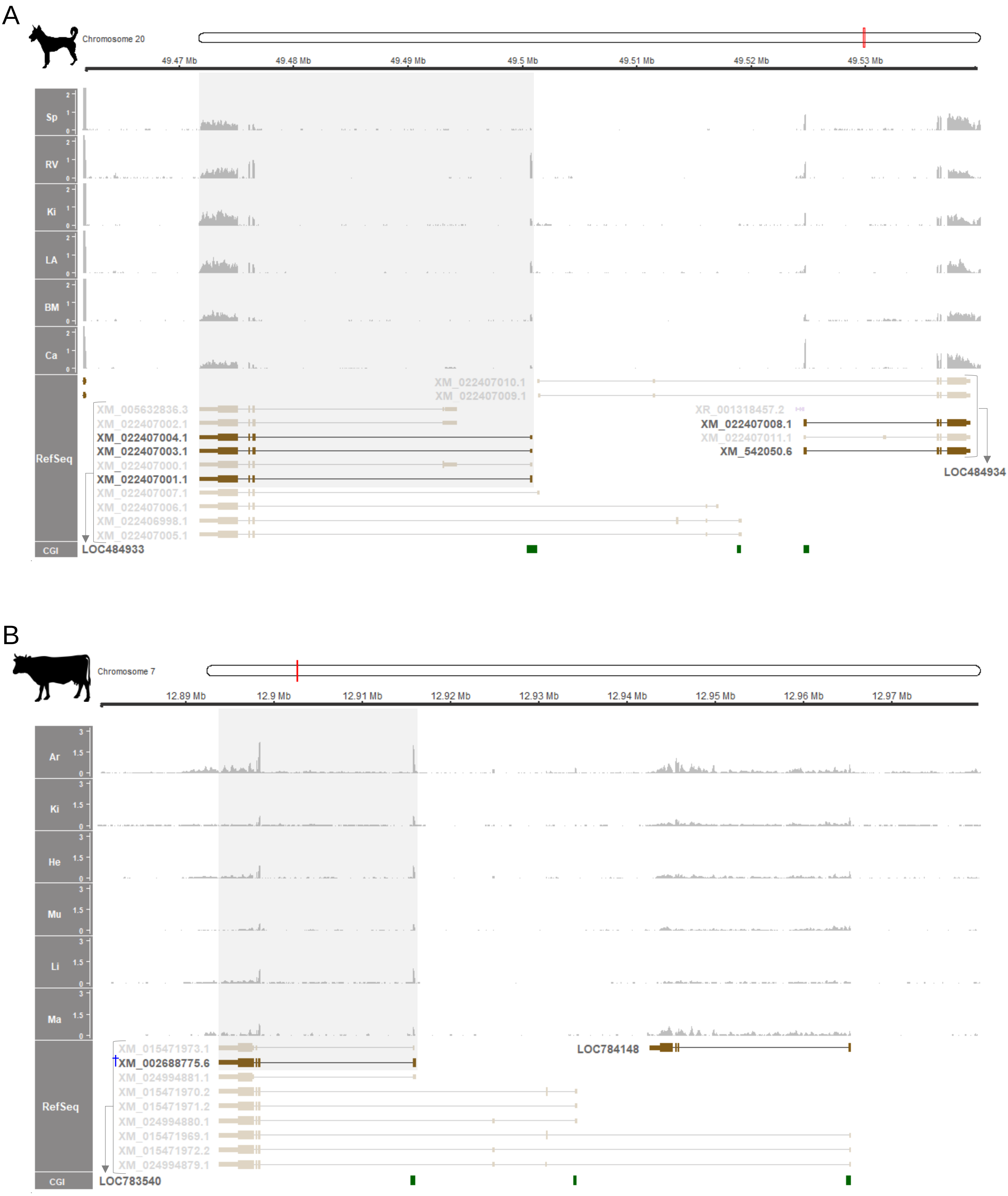

S13 Fig. Expressed *ZNF791* transcripts in dogs, cattle, and pigs. (A) Dog *ZNF791* mRNA expression. **(B)** Cattle *ZNF791* mRNA expression.

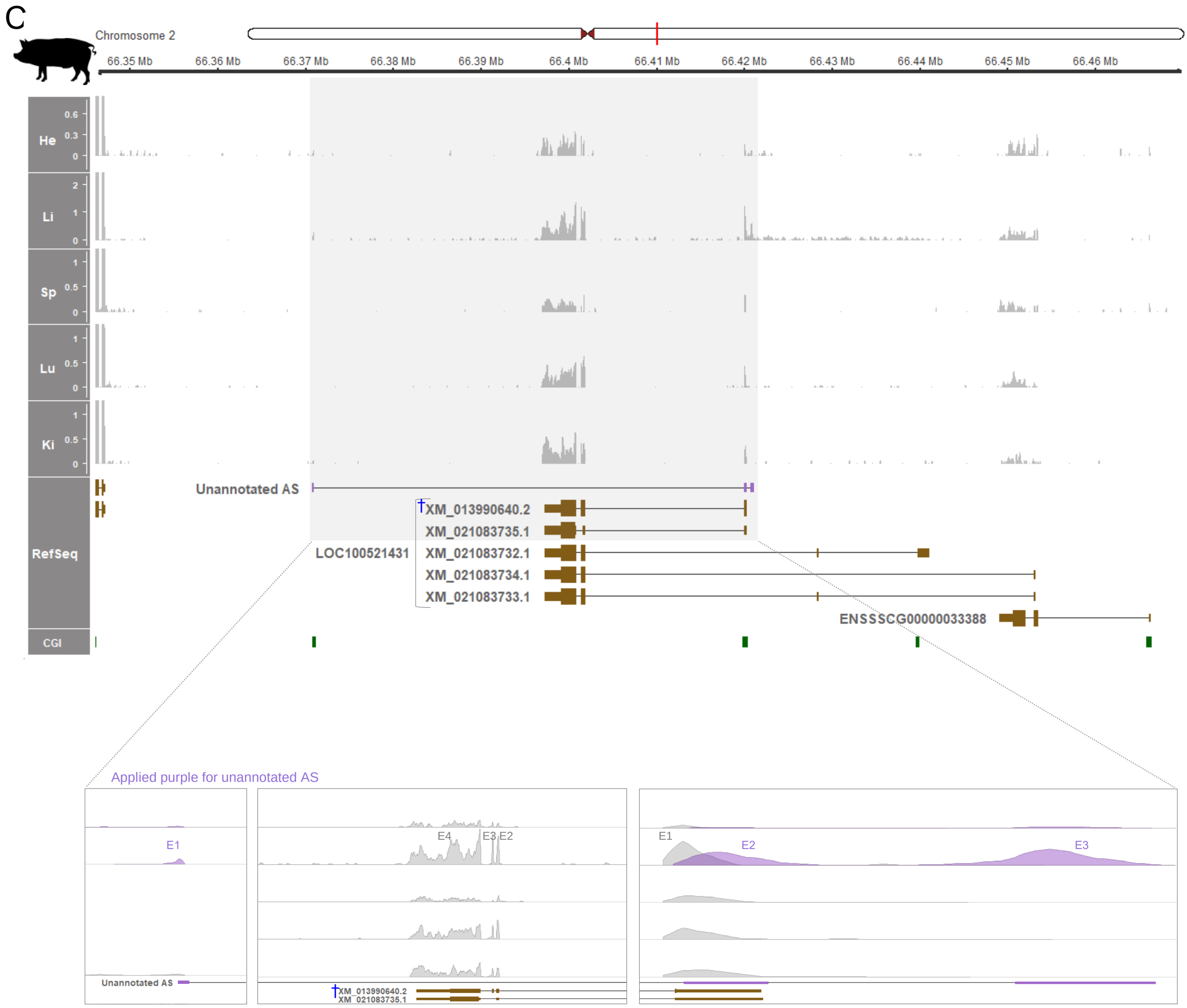

S13 Fig (Cont'd). (C) Pig *ZNF791* mRNA expression patterns. Transcripts that are expressed are indicated with grey highlights. RNA-seq read coverages were normalized to TPM. Datasets used in Figure 4 (PRJNA396033 for dog, ERP118133 for cow, and GSE77776 for pig (Jinhua)) are analyzed. The major predominant *ZNF791* transcript is marked with a blue symbol (†).

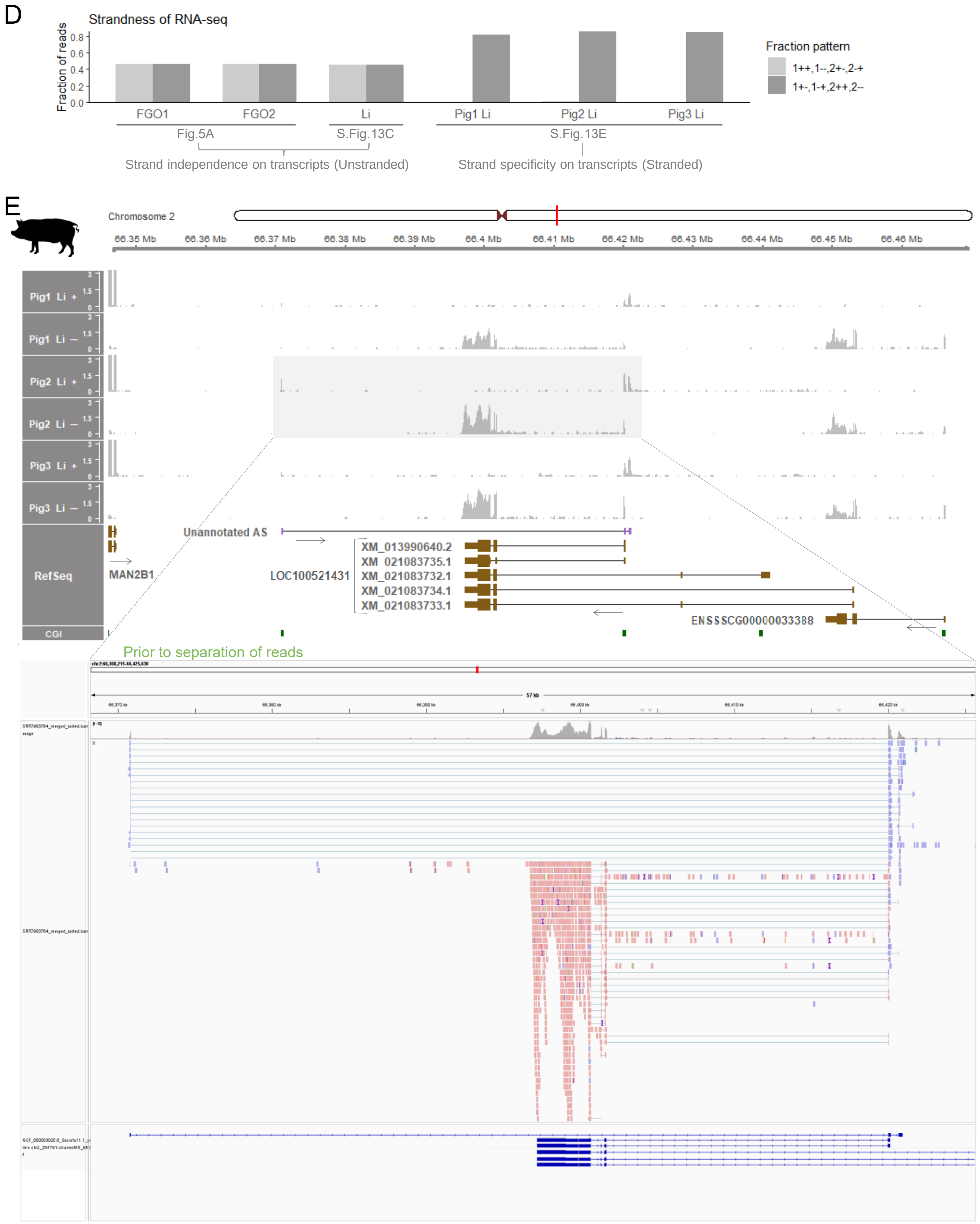

S13 Fig (Cont'd). (D) The strand specificity of RNA-seq reads was assessed using the infer_experiment.py script from the RSeQC package (https://rseqc.sourceforge.net/). The strandness of the reads was either independent of the transcript strand annotation, indicating unstranded RNA-seq, or dependent on it, indicating stranded RNA-seq. **(E)** Analysis of stranded RNA-seq data from liver tissues of three 180-day-old Large White pigs (PRJNA493166). BAM files were analyzed using bamCoverage from deepTools with the parameters --filterRNAstrand forward or --filterRNAstrand reverse to capture transcript directionality. Forward (+) and reverse (−) reads are presented separately. Prior to the separation of reads, alignments were colored by the first-of-pair strand in IGV, with red and blue denoting different read orientations.
