# Supplementary material for: Evolutionary lineage-specific genomic imprinting at the ZNF791 locus
Source: PLoS Genet. 2025 Jan 15;21(1):e1011532. doi: 10.1371/journal.pgen.1011532 (PMC11734915; doi:10.1371/journal.pgen.1011532)
Supplement: S14 Fig — (PDF) [file pgen.1011532.s014.pdf]

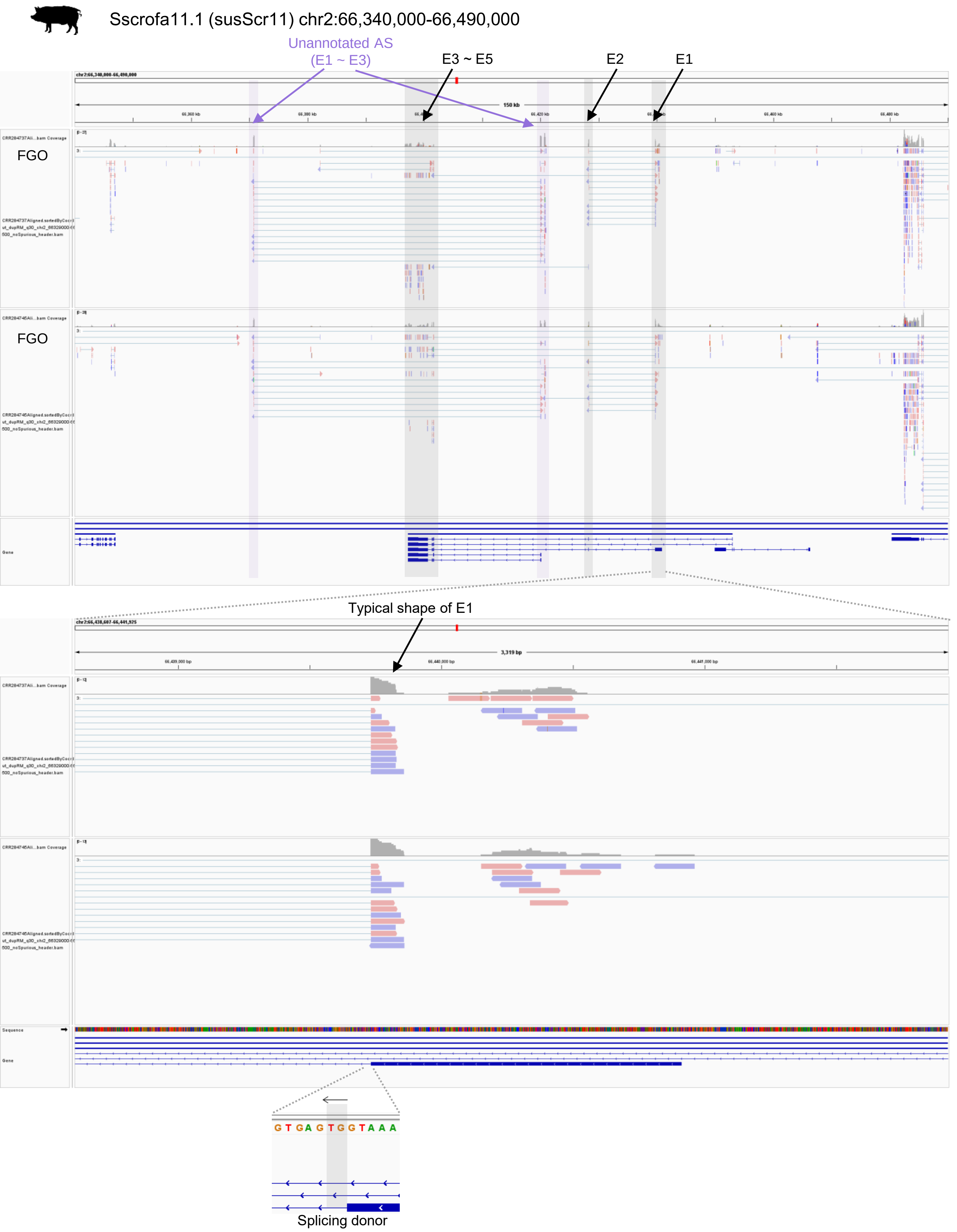

S14 Fig. Expressed transcripts at the *ZNF791* locus in pig oocytes. The 1st, 2nd and last exons of expressed *ZNF791* transcript in pig oocytes are indicated with grey highlights. Zoomed view of the 1st exon region. The splicing donor (GT) is shown. The exon1 could be shorter than the annotated one because the typical shape of exon1 is shorter.
