# Supplementary figures and images for: Evolutionary lineage-specific genomic imprinting at the ZNF791 locus
Source: PLoS Genet. 2025 Jan 15;21(1):e1011532. doi: 10.1371/journal.pgen.1011532 (PMC11734915; doi:10.1371/journal.pgen.1011532)

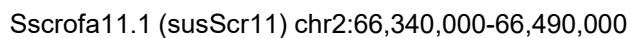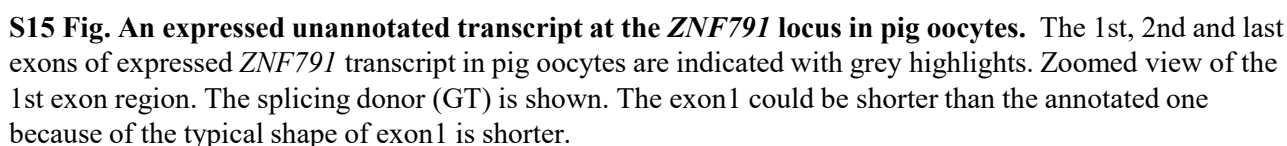

Supplement: S15 Fig — (PDF) [file pgen.1011532.s015.pdf]
