# Supplementary material for: Evolutionary lineage-specific genomic imprinting at the ZNF791 locus
Source: PLoS Genet. 2025 Jan 15;21(1):e1011532. doi: 10.1371/journal.pgen.1011532 (PMC11734915; doi:10.1371/journal.pgen.1011532)
Supplement: S16 Fig — (PDF) [file pgen.1011532.s016.pdf]

S16 Fig. Dfam database search process. The Dfam database was queried with input sequence from each species. These sequences were searched for transposable elements (TEs) specific to each organism using profile hidden Markov models (HMMs). This process was carried out in the following order:

1. Dfam database (https://www.dfam.org/home)
2. Pig (*Sus scrofa*) sequence
3. Cattle (*Bos taurus*) sequence
4. Sheep (*Ovis aries*) sequence
5. Horse (*Equus caballus*) sequence
6. Goat (*Capra hircus*) sequence
7. Dog (*Canis lupus familiaris*) sequence
8. Summary of gene and LTR orientations
9. Comparison of gene and LTR annotations in NCBI, UCSC, Dfam, and Ensembl for pigs

1. Dfam database (https://www.dfam.org/home)

1.1. Sequence search

Dfam release 3.8

1.2. FASTA-formatted consensus LTR52 sequence, deposited in the Dfam database

```
>DF000000543.4 LTR52
TGTAATAAAGAGTCTGACTCCATTTTTTGATGTTTGACTGCTGACAGCTTTTAAGCCTCA
CCCCTCCCTCTTCCCTTTGCCCCACATCTGGGCAAGCTGATAAGAAAGCCCGGGTGCTCC
CTCCTTTGGCGCCGGCGGGAAGTTCAAACCACGCAAGCCCCTGCCTGCGGGAACCCTCAC
CCCGGCCCCACCCCCNTAACCACAATAAAAACCCCAAGCCAGTCTCCTTTCCCTGCTCTC
TCAAGCCATTTTCGGACCTGCTTGGGAGGCCTGCCCTGCTCTCCCCAGAAAGCCTCANTT
ATGTGAGTAATAAACCTTTTCATACCCTCTTGGTGCGTGTGTGGCATCATCAGTCTCGAC
ATCCGAACCAAATTTTGGGTGGGGGAGTCCATCCTGCCTCTGCAGGGTGACCACAACAAC
A
```

2. Pig (*Sus scrofa*) sequence

2.1. Sequence search input (*ZNF791* E1 + 1kb upstream)

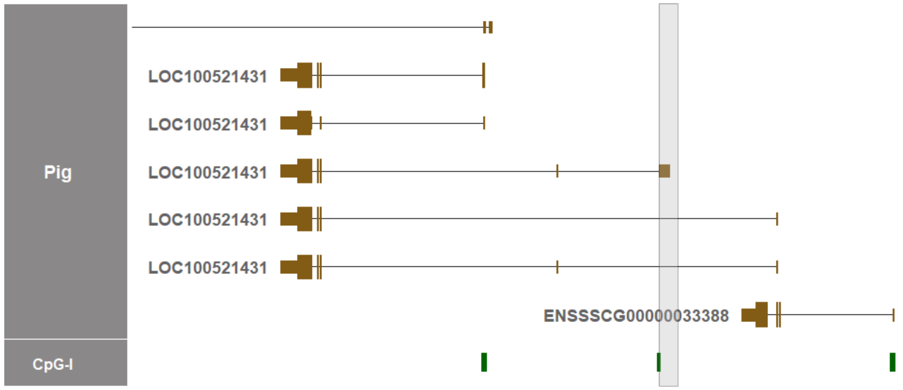

>chr2:66439732-66441912
CCATTTCCCCGCTTCTGGGATGCTCCGGCGTCACACCTTAAACTTCTACGACCCAAACAG
CTCACGGCAGGAATAACGCTGCAGCTGACAAACCTGGGCCGCTTAAGGTCCGTTGCGACT
TGACGCCAAATCTGGCCAGGAGGGTCCACGTGGTTTGTGGAGCATCGCCCAACCCACCTA
CCCCTGCTGCGTCCGTGATTGGACAATTCGCAAGCACCCGCCCCCTTTTCCTGAGTGAAG
TAGGTCTGGAGGTCACGTGATTGATCCCAccagcttctttccttaaacgTGATTGTGTAT
CTTAGAGAGCGACGTTTGCTTTTAAAACCACACTTGTTCCTGGCCCTAGAAGATGTGGCC
GTCTTCCTCAGATTTTTCAGAACGCGGGTACAGTGGTGTGCGCAGGGAATTCCAGAGTCA
AATTCTAGAAGAACCAACATACTTGTCCAAAGCTAGAGGGTCGCCTGGGTCTGGCCAACT
GGGACGATAGTTGGCATAGGCTCCGAAGCCACTCGTGGTACTGTGACCCTTGTTTACAAA
CACACTGAAAAATAACCTCACCACGTGTAATGAAGAGTCTAGCTCCATTTCAGTCTTTGT
TGACAGGGTTGAGATGTCCACACATCTGCTTTTCCCCTTCCATACACGAGGTATTTAAGT
TAATCTCCTGGCCTCCCAAGCACTTGACTCTGCTGAGAAATTTGAGCCGTGCAGCCCTCG
CTTGGGAACAGGGACTCTTCTACCAGCCGTAAGTTGTAAGATGTGTTCAGTAATACTCAG
TATACATTTTATCCTacaaggaaatttattttaattttgtagtgTGTGCCTAGAGAGTTT
GAAATGTAAATGGCTATGATATATTTAGGTCAATCCAGTTACTATTTTCACCAGTATAAC
AAAAACCAGCCAAAAGTTATGGAAATGTCGGATACCTGCAAACTTTTAATATTCTTAACA
CATTGGTTCAAAACTTCCAGtgcatttatttatcttaataGTTTTACTGAGGTAAAATAC
ACATACCATACACTGCATCCCTTAAATGattacaattcagtggttttcagtGTATTCACA
GGTATGCGCAATCAGCACCACAATTTTTGAACATTACTCTCACTTCTAAAAGAAACCCCT
GAAGGAGAGAGACAGTctcatcgggttaaggacctgacattgtctccctgaggatgcagg
ttcaatccctggttttgctcagtgggttaaggatccagcgttgccacaagctttggtgta
ggtaacagatgatacttggatccagtgtggctgtggctgtggcatagcctcagctgcagc
tcagattcaaccccccaccccaccccacccccagtaagggaacttccatatgatgcaggtg
ggccataaaaaggaaaaagaaaaaaagagaaaccccTGCCCCCTTTAGAAGTTCTTCTCT
ATCTTCCTATCTTCCTCCAAGCTACAATTAGACAACCTCTAATCTTTGTTTCTGTAGATT
TCCCTATTCTTCATTTACATATGAATGGACTCACATAATGTATTGTCTTTGTAATTGACT

```
CTGGTTGCTTAGCATAACTTTTTCAAGAAACATGTATgctgagttccctgatggcctagt
ggctaaaaaaaatttcaaaattcaaaccaaaataaaccataaaaaaagaaacatctatgC
TGTAGCATGTATGGATACCTATTTGtctttatggccaaataatattctactgtatggata
aaacatatttattcacCCCTTAATGGATTTACTTTGTTCCTacgttttggctattatgaa
taatgttgcaataaaCTTTCATGGAGAAGTTTTTGTTTAGAcgtattttcatttctattt
ggcaGATTCCTAGGAATGAATTGATGAGTCTTAAGGTAATTCTAGGTTTAATTGCTAAGG
AACTTTTCCCCaaaagactgttttccaaagtggctgcaccattttatattcccaccagca
gtgggtGAGTGTTCCAGTTTTTCCAAATCATCCACAAAACTtattatctcacttttttttt
tttttgtatttttttagggcaacacctggaatatggaagttcccagtctactCAGAaccga
actgcagctgccagcctaagccacagccacagcaatgccagatctaagcaatgctggata
cttacccactgagtgaggaca
```

2.2. Dfam results from profile HMMs

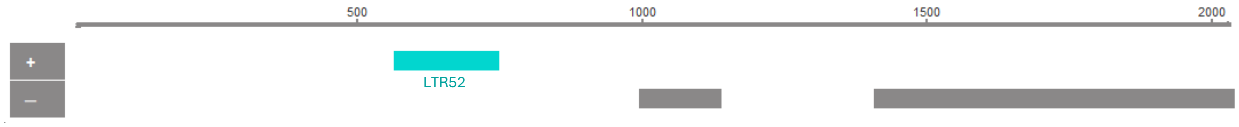

# target name	acc	query name	bits	e-value	bias	hmm-st	hmm-en	strand	ali-st	ali-en	env-st	env-en	modlen	description of target
# -------------------	-------------------	-------------------	------	---------	-----	-------	-------	------	-------	-------	-------	-------	-------	---------------------
LTR52	DF000000543.4	chr2:66439732-66441912	21.5	3.7e-05	2.3	1	188	+	566	749	566	763	421	LTR52 (Long Terminal Repeat) for ERVL endogenous retrovirus
L1MB8_3end	DF000000274.4	chr2:66439732-66441912	87.7	1.1e-25	12.0	774	921	-	1139	995	1143	988	925	3' end of L1 retrotransposon, L1MB8_3end subfamily
L1MD3_3end	DF000000289.4	chr2:66439732-66441912	258.3	9.2e-78	48.6	222	782	-	2040	1408	2058	1398	2742	3' end of L1 retrotransposon, L1MD3_3end subfamily

Using *Sus scrofa* as a query organism and the Dfam curated threshold (--cut_ga), the LTR52 was detected within the query input as shown in cyan.

```
>chr2:66439732-66441912
CCATTTCCCCGCTTCTGGGATGCTCCGGCGTCACACCTTAAACTTCTACGACCCAAACAG
CTCACGGCAGGAATAACGCTGCAGCTGACAAACCTGGGCCGCTTAAGGTCCGTTGCGACT
TGACGCCAAATCTGGCCAGGAGGGTCCACGTGGTTTGTGGAGCATCGCCCAACCCACCTA
CCCCTGCTGCGTCCGTGATTGGACAATTCGCAAGCACCCGCCCCCTTTTCCTGAGTGAAG
TAGGTCTGGAGGTCACGTGATTGATCCCAccagcttctttccttaaacgTGATTGTGTAT
CTTAGAGAGCGACGTTTGCTTTTAAAACCACACTTGTTCCTGGCCCTAGAAGATGTGGCC
GTCTTCCTCAGATTTTTCAGAACGCGGGTACAGTGGTGTGCGCAGGGAATTCCAGAGTCA
AATTCTAGAAGAACCAACATACTTGTCCAAAGCTAGAGGGTCGCCTGGGTCTGGCCAACT
GGGACGATAGTTGGCATAGGCTCCGAAGCCACTCGTGGTACTGTGACCCTTGTTTACAAA
CACACTGAAAAATAACCTCACCACGTGTAATGAAGAGTCTAGCTCCATTTCAGTCTTTGT
TGACAGGGTTGAGATGTCCACACATCTGCTTTTCCCCTTCCATACACGAGGTATTTAAGT
```

TAATCTCCTGGCCTCCCAAGCACTTGACTCTGCTGAGAAATTTGAGCCGTGCAGCCCTCG
CTTGGGAACAGGGACTCTTCTACCAGCCGTAAGTTGTAAGATGTGTTCAGTAATACTCAG
TATACATTTTATCCTacaaggaaatttattttaattttgtagtgTGTGCCTAGAGAGTTT
GAAATGTAAATGGCTATGATATATTTAGGTCAATCCAGTTACTATTTTCACCAGTATAAC
AAAAACCAGCCAAAAGTTATGGAAATGTCGGATACCTGCAAACTTTTAATATTCTTAACA
CATTGGTTCAAAACTTCCAGtgcatttatttatcttaataGTTTTACTGAGGTAAAATAC
ACATACCATACACTGCATCCCTTAAATGattacaattcagtggttttcagtGTATTCACA
GGTATGCGCAATCAGCACCACAATTTTTGAACATTACTCTCACTTCTAAAAGAAACCCCT
GAAGGAGAGAGACAGTctcatcgggttaaggacctgacattgtctccctgaggatgcagg
ttcaatccctggttttgctcagtgggttaaggatccagcgttgccacaagctttggtgta
ggtaacagatgatacttggatccagtgtggctgtggctgtggcatagcctcagctgcagc
tcagattcaacccccaccccaccccacccccagtaagggaacttccatatgatgcaggtg
ggccataaaaaggaaaaagaaaaaaagagaaaccccTGCCCCCTTTAGAAGTTCTTCTCT
ATCTTCCTATCTTCCTCCAAGCTACAATTAGACAACCTCTAATCTTTGTTTCTGTAGATT
TCCCTATTCTTCATTTACATATGAATGGACTCACATAATGTATTGTCTTTGTAATTGACT
CTGGTTGCTTAGCATAACTTTTTCAAGAAACATGTATgctgagttccctgatggcctagt
ggctaaaaaaaatttcaaaattcaaaccaaaataaaccataaaaaaagaaacatctatgC
TGTAGCATGTATGGATACCTATTTGtctttatggccaaataatattctactgtatggata
aaacatatttattcacCCCTTAATGGATTTACTTTGTTCCTacgttttggctattatgaa
taatgttgcaataaaCTTTCATGGAGAAGTTTTTGTTTAGAcgtattttcatttctattt
ggcaGATTCCTAGGAATGAATTGATGAGTCTTAAGGTAATTCTAGGTTTAATTGCTAAGG
AACTTTTCCCCaaaagactgttttccaaagtggctgcaccattttatattcccaccagca
gtgggtGAGTGTTCCAGTTTTTCCAAATCATCCACAAAACTtattatctcacttttttttt
tttttgtatttttttagggcaacacctggaatatggaagttcccagtctactCAGAaccga
actgcagctgccagcctaagccacagccacagcaatgccagatctaagcaatgctggata
cttacccactgagtgaggaca

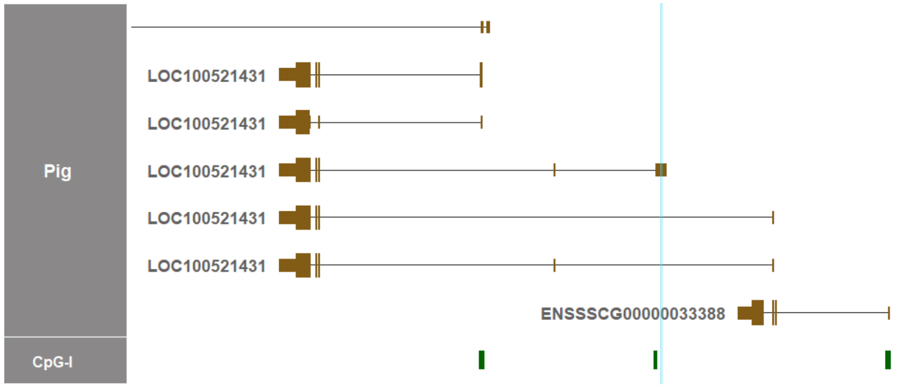

The detected region of LTR52 is indicated in cyan in both the sequence above and the genome visualizer.

3. Cattle (*Bos taurus*) sequence

3.1. Sequence search input (*ZNF791* E1 + 1kb upstream)

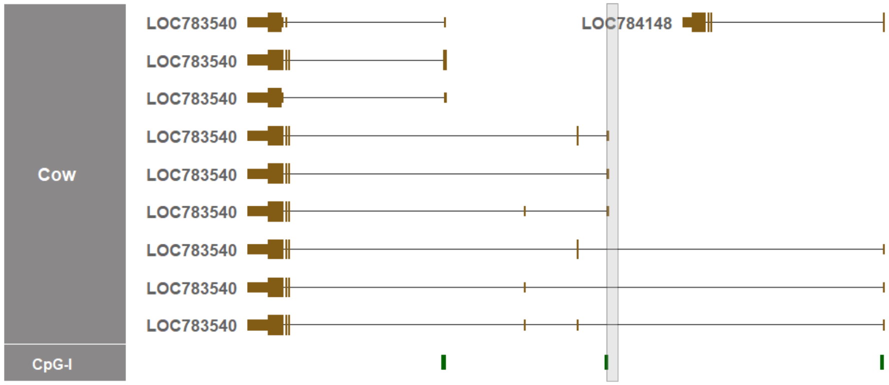


```
>chr7:12934124-12935308
CACTTCACGCCTTCCGGGGTTCTCCGGCGTTATTCCTTAGGCTCACGCGGCCTCTGCAGC
TCATAGCGGTAATGAAGCTGCAGCGAACTAACCTGAGCTGCTTCAACACGGCGACACTCC
ACGCAGAGCGTGCCGGGAGAGGCCGACCACACGGTTTGCGGGACGTGGCCCCACCCACCT
TCCCCTTCTGCGGTCCTGATTGGACAGTTCGCAAGCAACGCCCCCTTTATCCTGAGTAAT
AATGGATCCAGAGGCCACGTGCCTGAGCAGAATCAGCTTCTGCCGGTGGGTAGAAACCTA
CCCCTCTCCAGggacttttgtttttaaaccactCTTGTTACTTGTCCTAGAGGGACAGTG
GCAACTTCCTCAGATACTTCAGAACGTGGGGACACAGGTGTGCACAAGGAATTCCAGAGT
CAGATCCCAGAAGAGCCGACGTTCTTGTCCAAAGCAAGAGCAGGCCAACTGGAAAAACAG
GGTCTGAGGGTCCTCCATCGGGAAAATAGTTCGCACAGACTGCGGAGCTACTGATGATAA
CTATGACACTCGATTTCAAACACACTGAAAAATATCCTCACCATGTGTAATAAACCTTCT
GGCtccatttttatctttgttgACAGCGTCAAGGCGTACACACATCCCTTTCCCCCTCTC
CATACATGTGGTATTTTAGctaaagaatcccatggcctCCCAAGCACTTGTCTGGTGGGA
AGTTTGAACCCTGCAGCCCTAGCCTGGGAATATGGACTCTTGTCCCATCAACCAGAAGTT
GTGCGATGTGTTCAATAATACTCTCAAGTATACATGTTACGTGAGAAGGAAATTTTAGTT
TCGTAGTATGTGCCTAGAGATTTTGAAATGTAAACGGCTATGATATCCCAAAGGCATTTC
TGTTACTATTGTCACCAATATaacaagaaacaataaaaagtttTGGAAATGAACCATACA
CTTGAAAACTTTTGATATTCTTAACATACTGGTTCAAAACTTCTAATGAATTTATCTGAA
TAGTTTTATGaggtaaaatacacacacatttcctgcaagactccgatgctgggaaagatt
gagaacaaaaggataagagggtgacagaggatgagatggatggcatcactgactcaaagg
atgtgagtttgagcaaactctgggacatagtgaaggacagggaag
```

3.2. Dfam results from profile HMMs

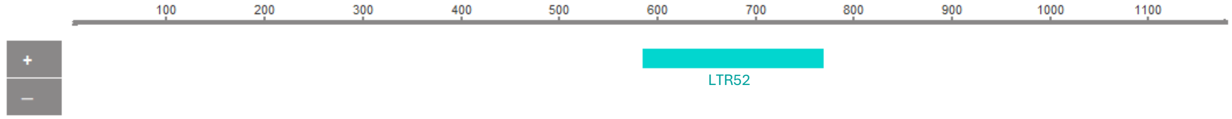

# target name	acc	query name	bits	e-value	bias	hmm-st	hmm-en	strand	ali-st	ali-en	env-st	env-en	modlen	description of target
# -------------------	-------------------	-------------------	------	---------	-----	-------	-------	------	-------	-------	-------	-------	-------	---------------------
LTR52	DF000000543.4	chr7:12934124-12935308	42.0	1.2e-11	3.9	1	192	+	586	769	586	789	421	LTR52 (Long Terminal Repeat) for ERVL endogenous retrovirus

Using *Bos taurus* as a query organism and the Dfam curated threshold (--cut_ga), only the LTR52 was detected within the query input as shown in cyan.

```
>chr7:12934124-12935308
CACTTCACGCCTTCCGGGGTTCTCCGGCGTTATTCCTTAGGCTCACGCGGCCTCTGCAGC
TCATAGCGGTAATGAAGCTGCAGCGAACTAACCTGAGCTGCTTCAACACGGCGACACTCC
ACGCAGAGCGTGCCGGGAGAGGCCGACCACACGGTTTGCGGGACGTGGCCCCACCCACCT
TCCCCTTCTGCGGTCCTGATTGGACAGTTCGCAAGCAACGCCCCCTTTATCCTGAGTAAT
AATGGATCCAGAGGCCACGTGCCTGAGCAGAATCAGCTTCTGCCGGTGGGTAGAAACCTA
CCCCTCTCCAGggacttttgtttttaaaccactCTTGTTACTTGTCCTAGAGGGACAGTG
GCAACTTCCTCAGATACTTCAGAACGTGGGGACACAGGTGTGCACAAGGAATTCCAGAGT
CAGATCCCAGAAGAGCCGACGTTCTTGTCCAAAGCAAGAGCAGGCCAACTGGAAAAACAG
GGTCTGAGGGTCCTCCATCGGGAAAATAGTTCGCACAGACTGCGGAGCTACTGATGATAA
CTATGACACTCGATTTCAAACACACTGAAAAATATCCTCACCATGTGTAATAAACCTTCT
GGCtccatttttatctttgttgACAGCGTCAAGGCGTACACACATCCCTTTCCCCCTCTC
CATACATGTGGTATTTTAGctaaagaatcccatggcctCCCAAGCACTTGTCTGGTGGGA
AGTTTGAACCCTGCAGCCCTAGCCTGGGAATATGGACTCTTGTCCCATCAACCAGAAGTT
GTGCGATGTGTTCAATAATACTCTCAAGTATACATGTTACGTGAGAAGGAAATTTTAGTT
TCGTAGTATGTGCCTAGAGATTTTGAAATGTAAACGGCTATGATATCCCAAAGGCATTTC
TGTTACTATTGTCACCAATATaacaagaaacaataaaaagtttTGGAAATGAACCATACA
CTTGAAAACTTTTGATATTCTTAACATACTGGTTCAAAACTTCTAATGAATTTATCTGAA
TAGTTTTATGaggtaaaatacacacacatttcctgcaagactccgatgctgggaaagatt
gagaacaaaaggataagagggtgacagaggatgagatggatggcatcactgactcaaagg
atgtgagtttgagcaaactctgggacatagtgaaggacagggaag
```

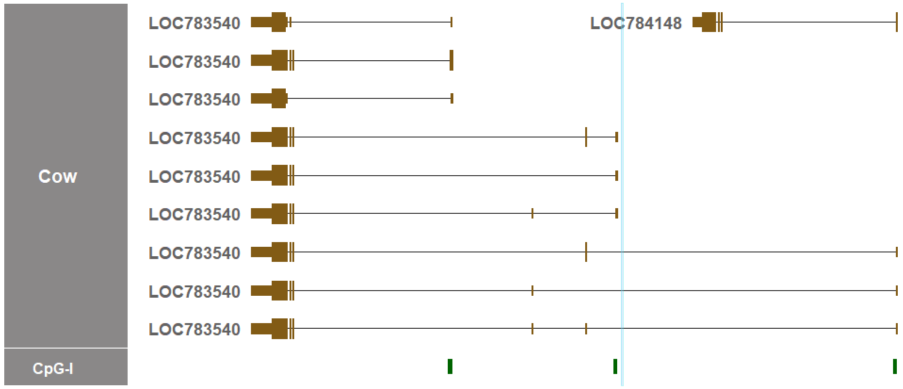

The detected region of LTR52 is indicated in cyan in both the sequence above and the genome visualizer.

4. Sheep (*Ovis aries*) sequence

4.1. Sequence search input (*ZNF791* E1 + 1kb upstream)

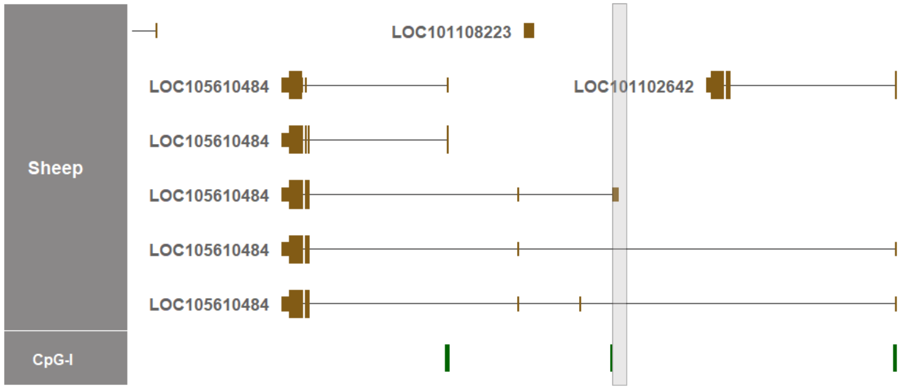

>chr5:10951059-10952708
CACTTCACACCTTCTGGGGTTCTCCAGAGACATTCCTTAGACTCACGCGGCCTCTGCAGC
TCACAGCGGTGATGAAGCTGCAGCGAACTAACCTGAGCTGCTTCAACGCTGGTGAAACTC
CACACAGAGCGTGCTAGGAGCCATCACAGGGTCTGCGGGACGTGGCCCCACCCACCTTCC
CCTGCTGCACTTCTGATTGGACAGTTTGCAAGCACAGCTCCCTTTTTCCTGAGTAATAAT
TGGTCCAGAGGCCACGTGGCTGAGCAGAATCAGCTTCTGCAGGTGGGTGGAAACCTACCC
GCCTCCAGGGACTTTTGTTTTTAAACCACTCTTGTTACTTGTCCTAGAGGGACAGTGGCA

```
ACTTCCTCAGATACTTCAGAACGTGGGGACACAGGTGTGCACAAGGAATTCCAGAGTCAG
ATTCCAGAAGAACCGACGTTCTTGTCCAAAAAAAAGAGCAGGCCAACTGGAAAAAGAGGG
TCTGAGGTGCTCCATCGGGAAAATAGTTCGCACAGATTGCGGAGCCACTGATGATACTAG
GACACTCGATTTCAAACACACTGAAAAATATCCTCACTATGTGTAATAAACCTTCTGGCT
TCATTTTTATCTTTGTTGACAGCGTCGAGGCGTACACTcatccctccccaccccccccac
cccacccTTAAGAATCCCATGGCCTCCCAAGCACTTGCCCGGTGGGAAGTTTGAACCCTG
CAGCCCTGGCCTGGGAATATGGACTCTTCTCCCATCAATCAGAACTTGTGCGACGTGTTA
AATAATATTCTCAAGTATACATGTTACGTGAGAAGGAAATTTTAGTTTCGtatgatccat
ggggtcgccaagagtcagacacaactgagcaacttcactttcacttttcactttcatgca
ttggagaaggaaatggcaacccactccagtgttcttgcctggagaatcccagggaggagg
gagcctggtgcgctgccatctatggggtcgcacagagtcagacacgactgaagcgactta
gcagcagcagcAGCAGCAGTATGTGCCTAGAGATTTTGAAATGTAAACGGCTATGATATC
CCAAAGGCAATTCAGTTACTATTGTCACCAATATAACAAGAAACAATAAAAAGTTTTGGA
AATGTACCATACACTTGAAAACTTTTGATATACTTAACATACTGGTTCAAAACTTCTAAT
GAATTTATCTGAATAGCTTTATGAGGTAAAATACACATACATTTCTTGCaagactctgat
gctgggaaagattgagcacaaaaggataagagggtgacagaggatgagatggttggatgg
cattaccgactcaaagggacatgagtttgagcaagctccaggagttggtgatggacaggg
aagcctggtgtgctgcagtcaaagaatcagacaccactgagggacGAAATAACAATAACC
ACTAAAGGGATGTTGCCTACAAATATAATATATACACAAGGGCCCATCTTTGGGAACCCT
GCCTTCACCTGTAAGGAGCATTAAACTAAAATGCCTTGTTTAGCTACAGAAAACATCCTG
ATCAGGACAAGTTAATCACTAAAGGGATGTTGCCTATAAAGCTTAAATTATATGTAATGG
CTCATCGCTGGTAACCCTGGGTTCTGTAAT
```

4.2. Dfam results from profile HMMs

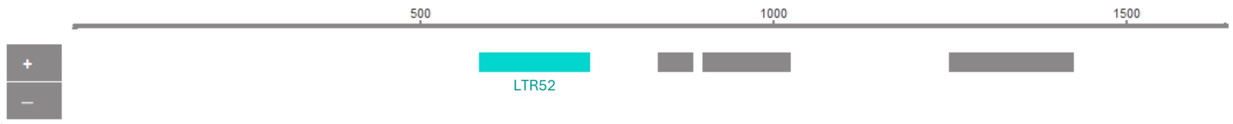

# target name	acc	query name	bits	e-value	bias	hmm-st	hmm-en	strand	ali-st	ali-en	env-st	env-en	modlen	description of target
LTR52	DF000000543.4	chr5:10951059-10952708	23.6	6.8e-06	8.9	1	175	+	582	739	582	760	421	LTR52 (Long Terminal Repeat) for ERVL endogenous retrovirus
OviAri-3.22	DF000279827.1	chr5:10951059-10952708	34.1	1.2e-08	0.0	170	219	+	836	885	816	888	221	Mammalian family identified by the Zoonomia project.
OviAri-1.57	DF000279825.1	chr5:10951059-10952708	102.3	1.4e-29	4.0	92	214	+	900	1023	895	1033	226	Mammalian family identified by the Zoonomia project.
OviAri-1.146	DF000279814.1	chr5:10951059-10952708	150.7	9.6e-46	10.8	3866	4052	+	1249	1425	1229	1431	4060	Mammalian family identified by the Zoonomia project.

Using *Ovis aries* as a query organism and the Dfam curated threshold (--cut_ga), the LTR52 was detected within the query input as shown in cyan.

```
>chr5:10951059-10952708
CACTTCACACCTTCTGGGGTTCTCCAGAGACATTCCTTAGACTCACGCGGCCTCTGCAGC
```

TCACAGCGGTGATGAAGCTGCAGCGAACTAACCTGAGCTGCTTCAACGCTGGTGAAACTC
CACACAGAGCGTGCTAGGAGCCATCACAGGGTCTGCGGGACGTGGCCCCACCCACCTTCC
CCTGCTGCACTTCTGATTGGACAGTTTGCAAGCACAGCTCCCTTTTTCCTGAGTAATAAT
TGGTCCAGAGGCCACGTGGCTGAGCAGAATCAGCTTCTGCAGGTGGGTGGAAACCTACCC
GCCTCCAGGGACTTTTGTTTTTAAACCACTCTTGTTACTTGTCCTAGAGGGACAGTGGCA
ACTTCCTCAGATACTTCAGAACGTGGGGACACAGGTGTGCACAAGGAATTCCAGAGTCAG
ATTCCAGAAGAACCGACGTTCTTGTCCAAAAAAAAGAGCAGGCCAACTGGAAAAAGAGGG
TCTGAGGTGCTCCATCGGGAAAATAGTTCGCACAGATTGCGGAGCCACTGATGATACTAG
GACACTCGATTTCAAACACACTGAAAAATATCCTCACTATGTGTAATAAACCTTCTGGCT
TCATTTTTATCTTTGTTGACAGCGTCGAGGCGTACACTcatccctccccacccccccac
cccacccTTAAGAATCCCATGGCCTCCCAAGCACTTGCCCGGTGGGAAGTTTGAACCCTG
CAGCCCTGGCCTGGGAATATGGACTCTTCTCCCATCAATCAGAACTTGTGCGACGTGTTA
AATAATATTCTCAAGTATACATGTTACGTGAGAAGGAAATTTTAGTTTCGtatgatccat
ggggtcgccaagagtcagacacaactgagcaacttcactttcacttttcactttcatgca
ttggagaaggaaatggcaacccactccagtgttcttgcctggagaatcccagggaggagg
gagcctggtgcgctgccatctatggggtcgcacagagtcagacacgactgaagcgactta
gcagcagcagcAGCAGCAGTATGTGCCTAGAGATTTTGAAATGTAAACGGCTATGATATC
CCAAAGGCAATTCAGTTACTATTGTCACCAATATAACAAGAAACAATAAAAAGTTTTGGA
AATGTACCATACACTTGAAAACTTTTGATATACTTAACATACTGGTTCAAAACTTCTAAT
GAATTTATCTGAATAGCTTTATGAGGTAAAATACACATACATTTCTTGCaagactctgat
gctgggaaagattgagcacaaaaggataagagggtgacagaggatgagatggttggatgg
cattaccgactcaaagggacatgagtttgagcaagctccaggagttggtgatggacaggg
aagcctggtgtgctgcagtcaaagaatcagacaccactgagggacGAAATAACAATAACC
ACTAAAGGGATGTTGCCTACAAATATAATATATACACAAGGGCCCATCTTTGGGAACCCT
GCCTTCACCTGTAAGGAGCATTAAACTAAAATGCCTTGTTTAGCTACAGAAAACATCCTG
ATCAGGACAAGTTAATCACTAAAGGGATGTTGCCTATAAAGCTTAAATTATATGTAATGG
CTCATCGCTGGTAACCCTGGGTTCTGTAAT

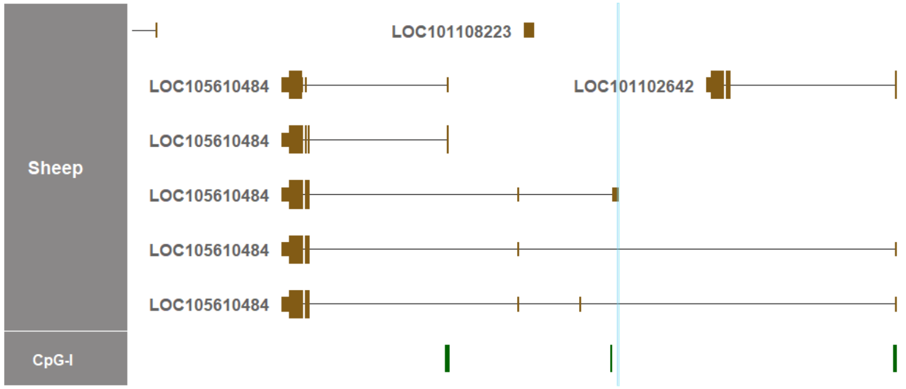

The detected region of LTR52 is indicated in cyan in both the sequence above and the genome visualizer.

5. Horse (*Equus caballus*) sequence

5.1. Sequence search input (*ZNF791* E1 + 1kb upstream)

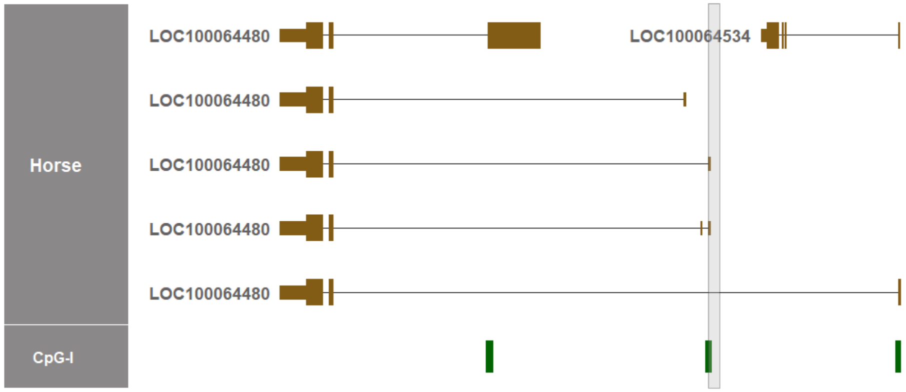


```
>chr7:47072841-47074025
CGGTTTCCCGGCTTCTGGGTACCCCGGCGTCGCCCCCACTCCGCCGGCGGCCGCGGCAGG
TCCCAGGGTACCGACGCCGCAGCAGAAACGCCGGAGCCCGTTAACTGCAGGTGAGACGCT
GTCTGAGCTCGGCCAAGAGCCTCCGCGCGGTCTGCGGGGCGTCGCCCCACCCACCTGCCC
CTGCTGCGCCTCTGATTGGACTGTTCGCAAGGGCCCGCCCCCTCGCTTCTTGAGTGACAG
TGGGGCAGGAGGTCAGACGCCTGAGCCGAATCAGCGTTCAGCGGTGAGCGGGAAACTGTG
TCGTCCGATGAGTGAGGTTTGTTTTTAAACAAAAGTTGTTCTTGTCCCTAAAGGGACAGT
GGCGTCTTCCTGCAGCGCCTTCTGAACGCGGAGACACAGGTGTGCAGGGAATTCCAGAGT
CAGTTGCCAGATGGAGCGATGCCCTTATCCAGAGCAAGAGGGCGCCCTAGCCAGGCTAGA
CAGGGACCAGAGGGTCTGAGGTccccgcccctccccccctcccccccccagcccccGGGTC
AGGGGTTGGCATAAGGCTGGGAAGCCACTCATGGAACCGTGACTCATTTACAAACACGCT
TAAATATGACATCACCACATGTAAGGGAGAGTCTGGCTCCATTTTGACCTTTGTAGACAG
CGCTGAGGCGTCCCCCCATCCCTTCCCCACCTCCGCACGTTTGGTACTTTACATAAAGAA
TCCCATGGCCTCCCAAGGGTCTGGCTCTGGGGGGATCTGAACCCTGCAGCCCTCGCCTGT
GCGCAGAAACTCGTCTCTGACCAGCTGTGAGCTTTGAGATGGGTTCAATAAAACTCAGGT
GTACATTTTACACTAGAAGGAAATTTATTTTACTTTCTGAGCATGACCTTAAGAGATTTT
GAAATGTAAACAGCTATGATATCCCTAAGGCAACTGAATTCTGATTTCCACCAATATACA
TATAAAAACAATGTTATGTAAATGTGAGCTGTACCTGAAAACTCTTTAAGATACTTAACA
CATTGGTTCAAAAAATTCCTAATTCATTTAtttgaatagctttactgagatataatacac
atattatacaaccctttaatgtgtataattcaatggttttcagtacattccgatatgtgc
```

aataatcatcacaacttttgaacatttttgtggcttcaaaaagaa

5.2. Dfam results from profile HMMs

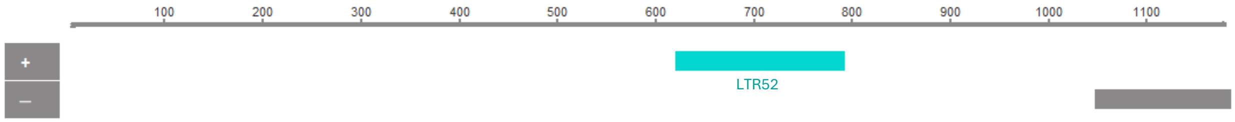

# target name	acc	query name	bits	e-value	bias	hmm-st	hmm-en	strand	ali-st	ali-en	env-st	env-en	modlen	description of target
# -------------------	-------------------	-------------------	------	---------	-----	-------	-------	------	-------	-------	-------	-------	-------	---------------------
LTR52	DF000000543.4	chr7:47072841-47074025	24.5	2.4e-06	5.1	1	176	+	620	792	620	808	421	LTR52 (Long Terminal Repeat) for ERVL endogenous retrovirus
L1MB8_3end	DF000000274.4	chr7:47072841-47074025	68.4	4e-20	18.3	779	923	-	1185	1051	1185	1047	925	3' end of L1 retrotransposon, L1MB8_3end subfamily

Using *Equus caballus* as a query organism and the Dfam curated threshold (--cut_ga), the LTR52 was detected within the query input as shown in cyan.

```
>chr7:47072841-47074025
CGGTTTCCCGGCTTCTGGGTACCCCGGCGTCGCCCCCACTCCGCCGGCGGCCGCGGCAGG
TCCCAGGGTACCGACGCCGCAGCAGAAACGCCGGAGCCCGTTAACTGCAGGTGAGACGCT
GTCTGAGCTCGGCCAAGAGCCTCCGCGCGGTCTGCGGGGCGTCGCCCCACCCACCTGCCC
CTGCTGCGCCTCTGATTGGACTGTTCGCAAGGGCCCGCCCCCTCGCTTCTTGAGTGACAG
TGGGGCAGGAGGTCAGACGCCTGAGCCGAATCAGCGTTCAGCGGTGAGCGGGAAACTGTG
TCGTCCGATGAGTGAGGTTTGTTTTTAAACAAAAGTTGTTCTTGTCCCTAAAGGGACAGT
GGCGTCTTCCTGCAGCGCCTTCTGAACGCGGAGACACAGGTGTGCAGGGAATTCCAGAGT
CAGTTGCCAGATGGAGCGATGCCCTTATCCAGAGCAAGAGGGCGCCCTAGCCAGGCTAGA
CAGGGACCAGAGGGTCTGAGGTccccgcccctccccccctccccccccagcccccGGGTC
AGGGGTTGGCATAAGGCTGGGAAGCCACTCATGGAACCGTGACTCATTTACAAACACGCT
TAAATATGACATCACCACATGTAAGGGAGAGTCTGGCTCCATTTTGACCTTTGTAGACAG
CGCTGAGGCGTCCCCCCATCCCTTCCCCACCTCCGCACGTTTGGTACTTTACATAAAGAA
TCCCATGGCCTCCCAAGGGTCTGGCTCTGGGGGGATCTGAACCCTGCAGCCCTCGCCTGT
GCGCAGAAACTCGTCTCTGACCAGCTGTGAGCTTTGAGATGGGTTCAATAAAACTCAGGT
GTACATTTTACACTAGAAGGAAATTTATTTTACTTTCTGAGCATGACCTTAAGAGATTTT
GAAATGTAAACAGCTATGATATCCCTAAGGCAACTGAATTCTGATTTCCACCAATATACA
TATAAAAACAATGTTATGTAAATGTGAGCTGTACCTGAAAACTCTTTAAGATACTTAACA
CATTGGTTCAAAAAATTCCTAATTCATTTAtttgaatagctttactgagatataatacac
atattatacaaccctttaatgtgtataattcaatggttttcagtacattccgatatgtgc
aataatcatcacaacttttgaacatttttgtggcttcaaaaagaa
```

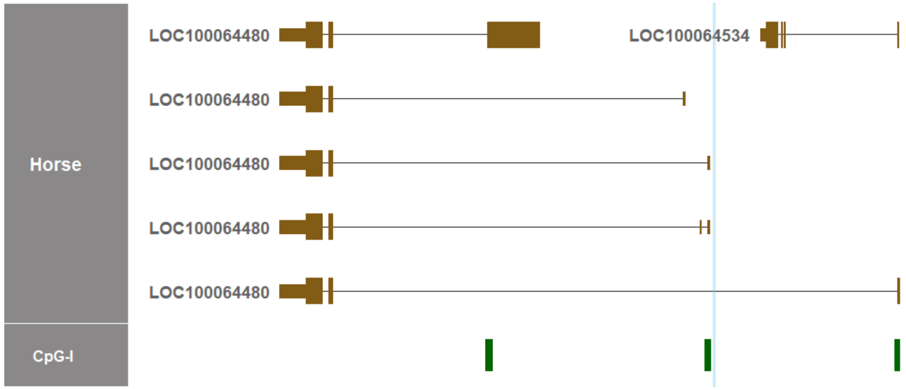

The detected region of LTR52 is indicated in cyan in both the sequence above and the genome visualizer.

6. Goat (*Capra hircus*) sequence

6.1. Sequence search input (*ZNF791* E1 + 1kb upstream)

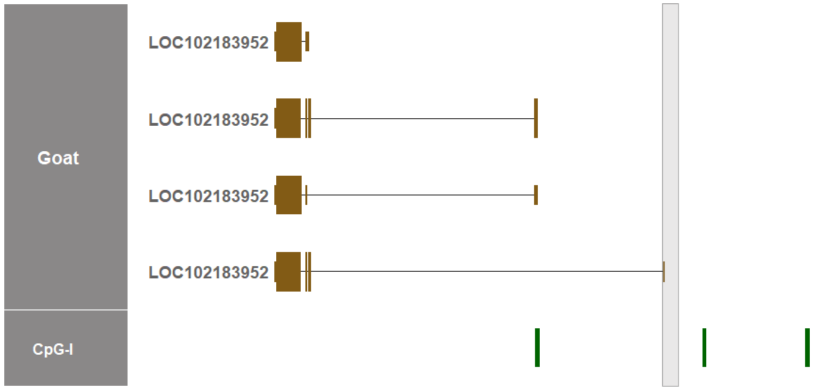


```
>chr7:9974100-9975196
CCTGTGCTCTTGGAGCCAAGAGGATGCAGCTCACATCCTGACTGCAGAGACCGTGTGCAG
GGGCAGCGGTGCTGAGGCTCCCAAAGCTAACCAGGTCAAGGTGTATCACGTCCCTTCAGA
GGGGAGGGCCAAGTGCAGCAAAGAAGCTGTTGAAATAGGCATCAAACACAACAAATTTAT
CCGAATATGTGAGAGCAGGGTATCTGCCAGCTGTTGACGGGATGGAGTCGGTAATTTCCA
CAATGTTGTGGACTGAGTACAGAGGTTCATTTGGTGTGACCACAACCTCAAGTGTTCAGT
```

```
AATCCACTGTCAGGTGTCATTCAGTCTTTTTAGATTTAAGAGCAGGCCAAattcagctgt
gaaaagaagtacAGGGAGTAATCACCTCTTTGCTAAATAATTTTTGtggggacttctttg
gtggcccagtggttaagaatctgcgtttccacttcagggggcacaggttcaatccctggg
tgaggaactAACATCCTAGGTGCTAtatggtgcggccaaaaaaatttttttttggtaat
ggtCTTCAATCCATCAAGGCCCTGTTTAATTTTCATTGGCCCATATTATGTAATTTAGAC
TTCCTCAAtggctcagtggggaaagaatctccctgcaaagGAGGAGAtaaaggagactca
ggtttgatccctgggttgggaagatcccctgcaaaaagaaatggcaacgcactctagtat
tcttgcctgaaaaatcccgtggacagaagagcctggcaggctacagtccatggggtcaca
aagagtcggatacaactgagcatgagcacaatAATGCAATTTACCTAGGGGGGTAGACTG
TGGGGTCCTATTTTGTCAAGCCAATTTGGAATTAGCAAAAAGTATTTAACTTTAATTTGT
GAATCATTGGTCCAGAGCTTTCCTGTTCACTATAGAAAACTTTAGGAAAATTGATCCTAT
GACCGTGGGAGAATTTAGGCAAGAAAAGAGTTGGCTGACAATCATTAAAGAAAGATATGC
CTATTCAGTAATTCCTCTAAGATTATGGGAAGTTCCTTGCTTAAGTTTGttaatgttgtt
tagtcgctcagttgtgt
```

6.2. Dfam results from profile HMMs

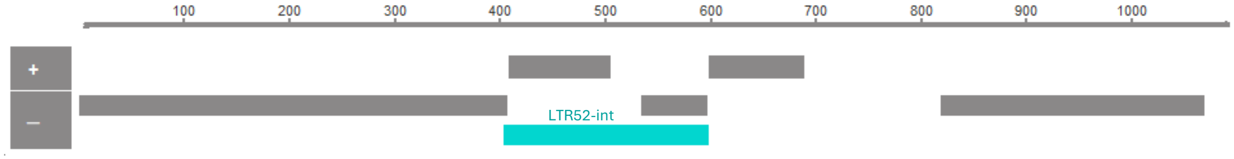

# target name	acc	query name	bits	e-value	bias	hmm-st	hmm-en	strand	ali-st	ali-en	env-st	env-en	modlen	description of target
# -------------------	-------------------	-------------------	------	---------	-----	-------	-------	------	-------	-------	-------	-------	-------	---------------------
GirTip-1.94	DF000277154.1	chr7:9974100-9975196	57.2	6.6e-16	0.9	1	95	+	409	504	409	524	173	Mammalian family identified by the Zoonomia project.
AmmLer-1.137	DF000274749.1	chr7:9974100-9975196	40.5	4.5e-11	0.0	3	84	+	599	689	597	709	317	Mammalian family identified by the Zoonomia project.
LTR53-int	DF000000547.5	chr7:9974100-9975196	220.6	4.9e-67	3.4	1194	1603	-	406	1	419	1	3329	Internal region of ERVL endogenous retrovirus, LTR53-int subfamily
LTR52-int	DF000000544.5	chr7:9974100-9975196	14.4	7e-05	7.0	1481	1698	-	598	404	620	397	3556	Non-autonomous Internal region of HERV52, LTR52-int subfamily
LTR53-int	DF000000547.5	chr7:9974100-9975196	16.9	1.2e-05	7.3	1131	1194	-	596	534	618	516	3329	Internal region of ERVL endogenous retrovirus, LTR53-int subfamily
LTR53-int	DF000000547.5	chr7:9974100-9975196	114.2	5.7e-35	16.0	876	1134	-	1068	819	1076	799	3329	Internal region of ERVL endogenous retrovirus, LTR53-int subfamily

Using *Capra hircus* as a query organism and the Dfam curated threshold (--cut_ga), the LTR52-int was detected within the query input as shown in cyan.

```
>chr7:9974100-9975196
CCTGTGCTCTTGGAGCCAAGAGGATGCAGCTCACATCCTGACTGCAGAGACCGTGTGCAG
GGGCAGCGGTGCTGAGGCTCCCAAAGCTAACCAGGTCAAGGTGTATCACGTCCCTTCAGA
GGGGAGGGCCAAGTGCAGCAAAGAAGCTGTTGAAATAGGCATCAAACACAACAAATTTAT
CCGAATATGTGAGAGCAGGGTATCTGCCAGCTGTTGACGGGATGGAGTCGGTAATTTCCA
CAATGTTGTGGACTGAGTACAGAGGTTCATTTGGTGTGACCACAACCTCAAGTGTTCAGT
AATCCACTGTCAGGTGTCATTCAGTCTTTTTAGATTTAAGAGCAGGCCAAattcagctgt
gaaaagaagtacAGGGAGTAATCACCTCTTTGCTAAATAATTTTTGtggggacttctttg
```

gtggcccagtggttaagaatctgcgtttccacttcagggggcacaggttcaatccctggg
tgaggaactAACATCCTAGGTGCTAatatggtgcggccaaaaaaatttttttttggtaat
ggtCTTCAATCCATCAAGGCCCTGTTTAATTTTCATTGGCCCATATTATGTAATTTAGAC
TTCCTCAAtggctcagtggggaaagaatctccctgcaaagGAGGAGAtaaaggagactca
ggtttgatccctgggttgggaagatcccctgcaaaaagaaatggcaacgcactctagtat
tcttgcctgaaaaatcccgtggacagaagagcctggcaggctacagtccatggggtcaca
aagagtcggatacaactgagcatgagcacaatAATGCAATTTACCTAGGGGGGTAGACTG
TGGGGTCCTATTTTGTCAAGCCAATTTGGAATTAGCAAAAAGTATTTAACTTTAATTTGT
GAATCATTGGTCCAGAGCTTTCCTGTTCACTATAGAAAACTTTAGGAAAATTGATCCTAT
GACCGTGGGAGAATTTAGGCAAGAAAAGAGTTGGCTGACAATCATTAAAGAAAGATATGC
CTATTCAGTAATTCCTCTAAGATTATGGGAAGTTCCTTGCTTAAGTTTGttaatgttgtt
tagtcgctcagttgtgt

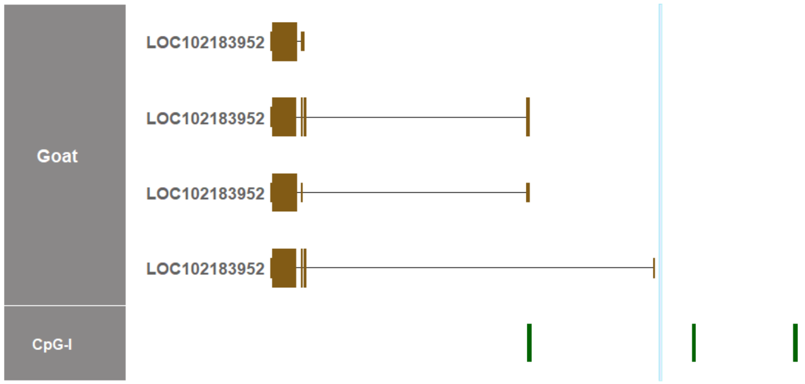

The detected region of LTR52-int is indicated in cyan in both the sequence above and the genome visualizer.

7. Dog (*Canis lupus familiaris*) sequence

7.1. Sequence search input (*ZNF791* E1 + 1kb upstream)

>chr20:49516920-49518052
CTCGAAGGGCCTCATCATCCCTTTGTGCATATGCATCCCCAGCGTTCCAGGCTCTGTAGC
TTCTCTTCATACAATGGCTCCTTCTGTGCTGGGTATGAGCAGGAAGGACTTCTGCAGAGG
CTCCTCAGGAAGAACCagctgggttctttttttttttttttttttaaagattttattta
tttacatatgagagaccagagagagagaggcagagacacaggcagagggagaagcaggct
ccatgcagggagcccgggactccaggaccacaccctgggctgaaggcaggtgctaaaccg
ctgagccacccgggctgcccccccccctttttttttaaagatttatttattcttgagagac
agagaaagagagagagagagagagagagaggcagagggagaagcaggctccacgcaggga

```
gcccgatatgggactggatcctgggactccaggatcatgccctgggccgaaggcaagcgc
tcaaccgctgagccacccagggatccctgaaccaGCTAGGTCTTGAAGGTATTTCCCTAT
GCAGGCTCAATGAGAAACGGGGTGGGGAGCCTACCTTGGGTCTAGTCATAGTCTTCTGCT
CCTAGTCCCATAGCTTTGGactatattgatatatttaatgACACAAACCCAGTATTAAAT
AATCTAGAAAAACCGCACAAATCCAATGTTTATGTagaaaagatggaagaagattgtaag
gctttttttttccagctcaacAAGAAAATCCACAAGTATTTATTACTTTACGAAACAAT
GAGAGTAGATTATTATTTCCATGGCAAGAAGCTGTCCTATAAACAAACAtacttgaatat
tattttttgaagattttatttatttatttatgagagaccaagagagagacagagaggcag
agacacacaggcagagagggaagcaggctccatacagggagccggatgtgggactcaatc
ccgggtctccaggatcactccatgagctgaaggtggcgctaaaccgctgagccacccagg
ctagtACTTGAATATTCTAATTGCTGGAATTATATTGGAAACTGCTCCCCTTCGCcctca
cattaaaaaacaaaacaatacagacCTTAGAATATTATTACACCTTCTCAGGg
```

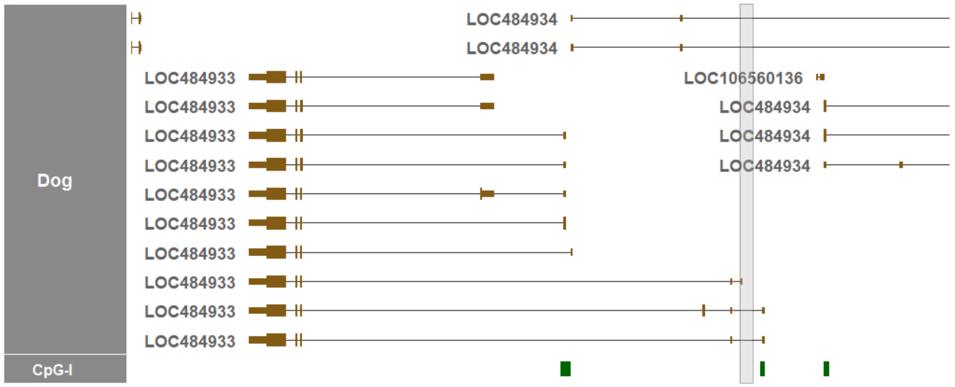

7.2. Dfam results from profile HMMs

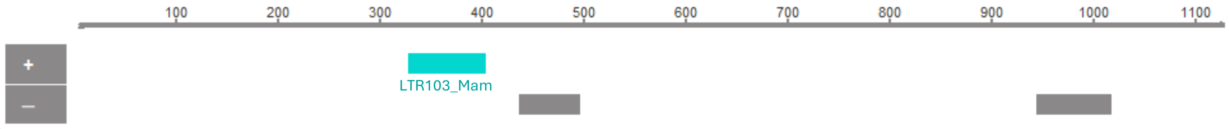

# target name	acc	query name	bits	e-value	bias	hmm-st	hmm-en	strand	ali-st	ali-en	env-st	env-en	modlen	description of target
# -------------------	-------------------	-------------------	------	---------	-----	-------	-------	------	-------	-------	-------	-------	-------	---------------------
LTR103_Mam	DF000000379.4	chr20:49516920-49518052	9.8	0.051	4.9	126	205	+	328	404	315	425	475	LTR103_Mam (Long Terminal Repeat) likely for ERV1 endogenous retrovirus
tRNA-Ala-GCG	DF000000637.4	chr20:49516920-49518052	12.5	0.092	3.1	7	55	-	496	437	508	420	72	tRNA-Ala (decoding GCG) gene / pseudogene
tRNA-Leu-CTG	DF000000678.4	chr20:49516920-49518052	13.0	0.075	2.9	5	78	-	1017	945	1021	940	83	tRNA-Leu (decoding CTG) gene / pseudogene

Using *Canis lupus familiaris* as a query organism and the E-value threshold (-E 0.1), the LTR103-Mam was detected within the query input as shown in cyan.

```
>chr20:49516920-49518052
CTCGAAGGGCCTCATCATCCCTTTGTGCATATGCATCCCCAGCGTTCCAGGCTCTGTAGC
TTCTCTTCATACAATGGCTCCTTCTGTGCTGGGTATGAGCAGGAAGGACTTCTGCAGAGG
CTCCTCAGGAAGAACCagctgggttctttttttttttttttttttttaaagattttattta
tttacatatgagagaccagagagagagaggcagagacacaggcagagggagaagcaggct
ccatgcagggagcccgggactccaggaccacaccctgggctgaaggcaggtgctaaaccg
ctgagccacccgggctgccccccccccttttttttaaagatttatttattcttgagagac
agagaaagagagagagagagagagagagagaggcagagggagaagcaggctccacgcaggga
gcccgatatgggactggatcctgggactccaggatcatgccctgggccgaaggcaagcgc
tcaaccgctgagccacccagggatccctgaaccaGCTAGGTCTTGAAGGTATTTCCCTAT
GCAGGCTCAATGAGAAACGGGGTGGGGAGCCTACCTTGGGTCTAGTCATAGTCTTCTGCT
CCTAGTCCCATAGCTTTGGactatattgatatatttaatgACACAAACCCAGTATTAAAT
AATCTAGAAAAACCGCACAAATCCAATGTTTATGTagaaaagatggaagaagattgtaag
gcttttttttttccagctcaacAAGAAAATCCACAAGTATTTATTACTTTACGAAACAAT
GAGAGTAGATTATTATTTCCATGGCAAGAAGCTGTCCTATAAACAAACAtacttgaatat
tatttttttgaagatttatttatttatttatgagagaccaagagagagacagagaggcag
agacacacaggcagagagggaagcaggctccatacagggagccggatgtgggactcaatc
ccgggtctccaggatcactccatgagctgaaggtggcgctaaaccgctgagccacccagg
ctagtACTTGAATATTCTAATTGCTGGAATTATATTGGAAACTGCTCCCCTTCGCcctca
cattaaaaaacaaaacaatacagacCTTAGAATATTATTACACCTTCTCAGGg
```

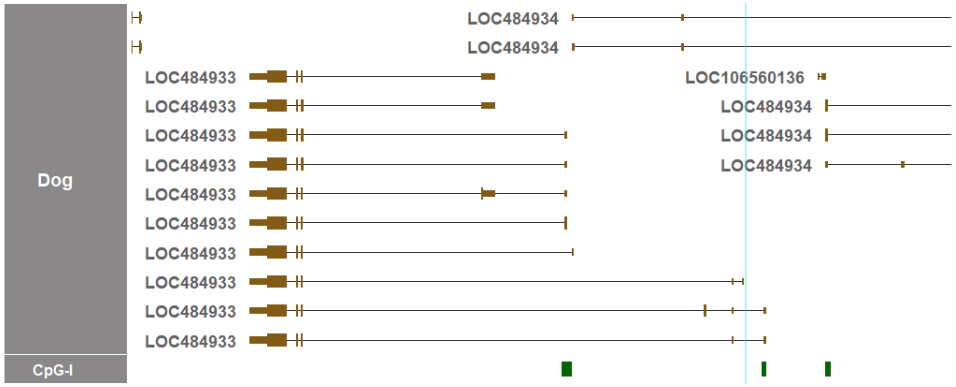

The detected region of LTR103-Mam is indicated in cyan in both the sequence above and the genome visualizer.

8. Summary of gene and LTR orientations

Species	Gene symbol	Gene description	Gene orientation	LTR	LTR orientation	LTR position relative to gene
Pig	LOC100521431	ZNF791-like	–	LTR52	+	5'
Cattle	LOC783540	ZNF791	–	LTR52	+	5'
Sheep	LOC105610484	ZNF791-like	–	LTR52	+	5'
Horse	LOC100064480	ZNF791	–	LTR52	+	5'
Goat	LOC102183952	ZNF791	–	LTR52-int	–	5'
Dog	LOC484933	ZNF791	–	LTR103_Mam	+	5'

9. Comparison of gene and LTR annotations in NCBI, UCSC, Dfam, and Ensembl for pigs

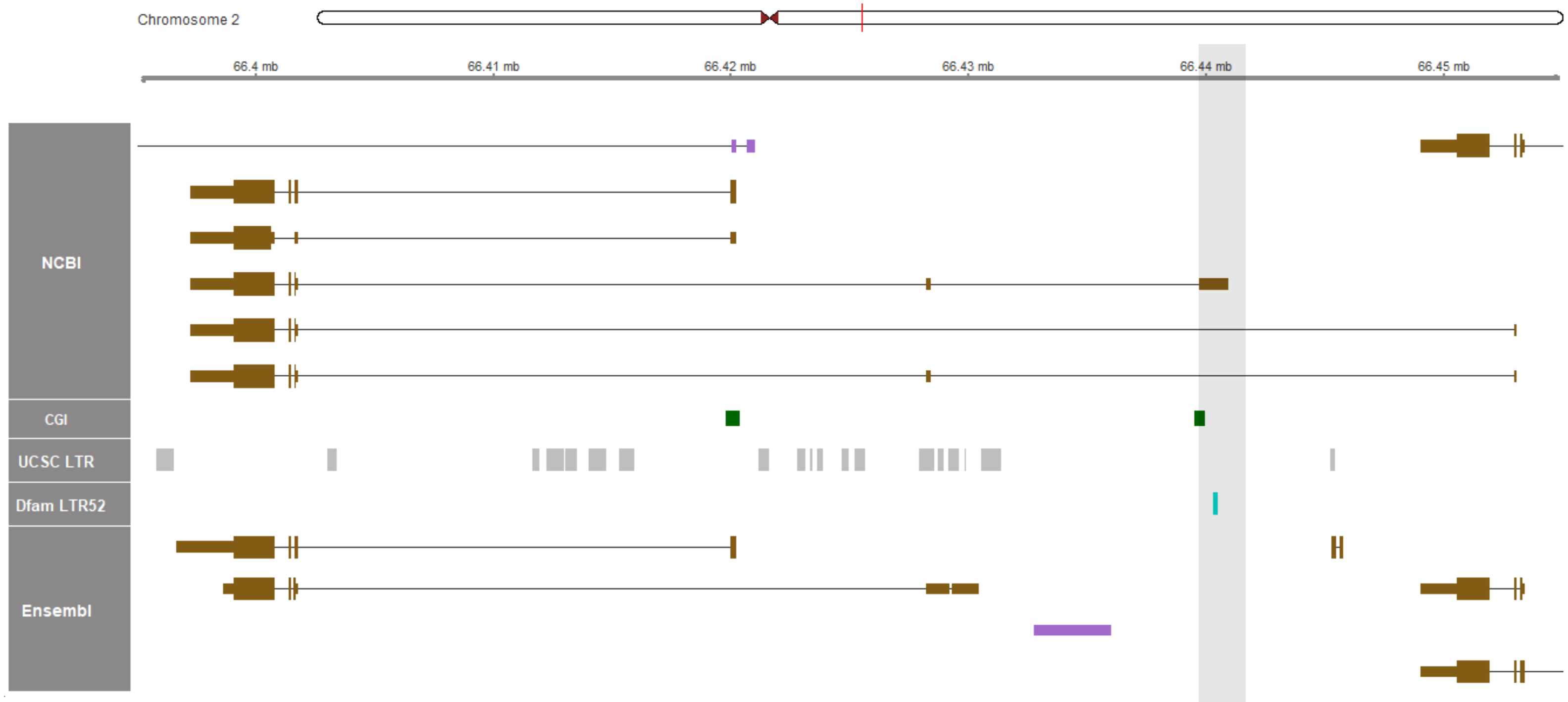

The region of interest is highlighted in grey, while the location of LTR52 in pigs is highlighted in cyan (as shown in 2. Pig (*Sus scrofa*) sequence). The Dfam search was based on the recent v3.8 annotation for the susScr11 pig genome assembly (latest update on November 16, 2023; https://www.dfam.org/releases/Dfam_3.8/annotations/DAsusScro11.1/). The UCSC LTR annotation is based on the January 31,2015 version of RepeatMasker with the Repbase library release 20140131 for the susScr11 assembly (https://hgdownload.soe.ucsc.edu/goldenPath/susScr11/bigZips/). The Ensembl annotation for LTR appears to be similar to UCSC's, while the target transcript is absent in Ensemble gene annotation.
