# Supplementary material for: Evolutionary lineage-specific genomic imprinting at the ZNF791 locus
Source: PLoS Genet. 2025 Jan 15;21(1):e1011532. doi: 10.1371/journal.pgen.1011532 (PMC11734915; doi:10.1371/journal.pgen.1011532)
Supplement: S17 Fig — (PDF) [file pgen.1011532.s017.pdf]

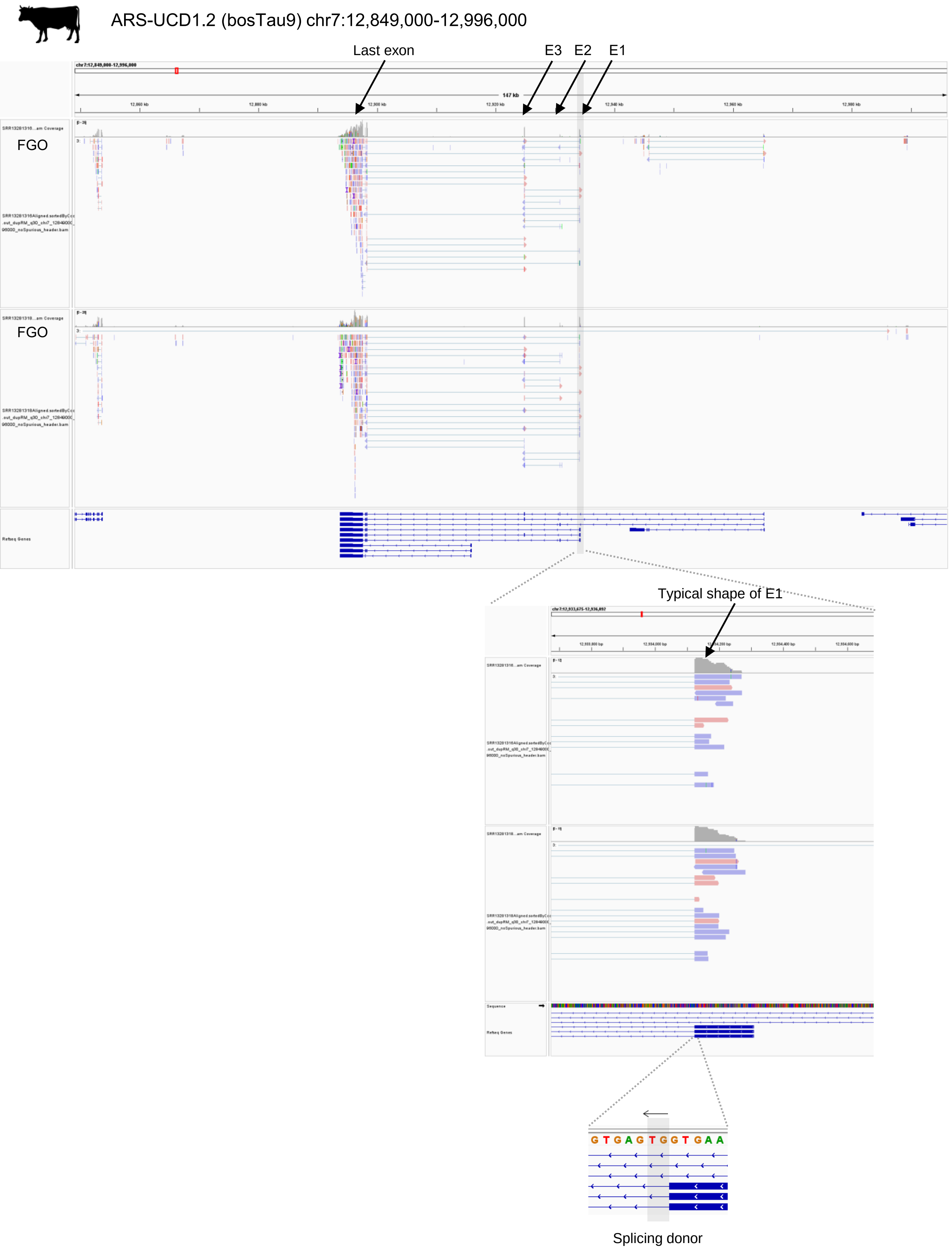

S17 Fig. Expressed transcripts at the *ZNF791* locus in cow oocytes. Detailed RNA-seq read coverages in full grown oocytes (FGOs) from cows (the same data used in Fig 5) is shown using integrative genome viewer (IGV).
