# Supplementary material for: Evolutionary lineage-specific genomic imprinting at the ZNF791 locus
Source: PLoS Genet. 2025 Jan 15;21(1):e1011532. doi: 10.1371/journal.pgen.1011532 (PMC11734915; doi:10.1371/journal.pgen.1011532)
Supplement: S18 Fig — (PDF) [file pgen.1011532.s018.pdf]

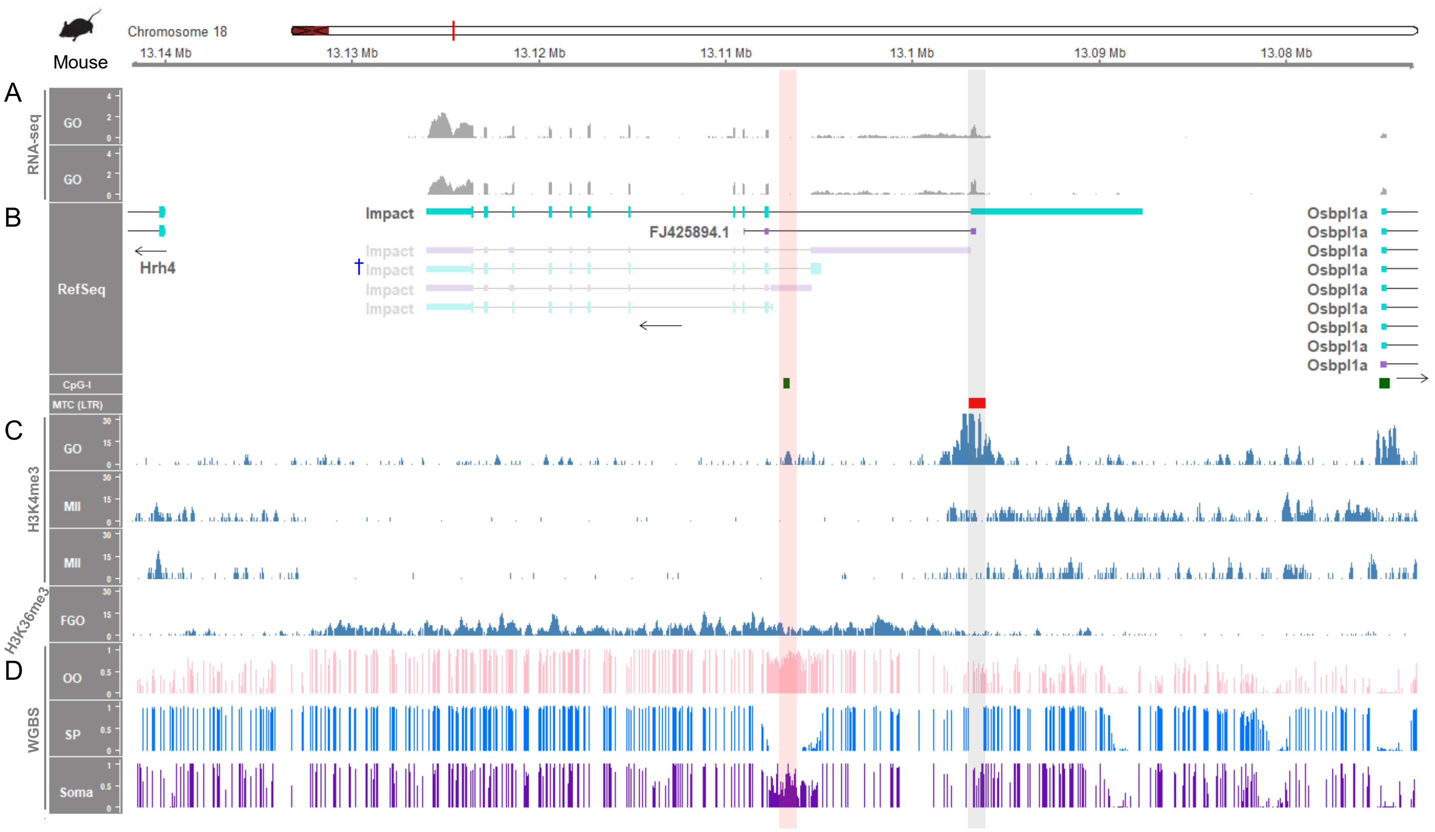

S18 Fig. LTR-initiated transcription and establishment of methylation imprint in mouse oocytes. This mechanism of imprinting was previously described for the known imprinted gene, mouse *Impact* (Bogutz, et al. 2019). **(A, B)** RNA-seq read coverages from growing oocytes (GOs) and the expressed *Impact* transcript. Besides the NCBI RefSeq annotation, the alternative short 1st exon was supported by an EST sequence (FJ425894.1) from the UCSC genome browser database. The LTR element (MTC) located around the TSS are marked with the red rectangle and highlighted with grey shading. Non-expressed transcripts are shown as faded. **(C)** Histone modifications, H3K4me3 and H3K36me3, in oocytes. **(D)** WGBS methylation ratios of mouse samples. Full methylation in oocytes (OO), unmethylation in sperm (SP), and partial methylation in somatic tissue (kidney) are highlighted with red shading. From the red-shaded region surrounding the intron 1 CpG island (CpG-I), the *Impact* transcript, indicated by a † in (B), is transcribed at later stages as an imprinted transcript. In addition, the rat *Impact* gene is also imprinted, but the *IMPACT* gene in humans and pigs are not imprinted (geneimprint.com).
