# Supplementary material for: Evolutionary lineage-specific genomic imprinting at the ZNF791 locus
Source: PLoS Genet. 2025 Jan 15;21(1):e1011532. doi: 10.1371/journal.pgen.1011532 (PMC11734915; doi:10.1371/journal.pgen.1011532)
Supplement: S19 Fig — (PDF) [file pgen.1011532.s019.pdf]

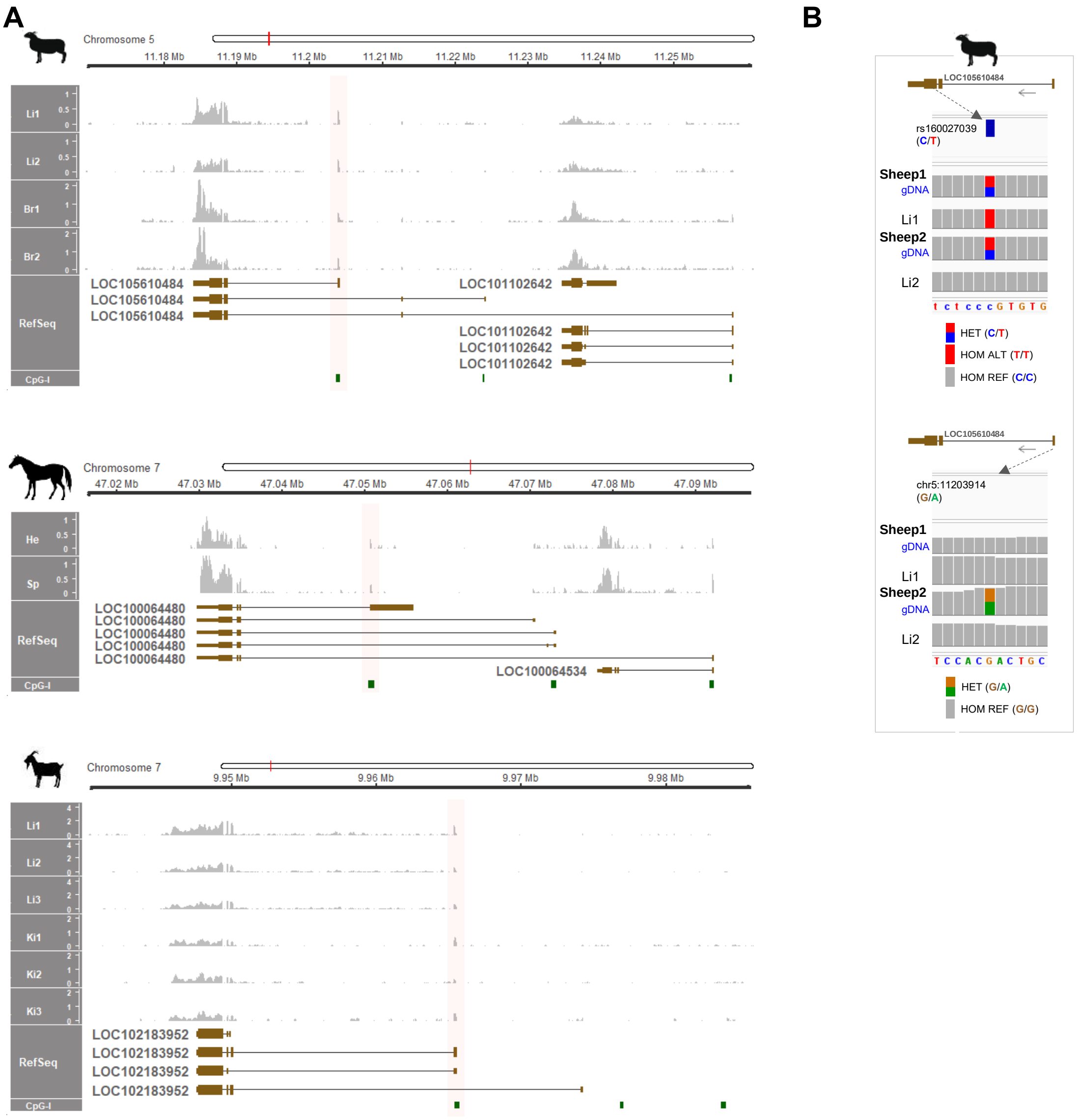

S19 Fig. Expressed *ZNF791* transcripts in sheep, horses, and goats. (A) Highlights in red are on the 1st exon of expressed transcripts from sheep, horses, and goats. For horses, reads were aligned to the equCab3 reference genome, displaying the short 1st exon as annotated in the equCab2 genome. RNA-seq data for sheep liver were derived from the GEO database under accession number PRJEB19199. RNA-seq data from brain (hypothalamus) of approximately 4-month-old sheep are from our published data (GSE253249). Adult horse heart (He) and spleen (Sp) RNA-seq data are from a dataset under accession number PRJEB26787. RNA-seq data for 3-year-old goats (Li, liver; Ki, kidney) are from a dataset under accession number GSE77020. **(B)** Both sheep liver RNA-seq and WGS data are from the same individuals under accession number PRJEB19199. Informative SNPs were as follows: sheep1 gDNA (C:T=5 reads: 6 reads; 45%:55%) and liver mRNA (C:T=0 read /12 reads, 0%:100%), Sheep2 gDNA (C:T=6 reads: 8 reads, 43%:57%) and liver mRNA (C:T=14 reads: 0 read, 100%:0%), Sheep2 gDNA (G:A=7 reads: 7 reads, 50%:50%) and liver mRNA (G:A=16 reads: 1 read, 94%:6%).
