# Supplementary material for: Evolutionary lineage-specific genomic imprinting at the ZNF791 locus
Source: PLoS Genet. 2025 Jan 15;21(1):e1011532. doi: 10.1371/journal.pgen.1011532 (PMC11734915; doi:10.1371/journal.pgen.1011532)
Supplement: S20 Fig — (PDF) [file pgen.1011532.s020.pdf]

```
                                10        20        30        40        50        60        70        80        90       100
                       ....|....|....|....|....|....|....|....|....|....|....|....|....|....|....|....|....|....|....|....|
LTR52_consensus      1 --TGTAATAAAGAGTCTGACTCCATTTTTTGATGTTTGACTGCTGA--CAGCTTTTAAGCCT-CACCCCTCCCTCTTCCCTTTGCCCCACATCTGGGCAA 95
pig_LTR52            1 --TGTAATGAAGAGTCTAGCTCCATTTCA--GTCTT----TGTTGA--CAGGGTTGAGATGTCCACACATCTGCTTTTCCCCTTCCATACACGAGGT--A 88
cow_LTR52            1 --TGTAATAAACCTTCTGGCTCCATTTTT--ATCTT----TGTTGA--CAGCGTCAAGGCGTACACACATCCCTTTCCCCCTCTCCATACATGTGGT--A 88
sheep_LTR52          1 --TGTAATAAACCTTCTGGCTTCATTTTT--ATCTT----TGTTGA--CAGCGTCGAGGCGTACACTCATCCCTCCCCACCCCCCCC------------A 78
horse_LTR52          1 --TGTAAGGGAGAGTCTGGCTCCATTTTG--ACCTT----TGTAGA--CAGCGCTGAGGCGTCCCCCCATCCCT-TCCCCACCTCCGCACGTTTGGT--A 87
goat_LTR52-int       1 ----TTGTGGGGACT-----------------TCTT----TGGTGGCCCAGTGGTTAAGAATC------TGCGTTTCCACTTCAGGGGGCACAGGTTCAA 69
dog_LTR103_Mam       1 TTTTTTTTAAAGA-------TTTATTTAT---TCTT----GAGAGA--CAGAG----------------------------------------------- 37

                               110       120       130       140       150       160       170       180       190       200
                       ....|....|....|....|....|....|....|....|....|....|....|....|....|....|....|....|....|....|....|....|
LTR52_consensus     96 GCTGA---TAAGAAAGCCCGGGTGCTCCC--TCCTT---TGGCGCCGGCGGGAAG---------------------TTCAAACCACGCAAGCCCCTGCCT 166
pig_LTR52           89 TTTAA-GTT----AATCTCCTGGCCTCCCAAGCACT---TGACTCTGCTGAGAAA---------------------TTTGAGCCGTGC-AGCCCTCGCT- 157
cow_LTR52           89 TTTTA-GCTAAAGAATCCCATGGCCTCCCAAGCACT---TG--TCTGGTGGGAAG---------------------TTTGAACCCTGC-AGCCCTAGCC- 159
sheep_LTR52         79 CCCCACCCTTAAGAATCCCATGGCCTCCCAAGCACT---TG--CCCGGTGGGAAG---------------------TTTGAACCCTGC-AGCCCTGGCC- 150
horse_LTR52         88 CTTTA-CATAAAGAATCCCATGGCCTCCCAAGGGTC---TGGCTCTGGGGGGA-----------------------TCTGAACCCTGC-AGCCCTCGCC- 158
goat_LTR52-int      70 TCCCTGGGTGAGGAAC----TAACATCCTAGGTGCTATATGGTGCGGCCAAAAAAATTTTTTTTTTGGTAATGGTCTTCAATCCATCAAGGCCCTGTTT- 164
dog_LTR103_Mam      37 ---------AAAGAGA------------------------------GAGAGAGAG---------------------AGAGAGAGAGGC-AG--------- 67

                               210       220       230       240       250       260       270       280       290       300
                       ....|....|....|....|....|....|....|....|....|....|....|....|....|....|....|....|....|....|....|....|
LTR52_consensus    167 GCGGGAACCCTCACCCCGGCCCCACCCCCNTAACCACAATAAAAACCCCAAGCCAGTCTCCTTTCCCTGCTCTCTCAAGCCATTTTCGGACCTGCTTGGG 266
pig_LTR52          157 ---------------------------------------------------------------------------------------------------- 157
cow_LTR52          159 ---------------------------------------------------------------------------------------------------- 159
sheep_LTR52        150 ---------------------------------------------------------------------------------------------------- 150
horse_LTR52        158 ---------------------------------------------------------------------------------------------------- 158
goat_LTR52-int     164 ---------------------------------------------------------------------------------------------------- 164
dog_LTR103_Mam      67 ---------------------------------------------------------------------------------------------------- 67

                               310       320       330       340       350       360       370       380       390       400
                       ....|....|....|....|....|....|....|....|....|....|....|....|....|....|....|....|....|....|....|....|
LTR52_consensus    267 AGGCCTGCCCTGCTCTCCCCAGAAAGCCTCANTTATGTGAGTAATAAACCTTTTCATACCCTCTTGGTGCGTGTGTGGCATCATCAGTCTCGACATCCGA 366
pig_LTR52          157 -----------------------------------TGGGAACAGGGACTCTTCTACCAGCCG-------------------------------------- 184
cow_LTR52          159 -----------------------------------TGGGAATATGGACTCTTGTCCCATC---------------------------------------- 184
sheep_LTR52        150 -----------------------------------TGGGAATA--------------------------------------------------------- 158
horse_LTR52        158 -----------------------------------TGTGCGCAGAAACTC-------------------------------------------------- 173
goat_LTR52-int     164 -----------------------------------AATTTTCATTGGCCCATATTATGTAATTTAG---------------------------------- 195
dog_LTR103_Mam      67 -----------------------------------AGGGAGAAGC------------------------------------------------------- 77

                               410       420       430       440       450
                       ....|....|....|....|....|....|....|....|....|....|....|
LTR52_consensus    367 ACCAAATTTTGGGTGGGGGAGTCCATCCTGCCTCTGCAGGGTGACCACAACAACA 421
pig_LTR52          184 ------------------------------------------------------- 184
cow_LTR52          184 ------------------------------------------------------- 184
sheep_LTR52        158 ------------------------------------------------------- 158
horse_LTR52        173 ------------------------------------------------------- 173
goat_LTR52-int     195 ------------------------------------------------------- 195
dog_LTR103_Mam      77 ------------------------------------------------------- 77
```

S20 Fig. Multiple sequence alignment of LTRs. The consensus LTR52 sequence and each matched LTR sequence were derived from the Dfam database and were aligned using the MUSCLE program.
