# Supplementary material for: Evolutionary lineage-specific genomic imprinting at the ZNF791 locus
Source: PLoS Genet. 2025 Jan 15;21(1):e1011532. doi: 10.1371/journal.pgen.1011532 (PMC11734915; doi:10.1371/journal.pgen.1011532)
Supplement: S21 Fig — (PDF) [file pgen.1011532.s021.pdf]

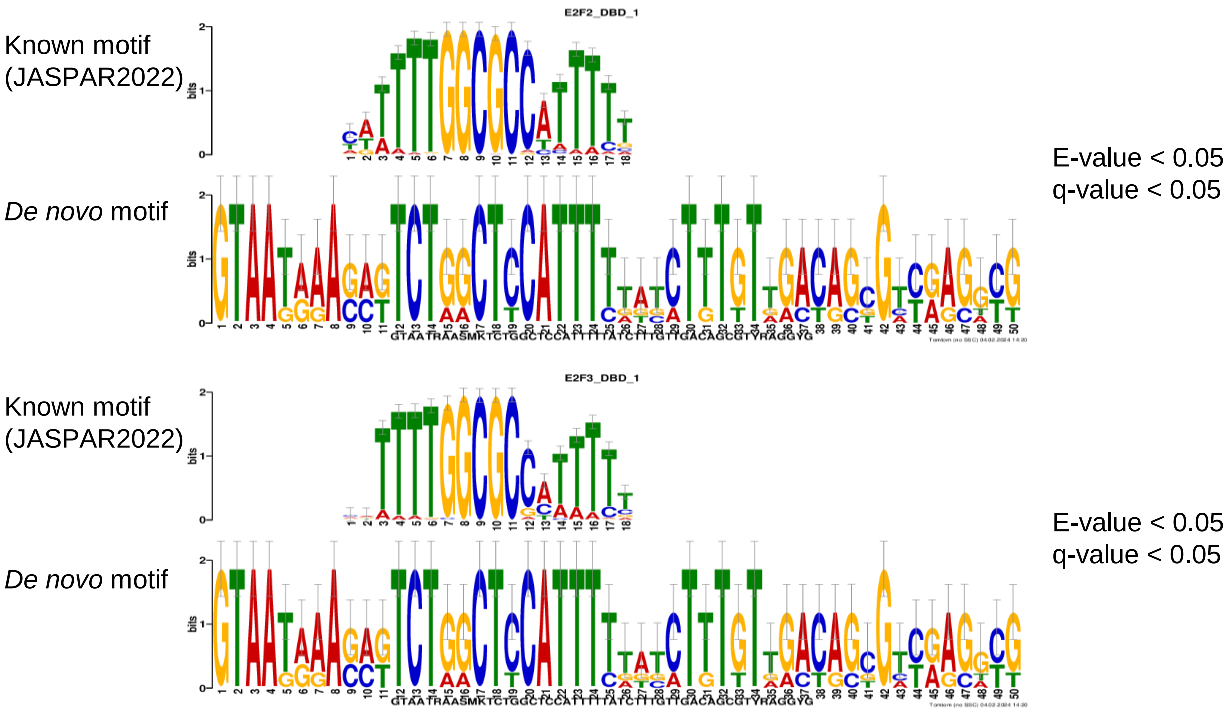

S21 Fig. Motif discovery and comparison. *De novo* motifs identified by the MEME tool that were common in five LTR52 sequences detected in this study (E-value < 0.05). They were found to be matched with known transcription factor binding motifs, E2F2 and E2F3 from the JASPAR2022 CORE vertebrates non-redundant database by the TOMTOM tool (E-value < 0.05, *q*-value < 0.05). The color for A and T were maintained as in the output, not converting to green and red, respectively, as in Fig 7.
