# Supplementary material for: Evolutionary lineage-specific genomic imprinting at the ZNF791 locus
Source: PLoS Genet. 2025 Jan 15;21(1):e1011532. doi: 10.1371/journal.pgen.1011532 (PMC11734915; doi:10.1371/journal.pgen.1011532)
Supplement: S22 Fig — (PDF) [file pgen.1011532.s022.pdf]

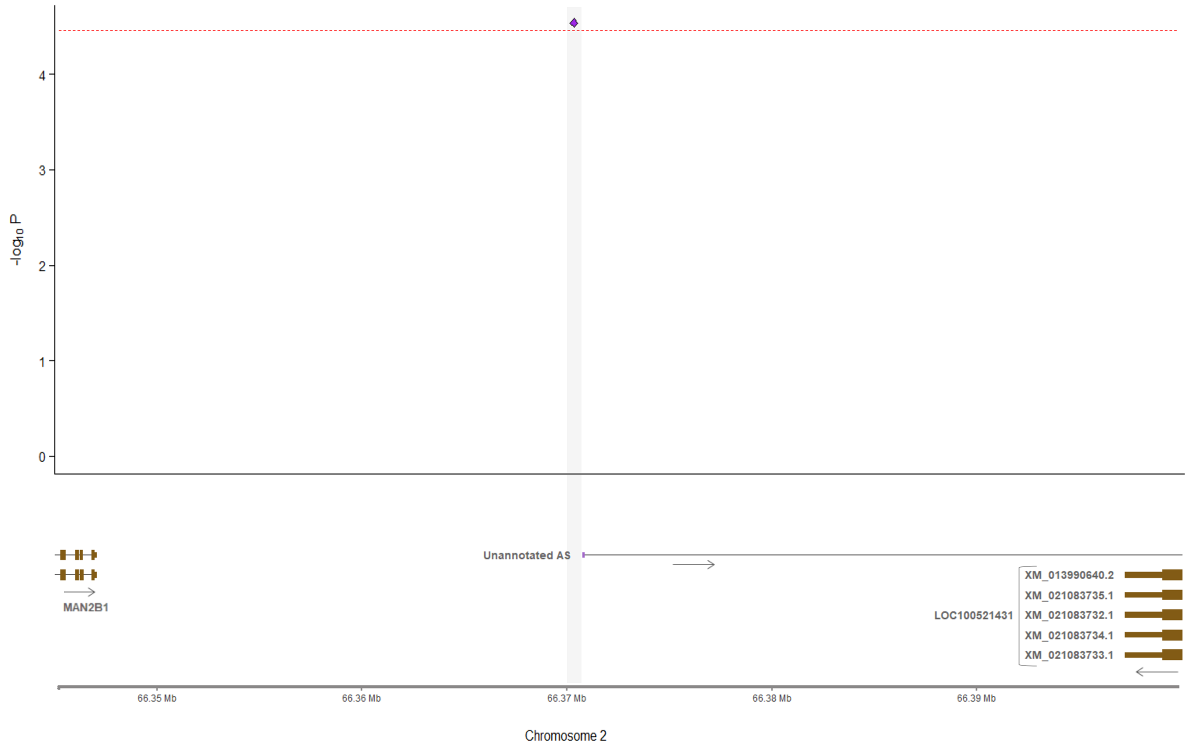

S22 Fig. QTL analysis for the upstream of the unannotated antisense transcript. Location (chr2:66370420) of a SNP (rs81272049) associated with residual feed intake (RFI) trait of pigs (*p*-value= 2.89×10^{-5}). The red dashed line shows suggestive significance with a *p*-value threshold of 3.09×10^{-5}. The suggestive significance and genome-wide significance were set as p = 1/N and p = 0.05/N, respectively, where N is the number of analyzed SNPs (Lander and Kruglyak, 1995). The genome-wide significance was *p*-value of 1.55×10^{-6}. The data were derived from pigQTLdb under http://www.animalgenome.org/QTLdb/ based on a recent GWAS (Li et al., 2022).
