# Supplementary material for: Evolutionary lineage-specific genomic imprinting at the ZNF791 locus
Source: PLoS Genet. 2025 Jan 15;21(1):e1011532. doi: 10.1371/journal.pgen.1011532 (PMC11734915; doi:10.1371/journal.pgen.1011532)
Supplement: S23 Fig — (PDF) [file pgen.1011532.s023.pdf]

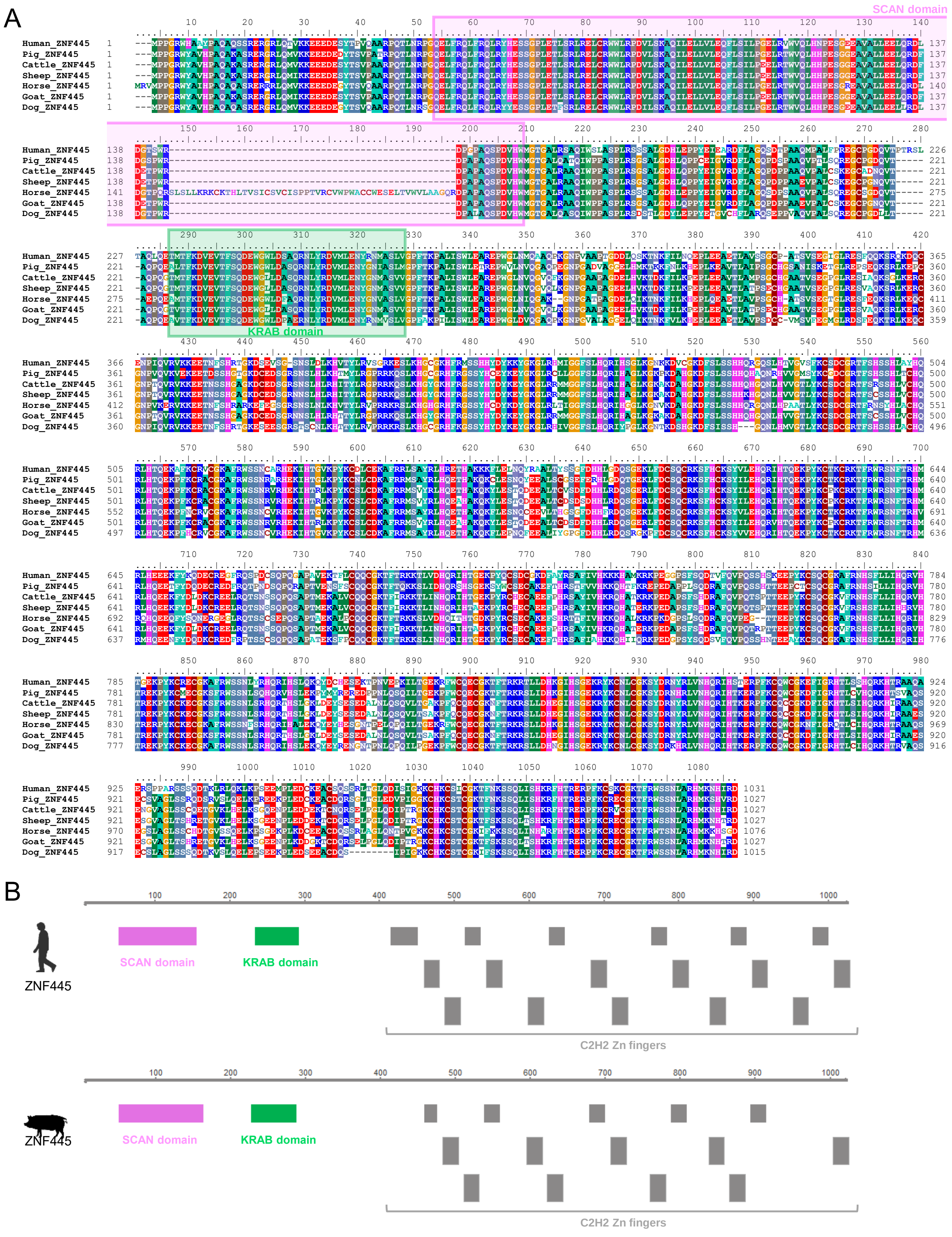

S23 Fig. Sequence and binding motif analysis. (A) Multiple sequence alignment of ZNF445 proteins performed using ClustalW in BioEdit. Pairwise BLAST between human and pig shows 77% identity and 85% similarity with conservative substitutions. Similarly, the pairwise BLAST between pig and other species show 82% identity and 88% similarity with cattle, 81% and 88% with sheep, 76% and 82% with horse, 82% and 88% with goat, and 80% and 87% with dog. A shading threshold of 50% was used on the figure. Protein IDs used: NP_852466.1 (Human), XP_005657124.1 (Pig), NP_001192748.1 (Cattle), XP_027813877.1 (Sheep), XP_023476384.1 (Horse), XP_005695656.2 (Goat), and XP_038287626.1 (Dog). **(B)** Structure of human and pig ZNF445 proteins.

C

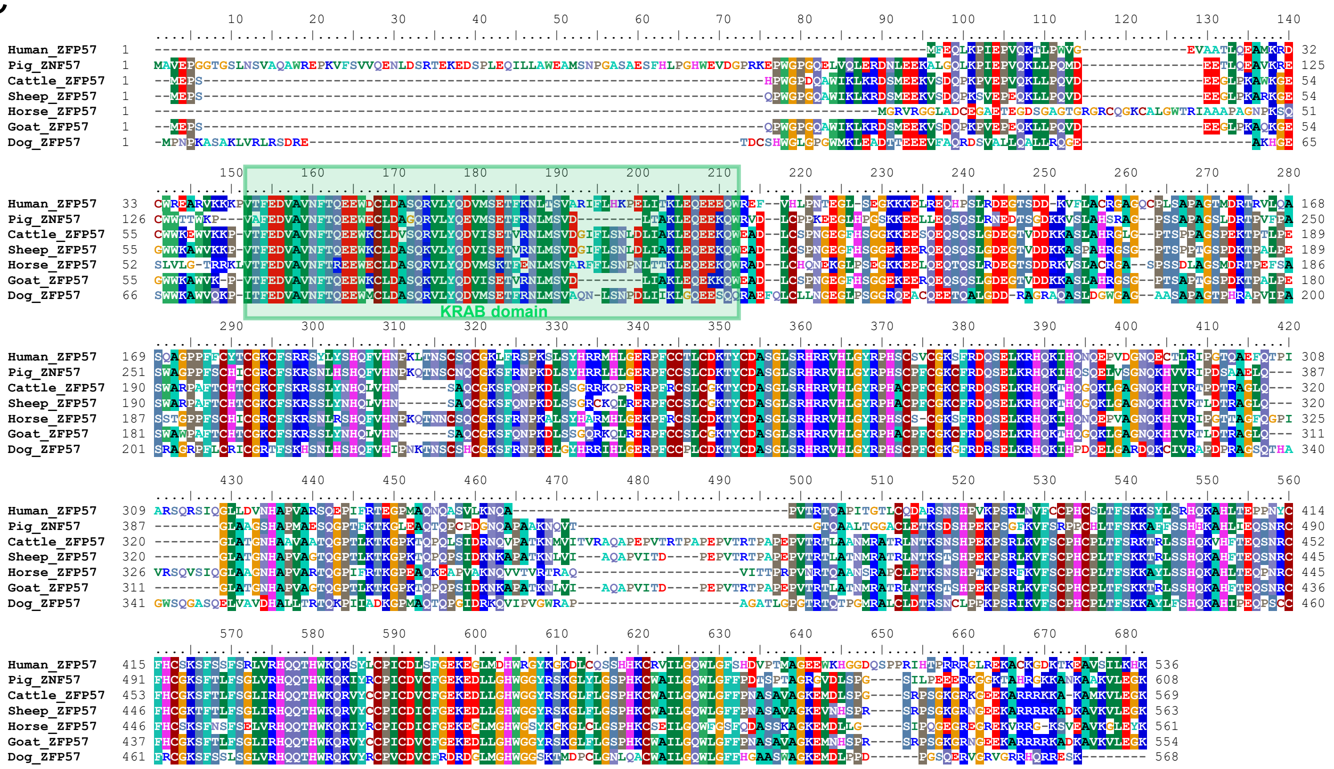

D

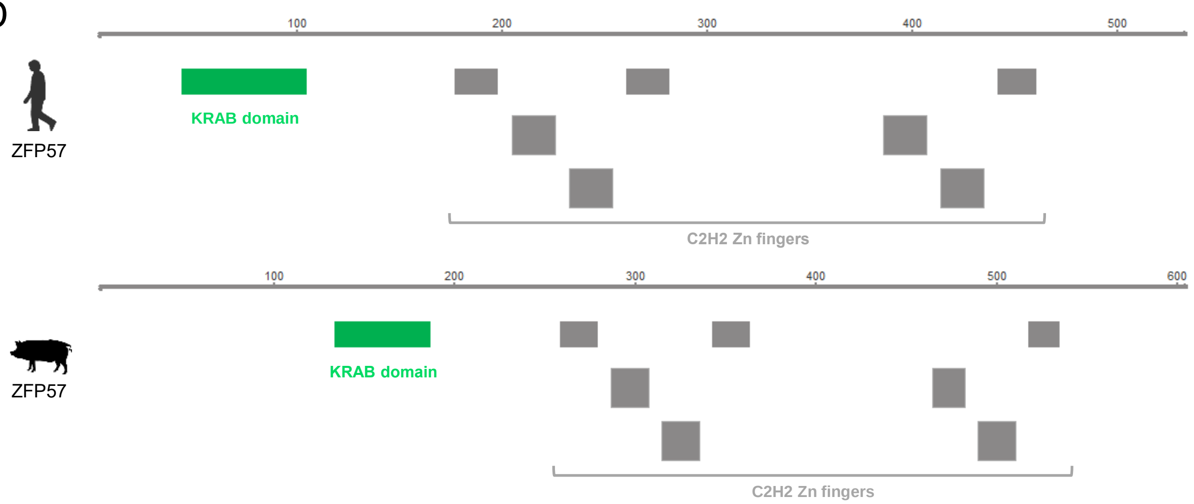

S23 Fig (Cont'd). (C) Multiple sequence alignment (MSA) for ZFP57 proteins was performed using ClustalW in BioEdit. Pairwise BLAST between human and pig shows 52% identity and 58% similarity including conservative substitutions. Similarly, the pairwise BLAST between pig and other species show 58% identity and 64% similarity with cattle, 58% and 65% with sheep, 56% and 61% with horse, 59% and 66% with goat, and 49% and 58% with dog. A threshold for shading was set to 50% for the figure. Protein IDs used: NP_001103279.2 (Human), NP_001116604.1 (Pig), XP_010816695.1 (Cattle), XP_042093259.1 (Sheep), XP_023480350.1 (Horse), XP_017894304.1 (Goat), and XP_038302587.1 (Dog). **(D)** Structure of human and pig ZFP57 proteins.

E

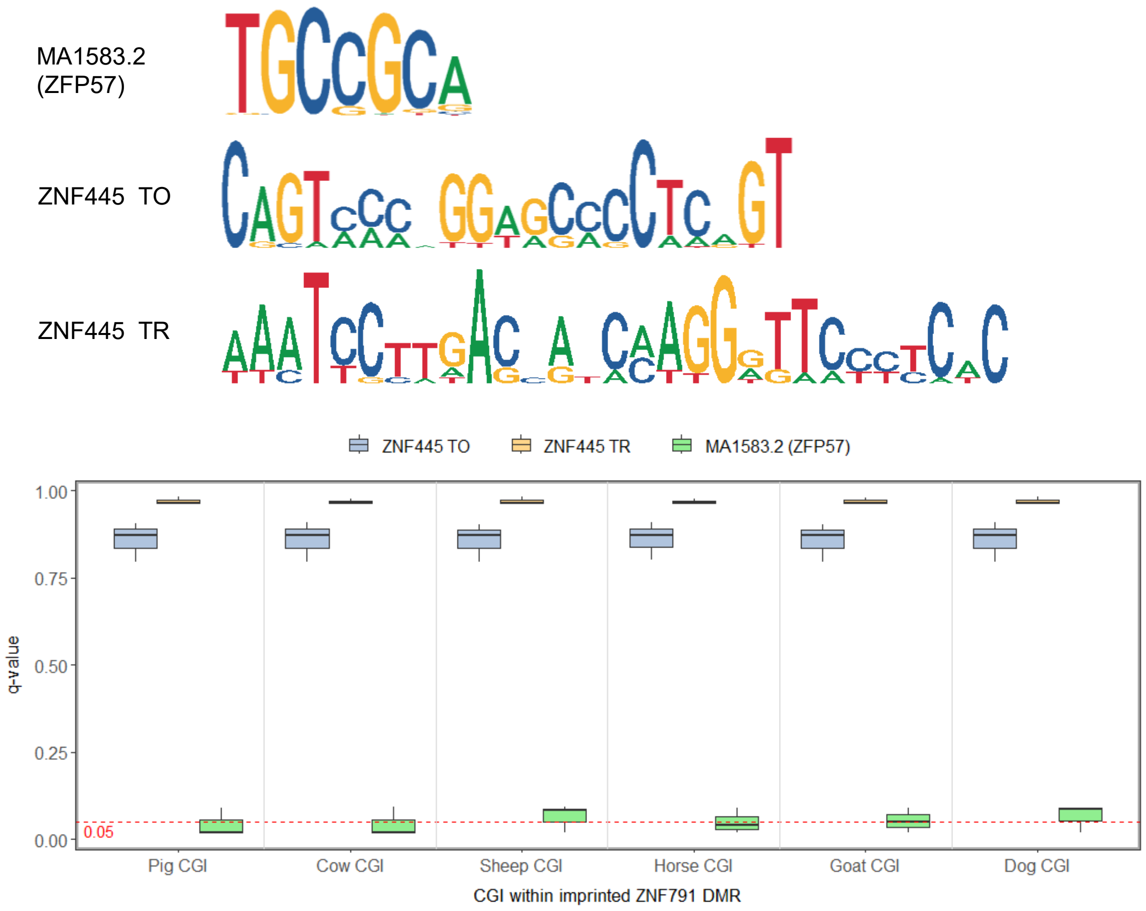

F

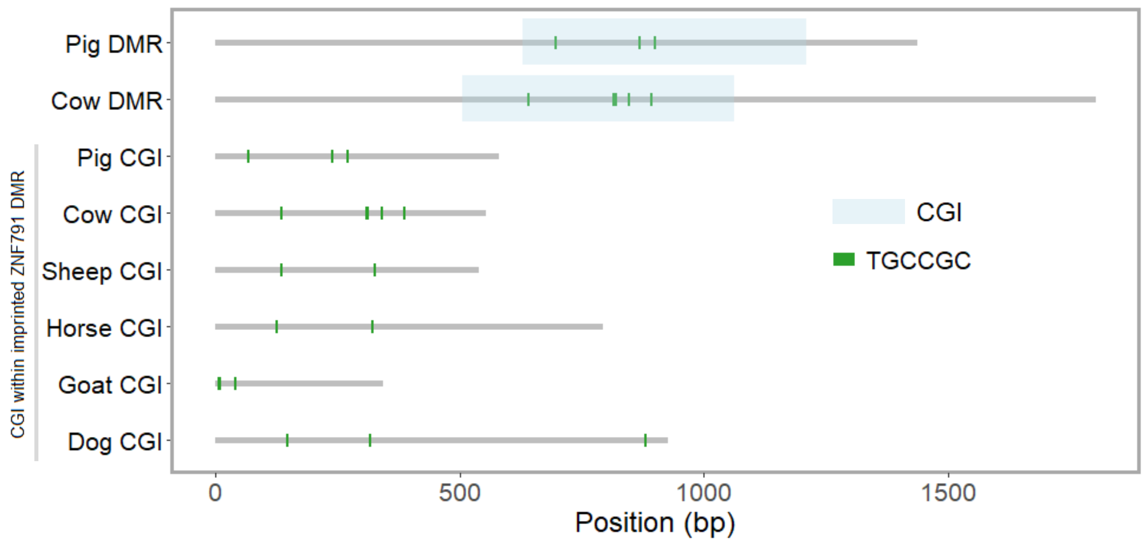

S23 Fig (Cont'd). (E) The consensus ZFP57 binding motif (MA1583.2.meme, JASPAR database), the predicted ZNF445 binding motifs (ZNF445 TO, Trono Original, and ZNF445 TR, Trono Reprocessed), and FIMO analysis of these motifs on CGIs within the imprinted *ZNF791* DMRs. Sites listed in S6 Table are plotted, and a *q-value* threshold of 0.05 is indicated by a red dashed line. **(F)** Locations of the ZFP57 binding TGCCGC motif with *q-values* < 0.05 in the pig *ZNF791-like* DMR (Fig 2C,F) and the cow *ZNF791* DMR (Fig 5H). CpG islands (CGIs), highlighted with red shading in Fig 7A, are further analyzed for the motif's locations (*q-values* < 0.05). Thick green bars in the cow and goat CGIs indicate the presence of two consecutive motifs.
