# Supplementary material for: Evolutionary lineage-specific genomic imprinting at the ZNF791 locus
Source: PLoS Genet. 2025 Jan 15;21(1):e1011532. doi: 10.1371/journal.pgen.1011532 (PMC11734915; doi:10.1371/journal.pgen.1011532)
Supplement: S11 Fig — (PDF) [file pgen.1011532.s011.pdf]

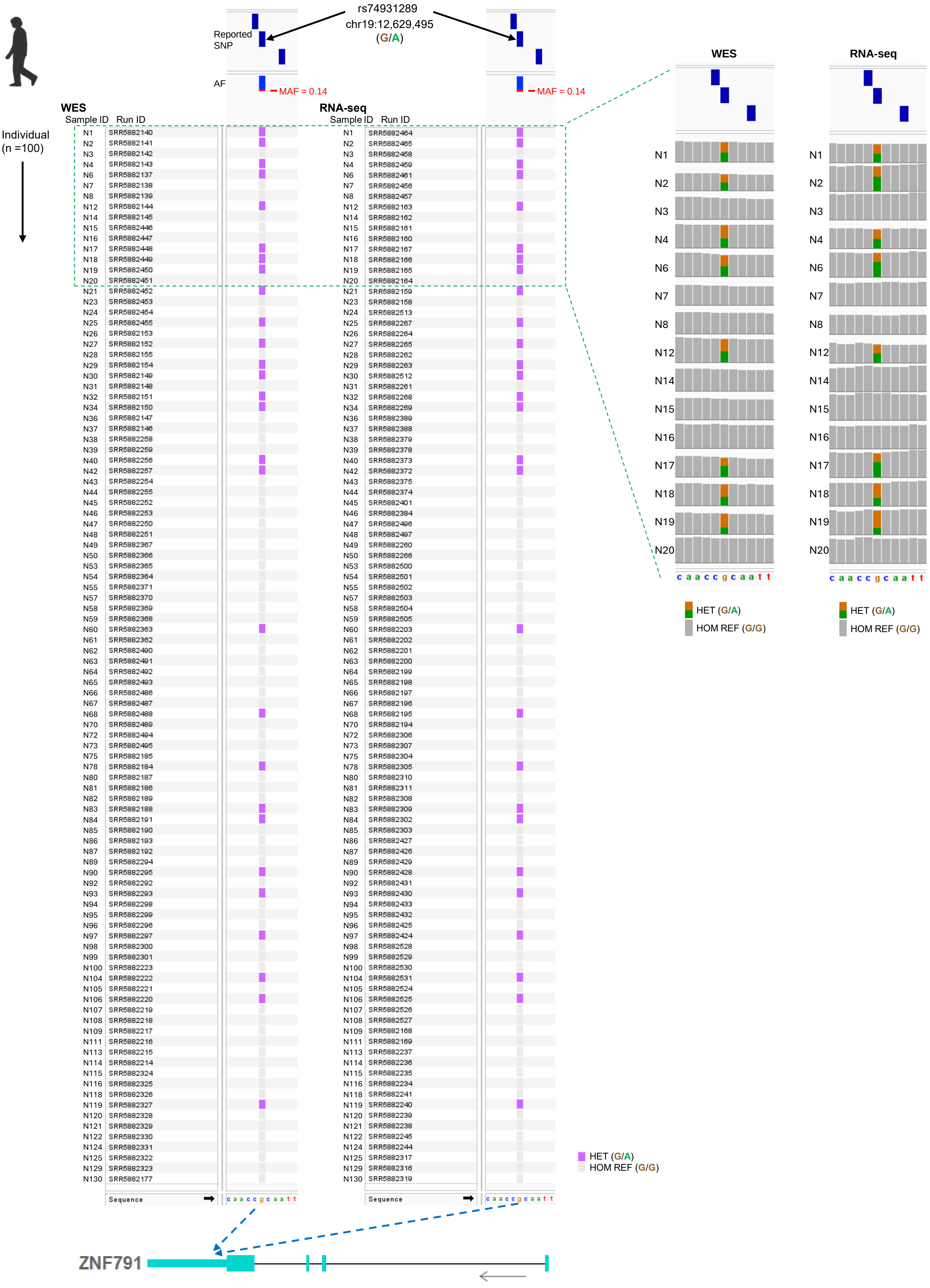

S11 Fig. Human lung exome from WES and RNA-seq. (A) SNP information for 100 individuals is shown using a VCF file. A SNP at the last exon (3′ UTR) of *ZNF791* [chr19:12,629,495; G/A (reference/alternate); rs74931289] was targeted. A minor allele frequency of this targeted SNP was 0.139 (MAF > 0.05). An informative, heterozygous SNPs (G/A) are indicated with blue. Grey indicates homozygous reference allele (G/G). The inset shows a close view of each SNP in lung tissues of each individual.
