# Supplementary material for: Evolutionary lineage-specific genomic imprinting at the ZNF791 locus
Source: PLoS Genet. 2025 Jan 15;21(1):e1011532. doi: 10.1371/journal.pgen.1011532 (PMC11734915; doi:10.1371/journal.pgen.1011532)
Supplement: Attachment — Submitted filename: 20240914_Review.pdf [file pgen.1011532.s030.pdf]

In this manuscript, the authors used parthenogenote embryos (consisting of 2 maternal genomes) and normal biparental embryos to evaluate the early mechanisms of parental genomic imprinting in the porcine species. Both aspects of genomic imprinting including parental-specific methylation and expression were analysed. The authors focused their analyses on Zinc Finger Proteins genes since they have been shown involved in DNA methylation changes during preimplantation development.

The first part of the manuscript highlighted a novel maternal differentially metylated region (DMR) spanning the promoter of the *ZNF791* gene, which seemed regulating its own paternal expression and the likely maternal expression of a novel antisens long non-coding RNA. The second part of the article showed the lineage-specific imprinting of this locus restricted to the laurasiatherian clade and absent from primates and rodents. And the last part was dedicated to the molecular mechanism demonstarting the acquisition of imprinting pattern in the *ZNF791* gene similarly to the *Sfmbt2* locus in mouse through histones deposition on LTR in germline maternal oocytes.

This manuscript suggested for the first time non-canonical imprinting in another clade than rodents.

Comments to the editor

The work presented here seems rigorous, the article is very well written with the story easy to follow, results are convincing and likely suited to Plos Genetics broadership. However, I have some concerns about specific points that I would like to be raised before any acceptance for publication.

1. questions regarding the model and the design

Authors used parthenogenotes embryos instead of more classical methods for genomic imprinting evaluation such as reciprocal crosses and informative variants. All experimentations and manipulations of embryos may have molecular, physiological and phenotypic impact on the whole embryo likely through DNA methylation modification. This is especially true for genomic imprinting regions since new borns from ART showed a higher risk og human imprinting disorders. Here, this assumption seems confirmed with siginificant differences for size and body weight between PA and CN.

- Firstly, what is the development stage in term of tissues or organes at the time of 21D when the embryos were collected ? Secondly, 10 PA and 10 CN seemed having collected based on supplementary figure 1. More informations have to be given (how many from each dam) ? Which ones have been used for analyses ? These crucial informations are missing in the manuscript.

- The study and the whole dataset might be much more powerful and convincing by analysing biological replicats instead of technical replicats. Are all embryos being analysed as suggested in the Figure 2 in which results are based on several embryos ? Are samples in RNAseq data technical or biological replicats ? There are misleading between material and methods and results shown (L437 and L493). Why only 2 WGBS PA and 2 WGBS CN and 3 RNAseq PA and 6 RNAseq CN ? Are they the same samples or just numbered differently ?

This has to be discussed because it is confusing from the very beginning and because the very interesting results of this manuscript are based on very few observations.

2. questions regarding the first characterization of the DMR, Figure 1 and Supplementary Table 2

Methods and results showed in the Figure 1 don't seem rigorous enough to reach the conclusion.

- L455 for CpG coverage analysis. Methylation percentages were based on a minimal coverage of 3 reads per CpG that is very low for DNA mathylation and especially for hemi-methylation identification. This has to be precised and the raw coverage of CpGs should be mentioned in the interesting regions (Figure 1C) as well the mean coverage in Supplementary Table 2.

- To conclude to an unbiaised detection of DMRs in the only 2 samples, the global DNA methylation levels should be analysed on all genes and expressed transcripts instead of focusing on *ZNF* loci (Figure 1A) and L122

- The way to present results is quite confusing and not supported by the following results. All PLAGL1 and ZNF791-like transcripts are shown but most of them have exactly the same expression and methylation values in Supplementary Table 2 suggesting that only some isoforms are expressed in embryos as shown later on. This has to be modified in consequence as well as the L135 where the conclusion is that 23 mRNA transcripts of *PLAGL1* and *ZNF791*-like were identified… that is false (Figure 1B).

- The top 88 ranked transcripts were selected but how many don't have orthologous genes in human or mouse ?

3. questions regarding the identification of an unannotated long non-coding RNA

Although the assembly of the pig genome is of high quality, its annotation might be improved and is even poor in imprinted regions and this particularly true for long non-coding RNAs. As examples, both *H19* and *KCNQ1OT1* are unannotated while they exist. Here, results are very interesting and very well detailed.

- Would it be possible to add on Figure 2 all intron-exon junctions for this novel antisens lncRNA ?

- The detailed characterization of this novel transcript in oocytes, embryos and 60-days old pig is highlighted in this species. However, nothing is mentionned later on when the evolutionary aspects of lineage-specific imprinting arose ? Could the authors discussed about that ?

4. Minor comments

- Bibliography could be updated with recent articles very important for the topic presented here

https://genomebiology.biomedcentral.com/articles/10.1186/s13059-023-02869-1
https://pubmed.ncbi.nlm.nih.gov/37309910/
https://pubmed.ncbi.nlm.nih.gov/38500688/

- Point regarding the size of the region and LTR repeats

I was wondering from the beginning of the study why *ZNF791-like* gene had several isoforms of different sizes with 2 important CpGs islands. While figures 3 and 4 were focused on the shortest isoform, we discovered later through the comparison between species that the two CpGs islands exist as well in primates and rodents but the longest isoforms seemed absent. Why do not include the whole locus in Figure 3 and 4 ?

Why a dedicated analysis has been performed to detect LTR while there are some data available on the various genome browsers ?

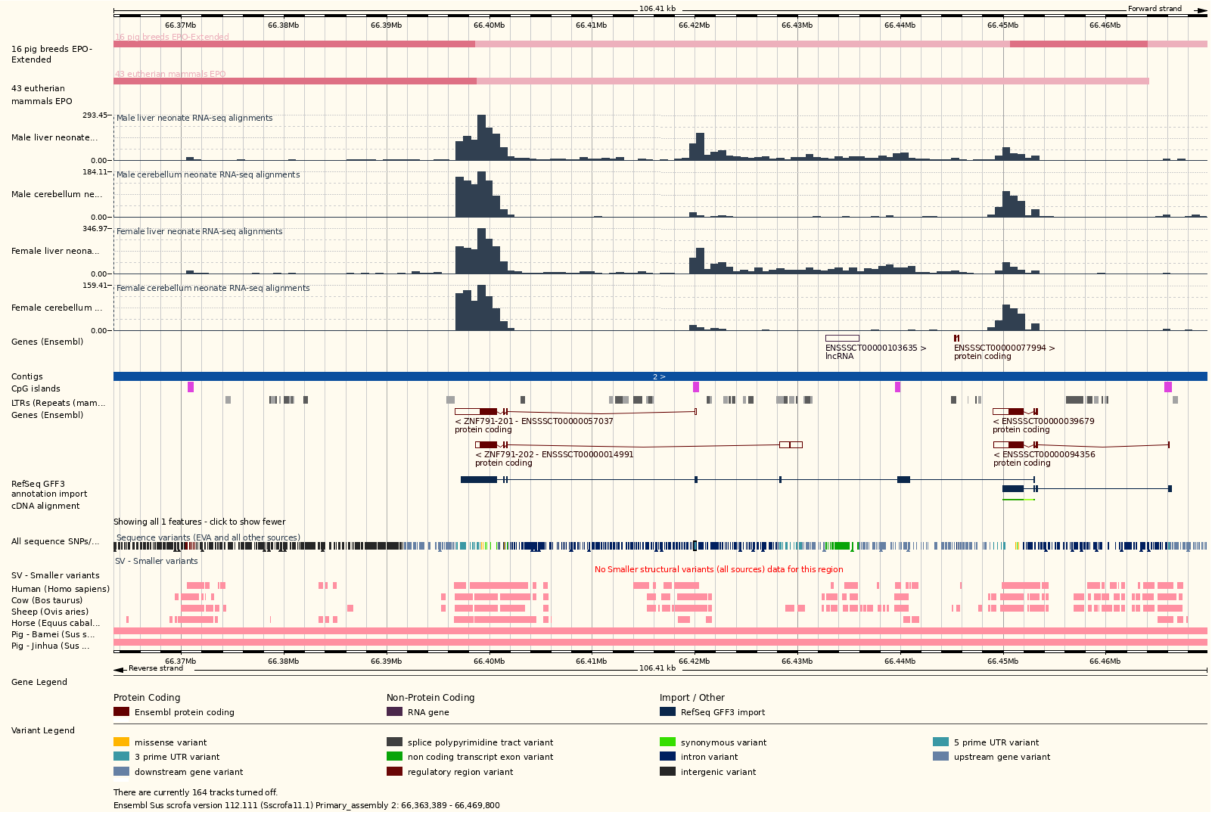

- Final point about the interest in pig production for feed efficiency (article of Li et al.)

That is true that the SNP rs81272049, located in the ZNF791 region was associated with RFI only in Yorkshire breed. However, this is one among a very long 8Mb haplotype including potential other imprinted genes. Consequently, this observation is important but might be moderated by suggesting that imprinted *ZNF791-like* gene belongs to a genomic regions which is associted with RFI phenotype.

- L516 - supplementary Table 3
