# Supplementary material for: Evolutionary lineage-specific genomic imprinting at the ZNF791 locus
Source: PLoS Genet. 2025 Jan 15;21(1):e1011532. doi: 10.1371/journal.pgen.1011532 (PMC11734915; doi:10.1371/journal.pgen.1011532)
Supplement: Attachment — Submitted filename: Reviewer_comments_RESPONSE_MM-JA.pdf [file pgen.1011532.s031.pdf]

Dear Editors,

Thank you for reviewing our manuscript (PGENETICS-D-24-00906), in which we describe genomic imprinting at the *ZNF791* locus across species. We have addressed the reviewers' comments and revised the manuscript accordingly. Additionally, we have formatted the figure labels according to PLOS Genetics guidelines and streamlined the figure legends while preserving essential information. We believe that these changes have strengthened the manuscript and that the explanations provided are appropriate. Below, we have provided our responses, outlined the revisions, and listed the updated references.

Reviewer's Responses to Questions

Comments to the Authors:
Please note here if the review is uploaded as an attachment.

Reviewer #1: Synopsis
In this study, the authors compare parthenogenetic and normal pig embryos to identify new imprinted genes. By limiting their search for Zn finger protein genes with human orthologs, they identify maternal gDMRS at the PLAGL1 and ZNF791-like genes. They show expression data suggesting that the ZNF791-like gene is exclusively expressed from the paternal allele. They show that the differential methylation at the ZNF791 gene is conserved in domesticated animals (pig, cow, goat, sheep, horse and dog) and absent in primates and rodents. Using expressed SNPs, they confirm monoallelic expression in species with the gametic DMR. Analysis of published data from pig and cow suggest the implication of an LTR element, active as a promoter in oocyte, in transcription across the DMR and de novo DNA methylation. This was not observed in human and mouse oocytes. Evidence for a similar LTR-driven mechanism is presented for other imprinted species. Overall, the study present good evidence that imprinting at ZNF791 in domesticated species correlates with integration of an LTR element at the locus.
The paper is generally well written and the interpretation and conclusions are generally well supported by the new data presented as well as the mining of published data and their analyses by the authors. However, the manuscript could be improved by addressing the following comments.

Comments
1. The authors limited their gDMR search to 642 zinc finger genes but do not provide any justification for this choice. Why only those genes were queried?

Ans) We have added our justification in the Introduction (lines 100-106). To facilitate locus-specific analysis, we focused on a subset of genes while ensuring a sufficient number for analysis, given that less than 1% of genes are generally estimated to be subject to imprinting (Tucci et al. 2019).

Thus, we selected the zinc finger protein gene family, which is one of the largest gene families. This reference number has been updated from [21] to [11].

[11] Tucci V, Isles AR, Kelsey G, Ferguson-Smith AC, Erice Imprinting G. 2019. Genomic Imprinting and Physiological Processes in Mammals. Cell 176: 952-965.

2. Similarly, from the top differentially methylated ZNF genes only those with human orthologs were considered. Why? The authors need to justify the basis for this selection.

Ans) We have included a justification of this selection in lines 134-136 and added a new tab ("top100_transcripts_sort_by_PA1") in S2 Table. Our aim was to explore similarities and differences between humans and domesticated animals, including pigs. However, the current pig reference genome (Sscrofa11.1/susScr11) is relatively incomplete, and many genes remain uncharacterized in the gene annotation. This limits our ability to conduct comprehensive cross-species comparisons, especially when orthologous genes are not well defined in pigs. Therefore, we focused on ZNF genes with known human orthologs for comparative analyses.

3. For the profiles presented in Fig.1C, the authors should annotate the X axes and show the position of the TSS and exons. The bottom tick marks presumably show the position of CpGs; this should be clarified on the figure.

Ans) Fig 1C has been revised to include X-axis annotations, the positions of TSS and first exons, as well as labeling of CpGs and CpG islands. Additionally, in response to Reviewer 3's comment #2, CpG coverages have also been added.

4. Line 139-140 - "and the corresponding paternal expression pattern was exclusively observed in the control embryos." The authors refer to this expression data but have not yet mentioned that they have conducted RNA-seq analysis in the paper. This should be presented first.

Ans) We added "our RNA-seq analysis showed that" in line 156.

5. Although the author suggest the formation of an antisense transcript, no strand-specific data is presented. Is the RNA-seq data stranded? If not, the proper annotation of this transcript would require usage of a strand-specific RT-PCR experiment.

Ans) Our RNA-seq data in Fig 2A is unstranded. We also identified that other pig RNA-seq data from oocytes (Fig 5A) and liver (S13C Fig) are unstranded (S13D Fig). Since the putative antisense transcript was observed in the liver, we analyzed stranded RNA-seq data from pig liver and

identified that the transcript is expressed in the opposite direction relative to *ZNF791-like*, as shown in the newly added S13E Fig.

We added the following text in lines 248-249: "In stranded RNA-seq data from the liver … was opposite to that of *ZNF791-like* (S13D and S13E Fig)".

6. Lines 183-186 - Although consistent with imprinting, differential methylation between oocyte and sperm is not "indicating that maternal silencing and paternal monoallelic transcriptional initiation of ZNF791". This would need to be confirmed by analysis of expression. Nevertheless, the DNA methylation data in somatic tissues do show that the gametic DMR is maintained and lend further support for imprinting of the locus. This needs to be clarified.

Ans) We revised the sentence in lines 205-207 to emphasize the conservation of imprinted gametic methylation, as there is no supporting expression data to confirm maternal silencing and paternal monoallelic expression.

7. Line 239-241 - The statement about the roles of H3K39 methylation in de novo DNA methylation in oocytes needs to refer to these two publications:
Xu, Q. et al. SETD2 regulates the maternal epigenome, genomic imprinting and embryonic development. Nature Genetics 51, 844–856 (2019).
Yano, S. et al. Histone H3K36me2 and H3K36me3 form a chromatin platform essential for DNMT3A-dependent DNA methylation in mouse oocytes. Nat Commun 13, 4440 (2022).

Ans) We cited these two papers (reference number [16, 17]) in line 271.

8. In the section starting on line 259, the authors make a parallel between what they observed at ZNF791-like genes and at the mouse Impact locus. In fact, two different mouse genes have been confirmed to acquired DNA methylation in oocytes as a consequence of transcription from an upstream LTR promoter, Impact and Slc38a4. This should be clarified, with reference to this publication, which is only mentioned in sFig.18 but not cited in the paper nor included in the bibliography:
Bogutz, A. B. et al. Evolution of imprinting via lineage-specific insertion of retroviral promoters. Nat Commun 10, 5674 (2019).
This also applies to the sentences starting on line 319 and 372, where the same reference should be included.

Ans) In lines 291-293, we added *Slc38a4* along with the citation for Bogutz et al. (2019) as reference [18], and also included a citation for the rat *Impact* gene (Okamura et al. 2005) as reference [19]. Additionally, in line 354 we added the references [18, 19], and in line 423, we added the reference [18].

[18] Bogutz, A. B., J. Brind'Amour, H. Kobayashi, K. N. Jensen, K. Nakabayashi, H. Imai, M. C. Lorincz, and L. Lefebvre. 2019. Evolution of imprinting via lineage-specific insertion of retroviral promoters. Nat. Commun. 10(1):5674.

[19] Okamura K, Sakaki Y, Ito T. 2005. Comparative genomics approach toward critical determinants for the imprinting of an evolutionarily conserved gene Impact. Biochem. Biophys. Res. Commun. 329: 824-830.

9. Lines 314-318 - It is not clear to this reviewer why the analysis presented is "contrary to" to emergence of imprinting after the split from monotremes. In fact, it is consistent with this model, since imprinting of ZNF791 is more recent. This needs to be fixed in the text.

Ans) We originally intended to express that the imprinting of ZNF791 occurred on a different time scale compared to the emergence of imprinting. However, we agree that "Contrary to" was not the appropriate phrasing. Based on the reviewer's suggestion, we have replaced "Contrary to" with "In addition to" to better reflect the timing of these evolutionary events (lines 347-348).

10. Line 363 - Although an RNAi silencing may occur here, the downregulation of the paternal allele of the antisense transcript could also be mediated via a transcriptional interference mechanism implicating enhancer occlusion by ZNF971, as proposed at several loci in this publication:
Pande, A., Makalowski, W., Brosius, J. & Raabe, C. A. Enhancer occlusion transcripts regulate the activity of human enhancer domains via transcriptional interference: a computational perspective. Nucleic Acids Res. 48, 3435–3454 (2020).

Ans) We have revised the sentences in lines 398-408 to address this point, and we have added the suggested reference as [22].

11. Line 373 - The original suggestion of a link between genomic imprinting and a defense mechanism comes from this publication by Denise Barlow, which predates reference #16 given by the authors:
Barlow, D. P. Methylation and Imprinting: from Host Defense to Gene Regulation? Science 260, 309–310 (1993).

Ans) Thank you for pointing this out. We have added this reference as [26] in line 425 to properly credit the initial suggestion of a link between genomic imprinting and a defense mechanism.

12.Line 375 - In addition to reference #17, the authors also need to refer here to the Bogutz reference given above in point 8.

Ans) We have cited Bogutz et al., 2019 as reference [18] in this sentence as well (line 426). Additionally, the reference previously numbered [17] has been updated to [24].

13.Line 378 - A reference to the Xu paper mention above (point 7) needs to be added here.

Ans) We have added Xu et al., 2019 as reference [16] at this point in line 435.

14. Line 385 - Protection from demethylation may involve ZFP57/445 or similar DNA methylation-dependent binding of a Zn finger protein. This should be mentioned here. The authors should mention whether these proteins are conserved in the domesticated species studied here and whether the imprinted ZNF791 DMRs contain binding motifs for these proteins.

Ans) We have addressed these factors in lines 442-445 by expanding the discussion on sequence conservation and binding motifs, based on a newly added S23 Fig. We have newly added references [30] and [31], along with updating the previous citation from [11] to [14].

[30] Quenneville S, Verde G, Corsinotti A, Kapopoulou A, Jakobsson J, Offner S, et al. In embryonic stem cells, ZFP57/KAP1 recognize a methylated hexanucleotide to affect chromatin and DNA methylation of imprinting control regions. Mol Cell. 2011;44(3):361-72.

[31] Takahashi N, Coluccio A, Thorball CW, Planet E, Shi H, Offner S, et al. ZNF445 is a primary regulator of genomic imprinting. Genes Dev. 2019;33(1-2):49-54.

15.Line 395 - Here again, the statement about imprinting evolution is confusing. The model is not that ALL imprinting occurred ~160 MYA ago, but rather that the first examples of imprinting are observed at this time. The fact that lineage-specific insertion of LTRs may have contributed to the emergence of species-specific imprinting at different time point since then is to be expected. In fact, examples of such events unique to the primate lineages have also been presented at 17 loci by Bogutz et al.

Ans) The sentence in lines 454-457 has been streamlined for clarity, addressing the confusion.

16.I do not recall this being mentioned in the paper, but the authors should clarify whether the documented LTR promoters are indeed transcriptionally oriented towards ZNF791.

Ans) To address the orientation of the LTR promoters, we added a section titled “8. Summary of gene and LTR orientations” in S16 Fig. Also, we clarified this by adding the following sentences in

lines 428-433: "All *ZNF791-like* and *ZNF791* genes in analyzed livestock ... reference [18]." Here, reference [18] corresponds to Bogutz et al. 2019.

Minor comments
1. The developmental stage of the embryos collected (21 days after the onset of estrus) should be mentioned in sFig. 1.
Ans) This developmental stage (21 days after the onset of estrus) has been mentioned in S1 Fig.
2. Line 148, change "was explored to reveal the imprinting status" to "was explored to study its imprinting status", to reflect that the imprinted expression was not yet shown in the paper.
Ans) We have replaced "reveal" with "study" in Line 167.
3. sFig.2 - "Non-expressed PLAGL1 transcripts are covered with white shading for visual distinction." It seems that the author actually mean transcripts not expressed in their embryonic samples. This should be clarified.
Ans) We have clarified this by specifying "embryonic samples" in the description of in S2C Fig.
4. In sFig.3, the position of the CpG islands should be presented.
Ans) The CpG islands have been added to S3 Fig.
5. Line 202 - This is a reference to Fig. 3G, not 3F. Otherwise, there is no reference to 3F in the text.
Ans) The references to Fig 3F and 3G have been corrected accordingly (lines 226-229).

Reviewer #2: In this manuscript, the authors generate transcriptome and methylome datasets in parthenogenetic porcine embryos to identify potentially novel imprinted genes. They use this data and previously published datasets from other species to reveal for the first time the species-specific imprinting of ZNF791 in domesticated animals but not human and mouse. The authors also convincingly show that the imprinting of ZNF791 correlates with the insertion of an LTR element that drives upstream transcription in the oocyte.

The manuscript is clear and convincing. To my opinion, it represents a nice and important contribution to the field of genomic imprinting that is of general interest for the scientific community.

I have only minor comments that need to be addressed for publication, as detailed below.

Comments:
-What is the rationale for initially focusing the analysis on zinc finger protein genes? Are these prone to imprinting in other species? This could be better explained in the introduction.

Ans) We have provided our rationale in the Introduction (lines 100-106). Initially, we did not specifically consider whether zinc finger protein genes are prone to imprinting in other species. Instead, we based our analysis on the general estimate that less than 1% of all genes are subject to imprinting (Tucci et al. 2019). This reference number has been updated from [21] to [11].

[11] Tucci V, Isles AR, Kelsey G, Ferguson-Smith AC, Erice Imprinting G. 2019. Genomic Imprinting and Physiological Processes in Mammals. Cell 176: 952-965.

-The WGBS datasets seem to be of good quality with a high number of reads. Can the authors provide some information on the sequencing depth and genome coverage?

Ans) Information on sequencing depth and genome coverage has been added to lines 125-126 and S1 Table.

-Figure 1B "Based on the methylation ratios between PA and CN embryos, the top 88 porcine zinc finger transcripts with orthologous loci in humans were identified".
Please be more specific. How where the top 88 candidate transcripts identified? Are these the 88 transcripts with the most methylation difference or only the ones with orthologous loci in humans? What cut off was used ? this is not clear.

Ans) We have revised this part of the manuscript (lines 134-137) and added a new tab ("top100_transcripts_sort_by_PA1") in S2 Table. From an initial selection of the top 100 transcripts based on methylation differences (ratios), we identified 88 candidate transcripts with human orthologous loci. The top 100 transcripts had a minimum methylation ratio of approximately 1.2.

-Figure 1C: In addition to the smoothed methylation profiles, can the authors also show the real methylation at CpG resolution for this region (a zoom in view of the DMR from data in Figure 2C)?

Ans) A zoom-in view of the methylation levels at CpG resolution for the DMR region has been incorporated into Fig 2F, as requested. Additionally, lines 194-195 have been added to the manuscript to describe this figure.

-Lines 159-160 "In contrast, the CGI regulates transcription of the ENSSSCG00000033388 gene was unmethylated in both PA and CN embryos (Fig. 2C)". Please correct, there seem to be a grammatical mistake.
Ans) We have corrected this grammatical mistake in line 178.

-Lines 180-186: in the text, the authors directly correlate hypomethylation with transcription initiation of the gene in gametes. I would tone this down because there is no expression data. The authors should only mention lack or conservation of imprinted gametic methylation.

Ans) We revised the sentences between lines 203-207 to focus on the lack or conservation of imprinted gametic methylation, as there is no supporting expression data.

-The analysis of individual reads to confirm the co-occurrence of fully methylated and hypomethylated sequences in DMRs (Figure 3E-F) is appreciated. However, I don't understand how these tendency figures were generated from the real WGBS datasets. More details need to be provided to make sure that these profiles rigorously reflect the reality of all the WGBS reads.
Ans) We have added supplementary data along with detailed explanations (S4C–S4E Fig) and revised the Methods section (lines 561-563).

-Have the authors tried to use SNPs in the DMR region to demonstrate parental-specific methylation in the existing WGBS datasets of somatic tissues from domestic animals?
Ans) We have not yet tried this approach, but we may consider developing strategies to investigate it depending on SNP densities and the availability of reference SNP panels for domestic animals.

-Lines 200-204: Figures 3F and 3G are not correctly cited in the text.
Ans) This issue has been corrected in lines 226-229.

-Line 516: I think "Supplementary Table 2" should be replaced by "Supplementary Table 3".

Ans) Thank you for pointing this out. We have corrected this mistake by replacing "Supplementary Table 2" with "S3 Table" throughout the paragraph at lines 590, 596, and 598.

Reviewer #3: The review is uploaded as an attachment

In this manuscript, the authors used parthenogenote embryos (consisting of 2 maternal genomes) and normal biparental embryos to evaluate the early mechanisms of parental genomic imprinting in the porcine species. Both aspects of genomic imprinting including parental-specific methylation and expression were analyzed. The authors focused their analyses on Zinc Finger Proteins genes since they have been shown involved in DNA methylation changes during preimplantation development.

The first part of the manuscript highlighted a novel maternal differentially methylated region (DMR) spanning the promoter of the *ZNF791* gene, which seemed regulating its own paternal expression and the likely maternal expression of a novel antisens long non-coding RNA. The second part of the article showed the lineage-specific imprinting of this locus restricted to the laurasiatherian clade

and absent from primates and rodents. And the last part was dedicated to the molecular mechanism demonstarting the acquisition of imprinting pattern in the *ZNF791* gene similarly to the *Sfmbt2* locus in mouse through histones deposition on LTR in germline maternal oocytes.

This manuscript suggested for the first time non-canonical imprinting in another clade than rodents.

Comments to the editor

The work presented here seems rigorous, the article is very well written with the story easy to follow, results are convincing and likely suited to Plos Genetics broadership. However, I have some concerns about specific points that I would like to be raised before any acceptance for publication.

1. questions regarding the model and the design

Authors used parthenogenotes embryos instead of more classical methods for genomic imprinting evaluation such as reciprocal crosses and informative variants. All experimentations and manipulations of embryos may have molecular, physiological and phenotypic impact on the whole embryo likely through DNA methylation modification. This is especially true for genomic imprinting regions since newborns from ART showed a higher risk of human imprinting disorders. Here, this assumption seems confirmed with significant differences for size and body weight between PA and CN.

• Firstly, what is the development stage in term of tissues or organs at the time of 21D when the embryos were collected? Secondly, 10 PA and 10 CN seemed having collected based on supplementary figure 1. More information has to be given (how many from each dam)? Which ones have been used for analyses? This crucial information is missing in the manuscript.

Ans) Although we did not perform histological analysis with 21D embryos, our previous results with 26D embryos showed that the organ and tissue formation was comparable between PA and CN embryos (Hwang et al. 2020). This reference has been added as [44]. In L499-501, we added a sentence: "Additionally, our previous histological assays showed that … although PA embryos were smaller than the control [44].".

Ten embryos from each of the PA and CN group were recovered from two dams, with half collected from each dam (L494-496). Embryos with intact morphology from both the PA and CN groups were used (L501-502).

[44] Hwang IS, Park MR, Lee HS, Kwak TU, Son HY, Kang JK, Lee JW, Lee K, Park EW, Hwang S. 2020. Developmental and Degenerative Characterization of Porcine Parthenogenetic Fetuses during Early Pregnancy. *Animals (Basel)* **10**.

• The study and the whole dataset might be much more powerful and convincing by analysing biological replicats instead of technical replicats. Are all embryos being analysed as suggested in the Figure 2 in which results are based on several embryos ? Are samples in RNAseq data technical or biological replicats ? There are misleading between material and methods and results shown

(L437 and L493). Why only 2 WGBS PA and 2 WGBS CN and 3 RNAseq PA and 6 RNAseq CN ? Are they the same samples or just numbered differently ?

Ans) The results in Fig 2 are based on several embryos. The RNA-seq data include biological replicates, but *not* technical replicates. For WGBS, the previous statement at L437 is correct (two biological replicates), and we have clarified it by stating, "two biological replicates for each of the PA and CN groups" (now at L507-508). For RNA-seq, the previous statement at L493 was misleading due to our mistake, so we have revised it to "three biological replicates for the PA group and six biological replicates for the CN group" (now at L566-567).

For WGBS, the number of replicates was determined by the availability of samples and the ENCODE standards, which require a minimum of two biological replicates with at least 30× coverage (III. Performance of WGBS Sequence Experiment: Number of replicates and sequencing depth in: https://www.encodeproject.org/documents/108d2515-c053-4b18-bc65-27e8f26d62c5/). This is discussed in L368-372 for the WGBS samples. For RNA-seq, we included more samples to clearly show the paternal expression pattern, as mentioned in L386-388. These are *not* the same samples *but* a different set of whole embryos, as noted in L388-389, and each sample has a unique identifying number.

This has to be discussed because it is confusing from the very beginning and because the very interesting results of this manuscript are based on very few observations.

Ans) We believe that we have addressed this concern in the lines mentioned above (L507-508, L566-567, L368-372, and L386-389). Additionally, the consistent results we obtained from processing publicly available data prompted us to prepare this manuscript.

2. questions regarding the first characterization of the DMR, Figure 1 and Supplementary Table 2

Methods and results showed in the Figure 1 don't seem rigorous enough to reach the conclusion.

• L455 for CpG coverage analysis. Methylation percentages were based on a minimal coverage of 3 reads per CpG that is very low for DNA methylation and especially for hemi-methylation identification. This has to be precised and the raw coverage of CpGs should be mentioned in the interesting regions (Figure 1C) as well the mean coverage in Supplementary Table 2.

Ans) We have revised L526-527 in the *Methods* section to clarify the CpG coverage criteria. Also, in L147-148 of the *Results* section, we noted that individual CpG coverages tended to exceed the minimum value of three by a substantial margin, supporting the validity of our conclusions. Fig 1C has been updated to show the raw CpG coverages for each sample, with further details provided in S2A and S2B Fig (L148-150). Moreover, the mean CpG coverages for all transcript isoforms for each sample are included in S2 Table (L150-151).

• To conclude to an unbiaised detection of DMRs in the only 2 samples, the global DNA methylation levels should be analysed on all genes and expressed transcripts instead of focusing on *ZNF* loci (Figure 1A) and L122

Ans) We replaced "unbiased" with "robust", because, although we focused on *ZNF* loci, the methylation profiles observed in the putative promoter regions were representative of typical methylation patterns found in promoter regions (L131).

• The way to present results is quite confusing and not supported by the following results. All PLAGL1 and ZNF791-like transcripts are shown but most of them have exactly the same expression and methylation values in Supplementary Table 2 suggesting that only some isoforms are expressed in embryos as shown later on. This has to be modified in consequence as well as the L135 where the conclusion is that 23 mRNA transcripts of *PLAGL1* and *ZNF791*-like were identified... that is false (Figure 1B).

Ans) In S2 Table, we provided DNA methylation levels for the putative promoter regions (TSS ± 2 kb) of all zinc finger protein gene transcripts but did *not* include gene expression values. We have added a sentence in L138-139 stating, "while methylation levels can be identical if the putative 4 kb promoter regions overlap and share CpG sites."

These were initial methylation profiling procedures based on WGBS. To avoid confusion, we changed the subtitle from "Detection of Differentially Methylated Regions and Imprinted Expression" to "DNA Methylation Profiling and Detection of Differentially Methylated Regions" in L122. Also, L141 was revised to "During the initial methylation profiling ..."

In L152-153, we replaced "23 mRNA transcripts of *PLAGL1* and *ZNF791-like* were identified to be..." with "23 mRNA transcripts of *PLAGL1* and *ZNF791-like* were initially categorized as candidate transcripts that might be..."

In L157-158, we added the phrase "for a subset of transcripts with the IDs XM_005654368.3, XM_005654371.3, and XM_021084067.1" to describe expressed *PLAGL1* transcripts based on RNA-seq data in S2C Fig.

• The top 88 ranked transcripts were selected but how many don't have orthologous genes in human or mouse ?

Ans) In L134-137, we clarified our procedure. Specifically, we sorted all ZNF transcripts and initially selected the top 100. Among these, 12 transcripts from a single gene do not have an orthologous gene in humans, as summarized in a new tab ("top100_transcripts_sort_by_PA1") in S2 Table.

3. questions regarding the identification of an unannotated long non-coding RNA

Although the assembly of the pig genome is of high quality, its annotation might be improved and is even poor in imprinted regions and this particularly true for long non-coding RNAs. As examples,

both *H19* and *KCNQ1OT1* are unannotated while they exist. Here, results are very interesting and very well detailed.

• Would it be possible to add on Figure 2 all intron-exon junctions for this novel antisense lncRNA ?

Ans) We have added all intron-exon junctions in Fig 2D for this antisense transcript. Additionally, in response to Reviewer1's comment #5, we have included S13D and S13E Fig, which show transcriptional directions from stranded RNA-seq data, while our RNA-seq data in Fig 2D was unstranded.

• The detailed characterization of this novel transcript in oocytes, embryos and 60-days old pig is highlighted in this species. However, nothing is mentioned later on when the evolutionary aspects of lineage-specific imprinting arose ? Could the authors discussed about that ?

Ans) Our discussion on this novel antisense transcript has been added in L409-414. As mentioned, the evolutionary aspects of this imprinting require further in-depth investigation.

4. Minor comments

• Bibliography could be updated with recent articles very important for the topic presented here

https://genomebiology.biomedcentral.com/articles/10.1186/s13059-023-02869-1

https://pubmed.ncbi.nlm.nih.gov/37309910/

https://pubmed.ncbi.nlm.nih.gov/38500688/

Ans) Thank you for sharing these recent and relevant papers. We have cited the first two in L457-459 and the last one, which reviews livestock imprinting, in L461-463.

• Point regarding the size of the region and LTR repeats

I was wondering from the beginning of the study why *ZNF791-like* gene had several isoforms of different sizes with 2 important CpGs islands. While figures 3 and 4 were focused on the shortest isoform, we discovered later through the comparison between species that the two CpGs islands exist as well in primates and rodents but the longest isoforms seemed absent. Why do not include the whole locus in Figure 3 and 4 ?

Ans) The longest isoforms appear to be transiently expressed during the oocyte stages in pigs and cattle but are likely absent in human and mouse oocytes. Therefore, in Figs 3 and 4, we focused on isoforms that are actually expressed in the later stages of development, specifically the shortest isoform. In line with this, the shortest isoforms are predominant in dogs, cattle, and pigs (S13A–S13C and S13E Fig), in sheep, horses, and goats (S19 Fig), as well as in porcine embryos (Fig 2).

For this subject, in L417, we have additionally cited two relevant papers, [18] and [24], and referenced S18 Fig, which is adapted from reference [18] that describes the mechanism of transcription. Also, we have newly added a review by Ferguson-Smith & Bourc'his (2018) as reference [25] in L417. Based on the following statement from this review, we expect that the LTR is active in pig and cow oocytes, promoting the expression of the longest transcript that overlaps the intragenic CpG island where hypermethylation is established as a DMR:

"… long terminal repeat (LTR) sequences of specific retrotransposons are particularly active during oogenesis. Through their promoter activity, they can define new transcription start sites in oocytes and promote transcription-coupled DNA methylation of downstream CpG islands."

[25] Ferguson-Smith AC, Bourc'his D. 2018. The discovery and importance of genomic imprinting. Elife 7. doi: 10.7554/eLife.42368.

Why a dedicated analysis has been performed to detect LTR while there are some data available on the various genome browsers ?

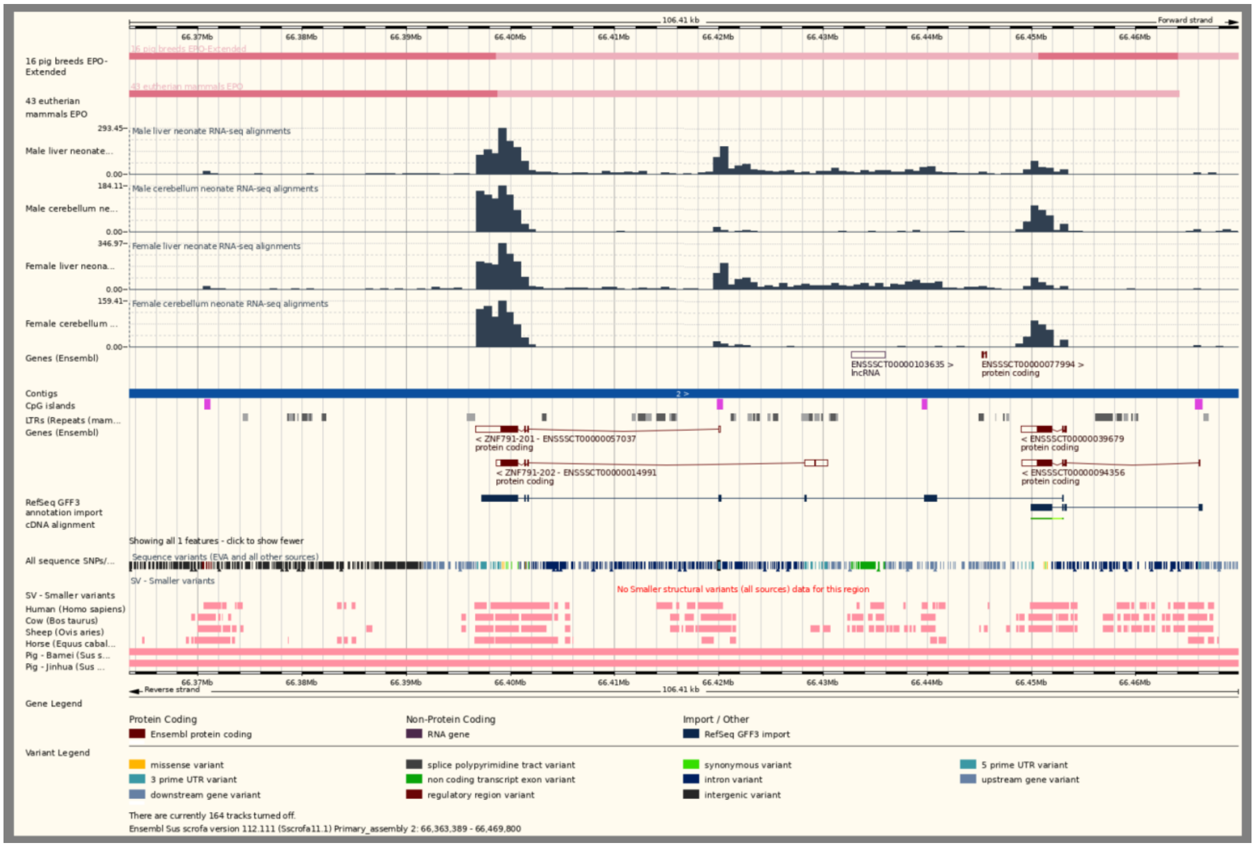

Ans) We initially searched the UCSC genome browser but could not find LTRs in the region of interest (indicated by the grey bar in the figure below). However, we identified LTRs within the same region using the Dfam database, as highlighted by the cyan bar overlapping the exon in the figure

below. It appears that the Ensembl LTR annotations provided by the reviewer are similar to UCSC's, as LTRs are also absent in this region (shown by the grey bar). This comparison has been added to S16 Fig ("9. Comparison of gene and LTR annotations in Ensembl, NCBI, UCSC, and Dfam for pigs").

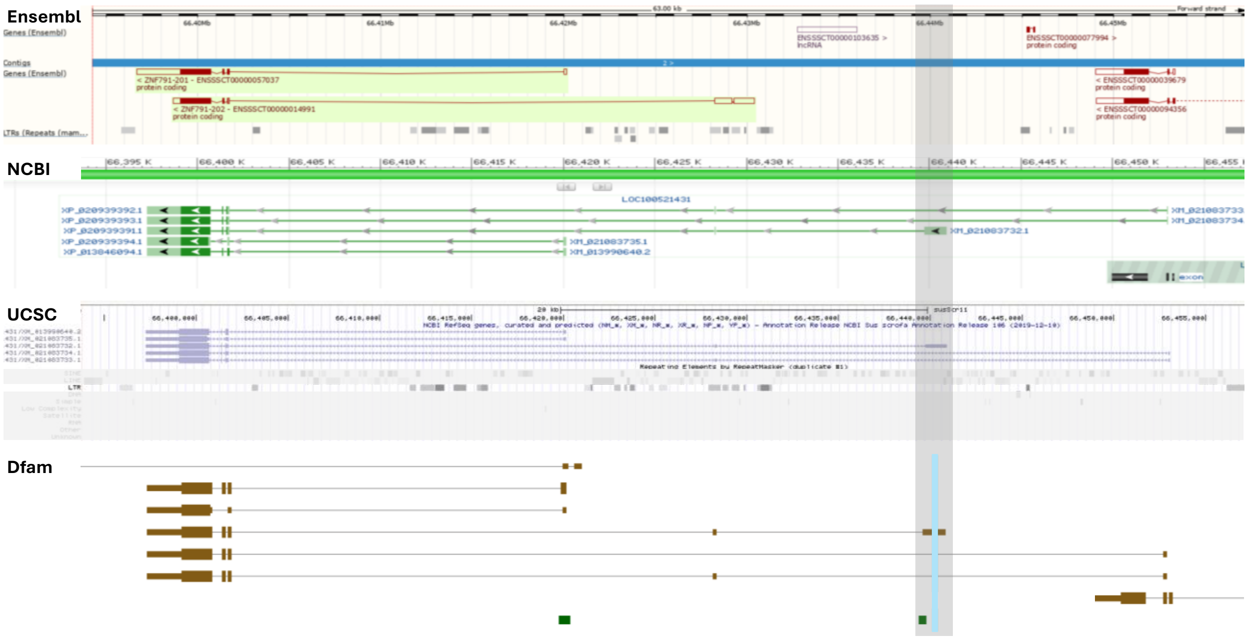

For the current pig genome assembly (susScr11), UCSC used RepeatMasker with the Repbase library to generate the LTR annotations. This annotation is based on the January 31,2015 version of RepeatMasker with the Repbase library release 20140131.

https://hgdownload.soe.ucsc.edu/goldenPath/susScr11/bigZips/

susScr11.fa.out.gz - RepeatMasker .out file. RepeatMasker was run with the
-s (sensitive) setting.
January 31, 2015 (open-4-0-5) version of RepeatMasker
RepBase library: RELEASE 20140131

In contrast, the Dfam annotations for the same assembly (susScr11) are more recent, with the latest update on November 16, 2023. The Dfam annotations leverage profile Hidden Markov Models (HMMs) and updated sequence models, potentially capturing more complex or diverged repetitive elements not detected by the older Repbase annotations. Additionally, Dfam uses RepeatModeler for *de novo* repeat detection.

https://www.dfam.org/releases/Dfam_3.8/annotations/DAsusScro11.1/

Index of /releases/Dfam_3.8/annotations/DAsusScro11.1/
DAsusScro11.1.hits.gz 16-Nov-2023 12:16 625133600
DAsusScro11.1.nrph.hits.gz 16-Nov-2023 12:16 126202935

Because LTRs may accumulate mutations over time and diverge substantially from the consensus sequence, it can be challenging to detect these elements using older versions of Repbase and RepeatMasker. In such cases, using recent versions of Dfam and RepeatModeler can improve detection accuracy and capture these divergent LTR sequences.

• Final point about the interest in pig production for feed efficiency (article of Li et al.)

That is true that the SNP rs81272049, located in the ZNF791 region was associated with RFI only in Yorkshire breed. However, this is one among a very long 8Mb haplotype including potential other imprinted genes. Consequently, this observation is important but might be moderated by suggesting that imprinted *ZNF791-like* gene belongs to a genomic regions which is associted with RFI phenotype.

Ans) We agree with the reviewer's point, and as mentioned in the Discussion section, further research is needed to investigate the functional relevance of the unannotated AS gene, which is closest to the reported SNP, as well as the role of this imprinted gene cluster (L468-471).

• L516 - supplementary Table 3

Ans) Thank you for pointing out this error (L590, 596, and 598). We have corrected it.

Have all data underlying the figures and results presented in the manuscript been provided?
Large-scale datasets should be made available via a public repository as described in the *PLOS Genetics* data availability policy, and numerical data that underlies graphs or summary statistics should be provided in spreadsheet form as supporting information.

Reviewer #1: Yes

Reviewer #2: Yes

Reviewer #3: Yes

PLOS authors have the option to publish the peer review history of their article (what does this mean?). If published, this will include your full peer review and any attached files.

If you choose “no”, your identity will remain anonymous but your review may still be made public.

Do you want your identity to be public for this peer review? For information about this choice, including consent withdrawal, please see our Privacy Policy.

Reviewer #1: **Yes:** Louis Lefebvre

Reviewer #2: No

Reviewer #3: No
