# Supplementary material for: Evolutionary lineage-specific genomic imprinting at the ZNF791 locus
Source: PLoS Genet. 2025 Jan 15;21(1):e1011532. doi: 10.1371/journal.pgen.1011532 (PMC11734915; doi:10.1371/journal.pgen.1011532)
Supplement: Attachment — Submitted filename: Response_to_Reviewers.docx [file pgen.1011532.s032.docx]

Dear Editors and Peer Reviewers,

Thank you for your positive feedback on our revised submission (PGENETICS-D-24-00906R1). In response to your additional suggestions, we have further revised the manuscript, with modifications highlighted in yellow in the lines described in our responses. Please find our responses, revision details, and an updated reference below.

**Editors' comments:**

From my side, I would like to suggest a small change in the Title to better reflect what this study is about: 'Evolutionary lineage-specific genomic imprinting at the ZNF791 gene'.  In fact, many studies have considered developmental lineage-specific imprinting, and to not confuse the reader and highlight the specificity of your study, it would be good to include the word 'evolutionary' into the title as suggested.

Ans) Thank you for your insightful comment. In response to the Editors’ suggestion, we have revised the title to 'Evolutionary lineage-specific genomic imprinting at the *ZNF791* locus'. We replaced “gene” with “locus” to better reflect that our study also considers the surrounding genomic regions, including an unannotated transcript and regulatory elements.

**Reviewers' comments:**

Reviewer's Responses to Questions

**Comments to the Authors:
Please note here if the review is uploaded as an attachment.**

Reviewer #1: I thank the authors for addressing my comments appropriately. I think this revised version is much improved.
However, two minor points still need to be addressed.

Lines 401-403 of the revised manuscript: “Alternatively, transcriptional interference can occur over enhancer domains when so-called Enhancer Occlusion Transcripts (EOTrs) occupy these regions.”

“Occupy” should be changed to “cover”

Ans) Thank you for your positive evaluation and additional comments. As suggested, we have replaced “occupy” with “cover” in line 403.

In response to my comment #14, the authors added lines 442-445 to discuss the implication of ZFP57/ZNF445 in DNA methylation maintenance at the ZNF791 DMR during preimplantation stages. However, they still need to specifically mention whether the imprinted ZNF791 DMR
contains binding motifs for these proteins.

Ans) We have revised lines 443-451 to address ZFP57/ZNF445 and their binding motifs on the imprinted ZNF791 DMRs. Additionally, we have included S23E Fig and S6 Table to present results of the motif analysis. A reference has also been added regarding the predicted ZNF445 binding motifs (ref [32]).

32.	Barazandeh M, Lambert SA, Albu M, Hughes TR. Comparison of ChIP-Seq Data and a Reference Motif Set for Human KRAB C2H2 Zinc Finger Proteins. G3 (Bethesda). 2018;8(1):219-29. Epub 2017/11/18. doi: 10.1534/g3.117.300296. PubMed PMID: 29146583; PubMed Central PMCID: PMCPMC5765350.

Reviewer #2: The authors have addressed all my comments in the revised manuscript.
The work is a nice and important contribution to the field of genomic imprinting.

Ans) Thank you for recognizing the significance of our work and its contribution to the field.

Reviewer #3: I'd like to thank the authors and congratulate them for taking all the comments into account and for their answers. The manuscript was already good, it's even better now and the results are very interesting for the scientific community!

Ans) Thank you for your thoughtful review. We greatly appreciate your encouraging feedback and recognition of our work.

**Have all data underlying the figures and results presented in the manuscript been provided?**
Large-scale datasets should be made available via a public repository as described in the *PLOS Genetics* [data availability policy](https://urldefense.com/v3/__http:/track.editorialmanager.com/CL0/http:*2F*2Fjournals.plos.org*2Fplosgenetics*2Fs*2Fdata-availability/1/010f01931b5f4fd5-3018f7da-b6b2-4275-9946-3fd04a98a1ac-000000/ZdpjpbRtoz11_7rvl0q9O-0a908hkHu3UscYHBh5XVw=184__;JSUlJSU!!KGKeukY!zXuUWOnBdBniXz_PsoxtL_eJHJnKumQRq_Gf0zlZGcwZS3PbGQjqUI2fxbjI2VA3r_g-a-18ocbs7tdwxQ$), and numerical data that underlies graphs or summary statistics should be provided in spreadsheet form as supporting information.

Reviewer #1: Yes

Reviewer #2: Yes

Reviewer #3: Yes

PLOS authors have the option to publish the peer review history of their article ([what does this mean?](https://urldefense.com/v3/__https:/track.editorialmanager.com/CL0/https:*2F*2Fjournals.plos.org*2Fplosgenetics*2Fs*2Feditorial-and-peer-review-process*23loc-peer-review-history/1/010f01931b5f4fd5-3018f7da-b6b2-4275-9946-3fd04a98a1ac-000000/Ud555T71K0mhcnq3Q0Xo2fL_YE7HqcpEMxA6redIEH4=184__;JSUlJSUl!!KGKeukY!zXuUWOnBdBniXz_PsoxtL_eJHJnKumQRq_Gf0zlZGcwZS3PbGQjqUI2fxbjI2VA3r_g-a-18ocYbJ51plQ$)). If published, this will include your full peer review and any attached files.


If you choose “no”, your identity will remain anonymous but your review may still be made public.


**Do you want your identity to be public for this peer review?** For information about this choice, including consent withdrawal, please see our [Privacy Policy](https://urldefense.com/v3/__https:/track.editorialmanager.com/CL0/https:*2F*2Fwww.plos.org*2Fprivacy-policy/1/010f01931b5f4fd5-3018f7da-b6b2-4275-9946-3fd04a98a1ac-000000/PBwRcgTAYeUqrVmzjPRD0Hn_q1k6KD1KaB2Nu9cE8-w=184__;JSUl!!KGKeukY!zXuUWOnBdBniXz_PsoxtL_eJHJnKumQRq_Gf0zlZGcwZS3PbGQjqUI2fxbjI2VA3r_g-a-18ocZLvisjSA$).

Reviewer #1: No

Reviewer #2: No

Reviewer #3: **Yes:** Julie Demars
